# Neosetophomone B induces apoptosis in multiple myeloma cells via targeting of AKT/SKP2 signaling pathway

**DOI:** 10.1002/cbin.12101

**Published:** 2023-10-26

**Authors:** Shilpa Kuttikrishnan, Fareed Ahmad, Jericha M. Mateo, Kirti S. Prabhu, Tamam El‐Elimat, Nicholas H. Oberlies, Cedric J. Pearce, Ammira S. Alshabeeb Akil, Ajaz A. Bhat, Feras Q. Alali, Shahab Uddin

**Affiliations:** ^1^ Translational Research Institute, Academic Health System Hamad Medical Corporation Doha Qatar; ^2^ College of Pharmacy, QU Health Qatar University Doha Qatar; ^3^ Dermatology Institute, Academic Health System Hamad Medical Corporation Doha Qatar; ^4^ Department of Medicinal Chemistry and Pharmacognosy, Faculty of Pharmacy Jordan University of Science and Technology Irbid Jordan; ^5^ Department of Chemistry and Biochemistry University of North Carolina at Greensboro Greensboro North Carolina USA; ^6^ Mycosynthetix Inc. Hillsborough North Carolina USA; ^7^ Department of Human Genetics‐Precision Medicine in Diabetes Obesity and Cancer Research Program, Sidra Medicine Doha Qatar; ^8^ Laboratory of Animal Research Center Qatar University Doha Qatar

**Keywords:** AKT, caspases, drug synergy, multiple myeloma, neosetophomone B, SKP2

## Abstract

Multiple myeloma (MM) is a hematologic malignancy associated with malignant plasma cell proliferation in the bone marrow. Despite the available treatments, drug resistance and adverse side effects pose significant challenges, underscoring the need for alternative therapeutic strategies. Natural products, like the fungal metabolite neosetophomone B (NSP‐B), have emerged as potential therapeutic agents due to their bioactive properties. Our study investigated NSP‐B's antitumor effects on MM cell lines (U266 and RPMI8226) and the involved molecular mechanisms. NSP‐B demonstrated significant growth inhibition and apoptotic induction, triggered by reduced AKT activation and downregulation of the inhibitors of apoptotic proteins and S‐phase kinase protein. This was accompanied by an upregulation of p21Kip1 and p27Cip1 and an elevated Bax/BCL2 ratio, culminating in caspase‐dependent apoptosis. Interestingly, NSP‐B also enhanced the cytotoxicity of bortezomib (BTZ), an existing MM treatment. Overall, our findings demonstrated that NSP‐B induces caspase‐dependent apoptosis, increases cell damage, and suppresses MM cell proliferation while improving the cytotoxic impact of BTZ. These findings suggest that NSP‐B can be used alone or in combination with other medicines to treat MM, highlighting its importance as a promising phytoconstituent in cancer therapy.

## 1 INTRODUCTION

Multiple myeloma (MM) is a rare cancer of the blood and bone marrow that triggers various symptoms, such as anemia, hypercalcemia, bone destruction, abnormal bleeding, and renal failure (Firth, 2019). While therapy has improved MM patients' life quality and expectancy, drug‐resistant clones often render most patients incurable, leading to decreased survival rates (Thorsteinsdottir et al., 2018; Yang & Lin, 2015). Thus, there is a pressing need for new treatments to combat this devastating disease (Li et al., 2021; Liu et al., 2021; Yang & Lin, 2015).

Natural compounds have played a significant role in developing anticancer drugs, with examples such as actinomycin D, etoposide, docetaxel, mitomycin C, bleomycin, paclitaxel, and vincristine (Kinghorn et al., 2016, 2009). Fungi's secondary metabolites have gained much attention as a potential source of new cytotoxic scaffolds, with meroterpenoids being structurally diverse and isolated from various sources such as fungi, marine organisms, animals, and plants (Kinghorn et al., 2016; Zhao et al., 2021). Neosetophomone B (NSP‐B), a meroterpenoid fungal secondary metabolite isolated from an unidentified *Neosetophoma* sp. (strain MSX50044), is cytotoxic in solid cancer cell lines at micromolar doses (El‐Elimat et al., 2019). However, the mechanism underlying NSP‐B‐mediated cytotoxicity remains unclear. The phosphatidylinositol 3‐kinase/AKT (PI3K/AKT) signaling pathway and its downstream effectors play a critical role in oncogenesis and are frequently activated in many cancers (Cantley, 2002; Geyer et al., 2018). AKT activation stimulates antiapoptotic signaling by activating NF‐kappa B, Bad, GSK3, forkhead box 1, and inhibitors of apoptotic proteins (IAPs; Cardone et al., 1998; Cross et al., 1995; Datta et al., 1997; Uddin et al., 2008). S‐phase kinase‐associated protein 2 (SKP2) is a component of the SCF (SKP1‐cullin‐F‐box) E3 ubiquitin ligase complex, which targets proteins for degradation. The role of SKP2 in cellular processes like cell cycle progression, apoptosis, cellular proliferation, and cellular differentiation makes it a significant player in tumorigenesis and cancer progression (Uddin et al., 2016). Numerous studies have shown that SKP2 is overexpressed in many human cancers, including prostate, breast, lung, colorectal, and gastric cancers, as well as lymphomas, melanomas, and sarcomas. Overexpression of SKP2 is associated with poor prognosis in many of these cancers (Gstaiger et al., 2001; Radke et al., 2005). SKP2 plays a critical role in the regulation of the G1/S phase transition of the cell cycle by promoting the degradation of cell cycle inhibitors such as p27Kip1 and p21Cip1 (Tsvetkov et al., 1999). Thus, aberrant expression of SKP2 can lead to uncontrolled cell proliferation, a key characteristic of cancer. SKP2 can degrade the tumor suppressor p53 and other proteins involved in the inhibition of cell growth (Wei et al., 2004). Inhibiting SKP2 function could stabilize these tumor suppressor proteins and inhibit cancer cell growth. SKP2 can influence the epithelial–mesenchymal transition, a process through which cancer cells acquire invasive and metastatic properties. Inhibition of SKP2 could potentially suppress these processes (Lin et al., 2010). SKP2 has been implicated in resistance to chemotherapy, and targeting SKP2 could potentially restore the sensitivity of cancer cells to these treatments (Chan et al., 2010). The AKT/SKP2 signaling pathway is known to play a crucial role in cell survival, proliferation, and apoptosis (Kulinski et al., 2018). Overactivation of this pathway is seen in several cancer types, including MM, and it promotes disease progression and resistance to treatment (Li et al., 2006). AKT activation leads to phosphorylation and stabilization of SKP2, which in turn promotes cell cycle progression and survival (Chan et al., 2012). Given the evidence suggesting an aberrant AKT/SKP2 pathway in MM and the antiproliferative effect of NSP‐B, it is plausible to hypothesize that NSP‐B may exert its therapeutic effect via the AKT/SKP2 pathway. This could involve NSP‐B‐induced downregulation of AKT phosphorylation and SKP2 expression, which may inhibit cell proliferation and induce apoptosis in MM cells.

In this study, we aimed to investigate NSP‐B's potential to inhibit tumor growth in MM cell lines. Our results demonstrate that NSP‐B treatment of MM cells decreased cell viability by inducing apoptosis. Furthermore, NSP‐B suppressed the AKT/SKP2 signaling axis and its downstream substrate molecules, including IAPs. Interestingly, NSP‐B enhanced the anticancer effects of bortezomib (BTZ) on MM cells.

## 2 MATERIALS AND METHODS

### 2.1 Isolation of the fungal compound NSP‐B

NSP‐B was isolated from *Neosetophoma* sp. (strain MSX50044). Solid‐phase cultures of *Neosetophoma* sp. were grown on rice and extracted using CHCl_3_−MeOH (1:1). The crude extracts were fractionated using normal‐phase flash chromatography and purified using preparative high‐performance liquid chromatography. High‐resolution electrospray ionisation mass spectroscopy and nuclear magnetic resonance were used to determine the structure of NSP‐B. X‐ray crystallography diffraction was also used to corroborate the structure. The purity of NSP‐B was determined to be greater than 97% using ultra‐performance liquid chromatography chromatograms (El‐Elimat et al., 2019).

### 2.2 Cell culture

U266 and RPMI8226 cells were purchased from ATCC. RPMI 1640 media supplemented with 10% (vol/vol) fetal bovine serum (FBS), 100 U/mL penicillin, and 100 U/mL streptomycin was used to grow cells, as previously described, at 37°C in a humid environment containing 5% CO_2_ (Kuttikrishnan, Bhat, et al., 2022).

### 2.3 Reagents and antibodies

Cell Counting Kit‐8 (CCK‐8) was purchased from Sigma Aldrich. z‐VAD‐fmk and BTZ were purchased from Tocris. Antibodies against SKP2, P21, P27, CDK‐4, CDK‐6, caspase‐9, cleaved caspase‐3, caspase‐3, Bid, Poly(ADP‐ribose) polymerases (PARP), cleaved caspase 8, cleaved caspase 9, XIAP, CIAP1 and 2, P‐AKT, AKT, ubiquitin, cyclin D1 and B1 were purchased from Cell Signaling Technologies. Antibodies against HSP60, p‐H2AX, Bax, cytochrome c, and Bcl2 were purchased from Santa Cruz Biotechnology Inc. FBS, penicillin–streptomycin, live and dead assay kit, and RPMI 1640 medium were purchased from Life Technologies Inc. Fluorescein isothiocyanate (FITC) Annexin V apoptosis detection kit I, Apo‐Direct kit, fixation/permeabilization solution kit, BD MitoScreen (JC‐1), BV421mouse anti‐p‐H2AX (pS139), PE rabbit antiactive caspase‐3, and Alexa Fluor 700 mouse anticleaved PARP (Asp214) antibodies were purchased from BD Biosciences.

### 2.4 Cell viability assay

NSP‐B's antiproliferative activity on myeloma cells was evaluated using CCK‐8. A total of 1 × 10^4^ cells/well were seeded in a 96‐well plate with varying concentrations of NSP‐B (from 0 to 40 μM) for 48 h. After incubation, 10 µL of CCK‐8 was added to each well, and the plates were incubated at 37°C to develop color. Finally, the microplate reader (Tecan Bio Tek Instruments Inc.) was used to measure the optical density (OD, absorbance) at 450 nm. The percentage of the viable cells was calculated as the OD of the experiment samples/OD of the control sample ×100 (Akhtar et al., 2019).

### 2.5 Live/dead assay

U266 and RPMI8226 cells were treated with escalating doses of NSP‐B, and then incubated for 48 h. The live and dead stain was prepared by adding 5 µL of calcein AM (Component A) and 20 µL of ethidium homodimer‐1 (Component B) to 10 mL of phosphate buffer saline (PBS). The cells were stained with this solution for 15–30 min at room temperature in the dark. The EVOS FLoid Cell Imaging System; Invitrogen (Thermo Fisher Scientific), was used to acquire the images at 20Χ magnification (Kuttikrishnan, Bhat, et al., 2022; Shishodia et al., 2007).

### 2.6 Measurement of apoptosis

NSP‐B‐induced apoptosis was confirmed using the Annexin V/propidium iodide (PI) dual staining assay. Briefly, 2 × 10^6^ cells were treated with NSP‐B for 48 h before being harvested, rinsed in ice‐cold PBS, and stained with Annexin V‐FITC and PI in 1X binding buffer for 20 min. Using flow cytometry and the BD LSR Fortessa analyzer (BD Biosciences), the cells were categorized as live (Annexin FITC negative, PI negative), early apoptotic (Annexin FITC positive, PI negative), late apoptotic (Annexin FITC positive, PI positive), or necrotic (Annexin FITC negative and PI positive). The apoptosis percentage was computed by combining the early and late apoptotic cell percentages (Prabhu et al., 2021).

### 2.7 Cell cycle analysis

U266 and RPMI8226 cells were treated with NSP‐B for 48 h. The cells were then exposed for 30 min at 37°C to Hoechst 33342 fluorescent stain in a complete medium. Flow cytometry analyzed the cell cycle distribution using the BD LSR Fortessa analyzer (BD Biosciences), as previously mentioned (Al‐Tamimi et al., 2022).

### 2.8 Immunoblotting technique

U266 and RPMI8226 cells were treated with NSP‐B, BTZ, and z‐VAD‐fmk for 48 h as previously described. Sodium dodecyl‐sulfate polyacrylamide gel electrophoresis (SDS‐PAGE) was used to separate proteins and transferred to a polyvinylidene difluoride (PVDF) membrane (Biorad). Cell lysates were prepared, and the protein quantification was performed using the ND‐1000 spectrophotometer (Nanodrop Technologies, Thermoscientific). A total of 50–100 µg of protein were loaded and separated in SDS‐PAGE and transferred into a PVDF membrane (Biorad). Several antibodies were utilized for immunoblotting, and a ChemiDoc system (Bio‐Rad) was used to generate and visualize the blots) (Akhtar et al., 2022).

### 2.9 Flow cytometric analysis of active caspase 3 and cleaved PARP

After 48 h of treatment with NSP‐B at varying lower doses (0.1, 0.5, 1, 2.5, and 5 µM), cells were fixed and permeabilized using the BD Cytofix/Cytoperm plus fixation and permeabilization solution kit. Anti‐active Caspase‐3‐BV605 and PARP Cleaved Form‐AF700 antibodies were used to stain 0.3 × 10^6^ cells in HBS Hank's balanced salt solution (HBSS) for 30 min. The cells were resuspended in the same solution after being rinsed once more with HBS. Flow cytometry using a BD LSR Fortessa analyzer was used to determine the percentage of cells with activated caspase‐3 and cleaved PARP (Iskandarani et al., 2016).

### 2.10 Quantitation of DNA double‐strand breaks

MM cells were treated with NSP‐B with lower doses for 48 h. After incubation, the cells were fixed and permeabilized using a BD Cytofix/Cytoperm plus fixation and permeabilization solution kit, as per the manufacturer's instructions. H2AX (pS139)‐Alexa Fluor 647 antibody was used to stain 0.3 × 10^6^ cells in HBSS for 30 min at room temperature. Using a BD LSRFortessa analyzer, DNA damage was measured by flow cytometry and quantified (Iskandarani et al., 2016).

### 2.11 Measurement of mitochondrial membrane potential (MMP)

For 48 h, U266 and RPMI8226 cells were treated with NSP‐B at various doses. The membrane‐permanent JC‐1 was used to stain the cells, and flow cytometry on a BD LSR Fortessa analyzer (BD Biosciences) was used to measure the potential of the mitochondrial membrane (Prabhu et al., 2017).

### 2.12 Gene silencing

Using the 4D‐Nucleofector^TM^ System (Lonza), 1 × 10^6^ U266 cells were transfected with AKT small interfering RNA (siRNA; Qiagen) and control siRNA (Cat no: 1027281; Qiagen). Upon completion of the 48‐h incubation period, cells were lysed and immunoblotted with AKT and other antibodies (Akhtar et al., 2019).

### 2.13 Statistical analysis

One‐way analysis of variance and Tukey's multiple comparison test were used to compare between the groups. The data were statistically analyzed using GraphPad Prism software (version 5.0 for Windows; GraphPad Software Inc., http://www.graphpad.com). The data are presented as a mean standard deviation. Values of *p ≤ .05, **p ≤ .01, and ***p ≤ .001 are statistically significant. Denisitometric analysis was carried out for all the western blots using ImageJ software.

## 3 RESULTS

### 3.1 NSP‐B reduces the viability and induces apoptosis in MM cells

**Figure 1:**
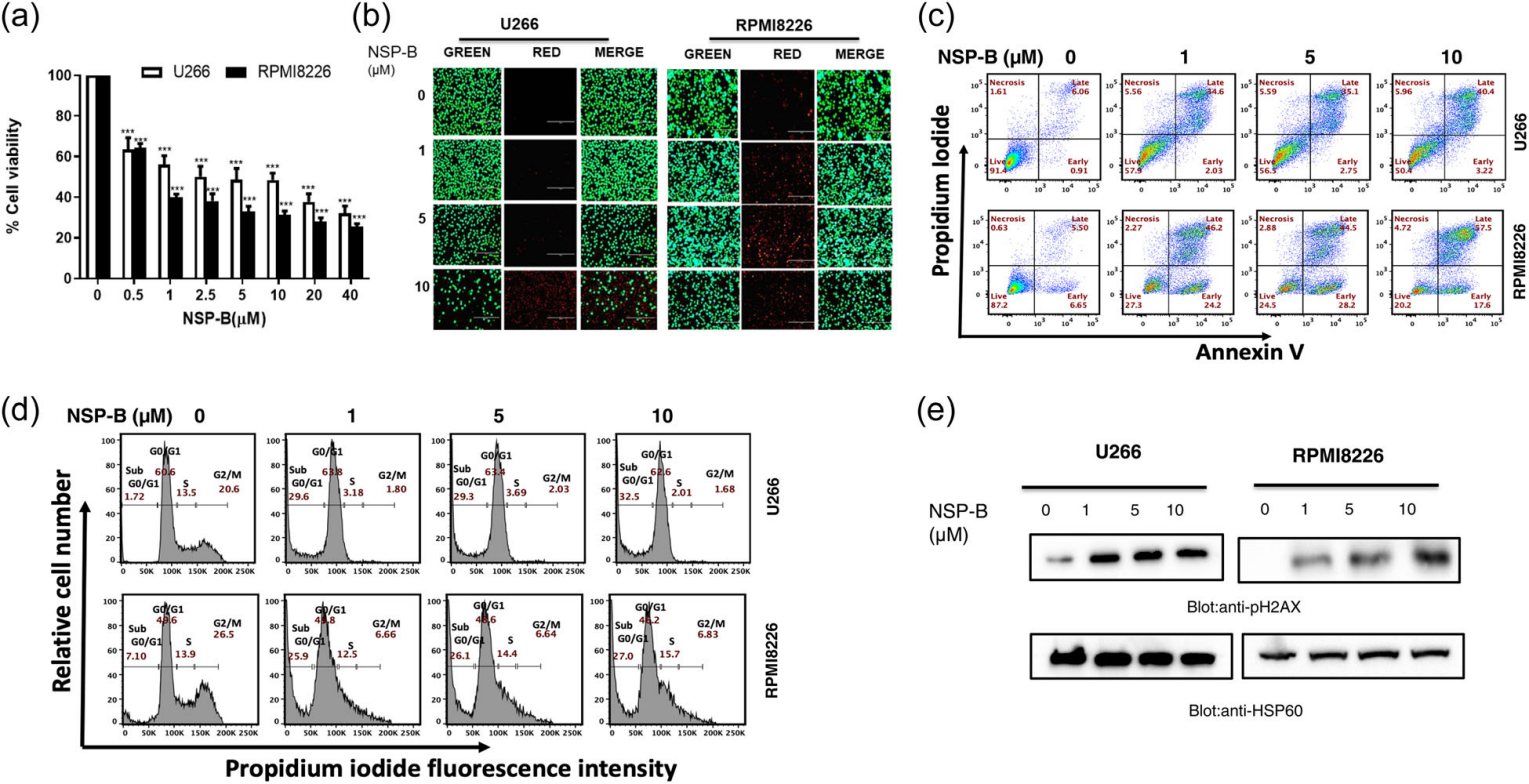
Effect of NSP‐B on cell viability of MM cells. (a) NSP‐B inhibits the cell viability of MM cells. U266 and RPMI8226 cells were incubated for 48 h with the indicated concentrations of NSP‐B (0.5–40 µM). Cell viability assays were performed using CCK‐8 as mentioned in Section 2. The graph displays the mean ± SD of three independent experiments. *p < .05, **p < .01,***p < .001. (b) NSP‐B induces cell death in MM cell lines. U266 and RPMI8226 cells were treated with doses 1, 5, and 10 µM of NSP‐B for 48 h. Then the cells were stained with live and dead reagent and visualized under a fluorescent microscope. (c) NSP‐B‐mediated apoptosis in MM cells. U266 and RPMI8226 cells were treated with NSP‐B (1, 5, and 10 µM), followed by staining with fluorescein‐conjugated Annexin‐V/PI, and apoptotic cells were determined by flow cytometry. (d) Effect of NSP‐B on cell cycle distribution. U266 and RPMI8226 cells were treated with NSP‐B, and cell cycle fractions were determined with flow cytometry as described in the method and materials. (e) NSP‐B‐mediated phosphorylation of H2AX in MM cells. U266 and RPMI8226 cells were treated with NSP‐B, and pH2AX level was determined by Western blot analysis using antibodies against p‐H2AX and HSP60. Original Western blots, microscopic images and quantification graphs can be found at Files S1 and S2. CCK‐8, Cell Counting Kit‐8; MM, multiple myeloma; NSP‐B, neosetophomone B; PI, propidium iodide.

We investigated the potential of NSP‐B to inhibit the viability of U266 and RPMI8226 MM cells. The cells were treated with various concentrations of NSP‐B (0.5, 1, 2.5, 5, 10, 20, and 40 µM) for 48 h, and cell viability was measured using the CCK‐8 assay. We observed a dose‐dependent reduction in cell growth in both cell lines, with most concentrations resulting in statistically significant growth suppression (Figure 1a). To determine if this reduction in cell viability was due to cell death, live/dead assays were performed. The proportion of dead cells increased in a dose‐dependent manner following NSP‐B treatment (Figures 1b and S1b). We also evaluated whether NSP‐B induced apoptosis in MM cells by treating U266 and RPMI8226 cells with experimental doses of NSP‐B for 48 h, and then staining with Annexin V. Significant apoptosis was observed at doses of 1 μM and higher (Figure 1c). The combined apoptosis percentages for U266 cell line are 36.63% at 1 μM, 37.85% at 5 μM, and 43.62% at 10 μM, and for RPMI8226 cell line are 70.4% at 1 μM, 72.7% at 5 μM, and 75.1% at 10 μM. Similarly, significant apoptosis was observed at doses 0.5 μM and higher (Figures S1a,b and S5a,b). Flow cytometry was used to analyze cell cycle distribution, and we found that the percentage of cells in the SubG0/G1 phase was significantly higher after 48 h of NSP‐B treatment compared to controls. The SubG0 fractions for the U266 cell line are 1.72% for untreated cells, 29.6% at 1 μM, 29.3% at 5 μM and 32.5% at 10 Μm of NSP‐B, and for RPMI8226 cell line are 7.10% for untreated cells, 25.9% at 1 μM, 26.1% at 5 μM, and 27.0% at 10 Μm of NSP‐B (Figure 1d). Moreover, NSP‐B treatment led to a dose‐dependent upregulation of p‐H2AX expression, a hallmark of double‐stranded breaks (Figure 1e), which was further confirmed in western blot analysis and flow cytometry in lower doses (Figures S2a–c and S5c,d).

### 3.2 NSP‐B activates intrinsic and extrinsic apoptotic pathways in MM cells

**Figure 2:**
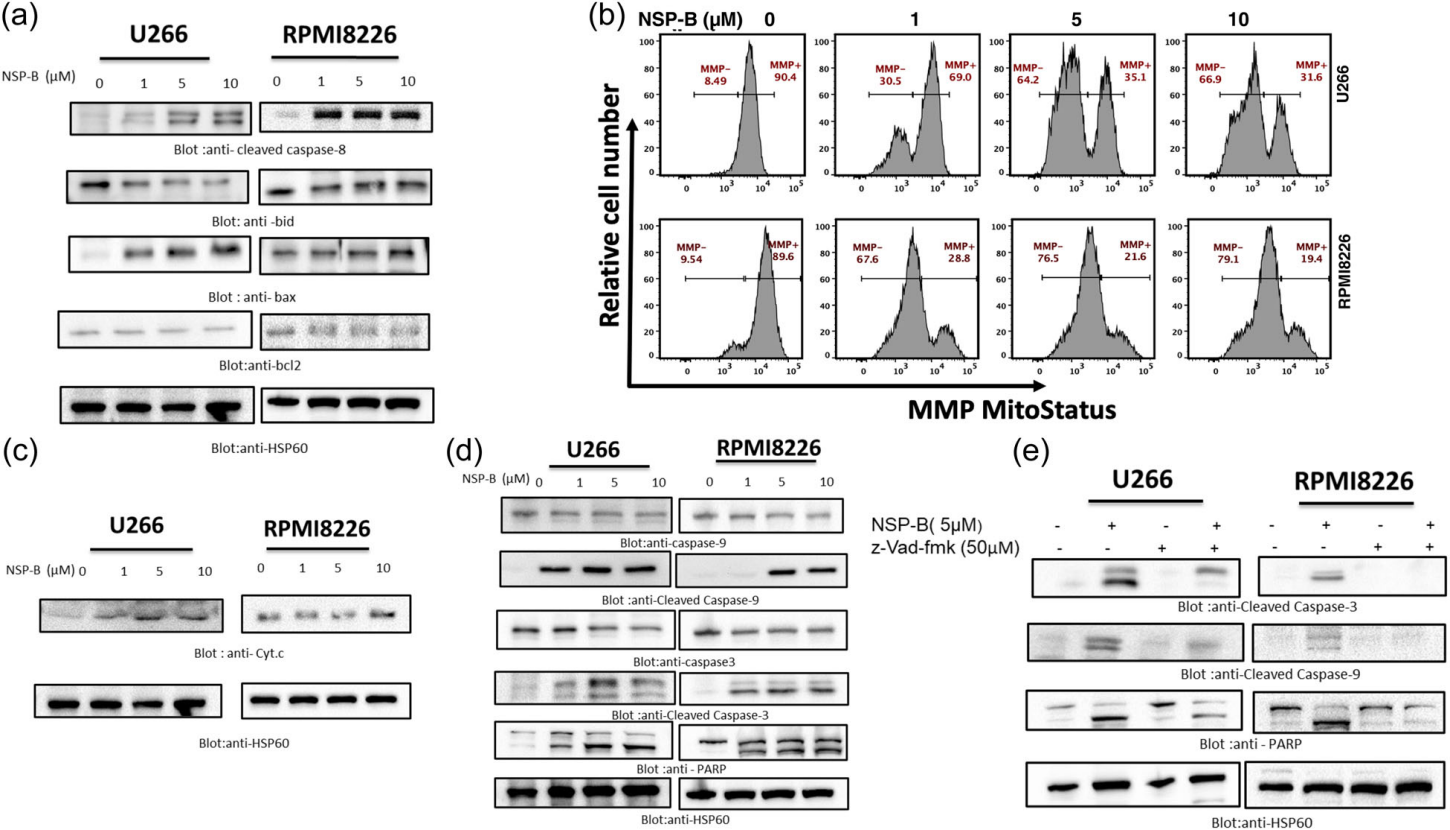
NSP‐B‐induced mitochondrial signaling pathways in leukemic cell lines. (a) Effect of NSP‐B on caspase‐8/BID/Bcl2 and Bax expression in MM cell lines. U266 and RPMI8226 cells were treated with NSP‐B as indicated, and 50 µg of protein was separated by SDS‐PAGE and immunoblotted with antibodies against cleaved caspase‐8, Bid, Bax, Bcl2, and HSP60 as indicated. (b) NSP‐B‐mediated loss of mitochondrial membrane potential in MM cells. U266 and RPMI8226 cells were treated with indicated doses (1, 5, 10 µM) of NSP‐B for 48 h. Mitochondrial membrane potential was determined by flow cytometry as described in the materials and methods section and analyzed. (c) The NSP‐B‐induced the release of cytochrome c. For U266 same blot of (a) was stripped and probed against antibody cytochrome c. Same HSP60 was used for (a) and (c). RPMI8226 cells were treated with NSP‐B, and proteins were separated on SDS‐PAGE and immunoblotted with antibodies against cytochrome c and HSP60 (d) NSP‐B mediates caspase activation in MM cells.U266 and RPMI8226 cells were treated with NSP‐B, and proteins were separated on SDS‐PAGE and immunoblotted with antibodies against caspase‐9, cleaved caspase‐9, caspase‐3, cleaved caspase‐3, PARP, and HSP60. (e) Effect of z‐Vad‐fmk on NSP‐B induced apoptosis. U266 and RPMI8226 cells were pretreated with z‐Vad‐fmk for 1 h, followed by NSP‐B treatment, and analyzed by western blot for antibodies against cleaved caspase‐3, cleaved caspase 9, PARP, and HSP60. For U266 same blot of (e) was stripped and probed against antibodies PARP and HSP60. Original Western blot images and quantification graphs can be found at Files S1 and S2. MM, multiple myeloma; NSP‐B, neosetophomone B; PARP, poly(ADP‐ribose)polymerases; SDS‐PAGE, sodium dodecyl‐sulfate polyacrylamide gel electrophoresis.

In this study, we investigated the effects of NSP‐B on caspase‐8 signaling and mitochondrial integrity in MM cells (Kuttikrishnan et al., 2019). Our results indicate that NSP‐B likely activates the extrinsic apoptotic signaling pathway and triggers Bid truncation via caspase‐8 activation, leading to increased apoptosis in U266 and RPMI8226 cells (Figure 2a). We also found that NSP‐B treatment upregulated Bax expression and downregulated Bcl2 expression in these cells, which may contribute to the proapoptotic effects of NSP‐B (Figure 2a). Furthermore, we observed a decrease in MMP in NSP‐B‐treated cells, which may suggest that NSP‐B modulates mitochondrial integrity (Figure 2b). Additionally, NSP‐B treatment led to the release of cytochrome c from the mitochondria, which triggered the activation of caspase‐9, ‐3, and PARP in a dose‐dependent manner in experimental doses (Figure 2c,d) as well as lower doses (Figures S3a–d, S4, 6a–d, S7a,b). Notably, the global caspase inhibitor z‐Vad‐fmk was able to block NSP‐B's effect on cleaved caspase‐3 and ‐9 and PARP (Figure 2e), indicating that NSP‐B triggers caspase‐cascade signaling during apoptosis in MM cells.

### 3.3 NSP‐B inhibits AKT activation, suppresses SKP2 and enhances p21, P27 expression in MM cells

**Figure 3:**
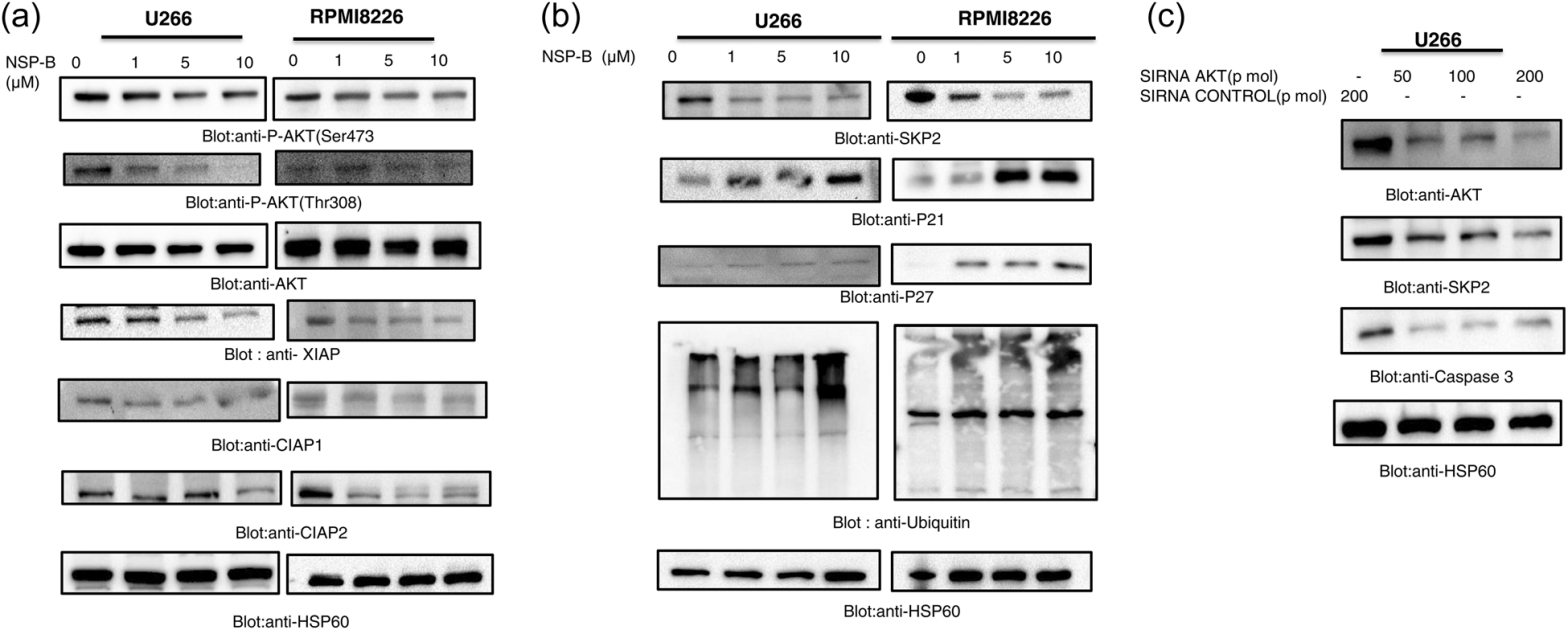
NSP‐B inhibits activated AKT/PKB, and induces downregulation of antiapoptotic proteins and SKP2 in MM cells. (a) NSP‐B treatment caused the inactivation of AKT (both Ser473 and Thr308) and downregulated the expression of antiapoptotic proteins. U266 and RPMI8226 cells were treated with various doses of NSP‐B. After cell lysis, equal amounts of proteins were separated by SDS‐PAGE, transferred to the PVDF membrane, and immunoblotted with antibodies against XIAP, c‐IAP1, c‐IAP2 and HSP60 as indicated. (b) NSP‐B treatment caused the downregulation of SKP2 and enhanced the expression levels of P27, P21, and ubiquitin. U266 and RPMI8226 cells were treated with various doses of NSP‐B, and equal amounts of proteins were immunoblotted with antibodies against SKP2, p27, p21, ubiquitin, and HSP60 as indicated. (c) Gene silencing of AKT suppressed SKP2 expression. U266 cells were transfected with control (200 pM) and AKT siRNA (50, 100, and 200 pM) as indicated in Section 2. Immunoblot analysis of U266 cells transfected with control (200 pM) and AKT siRNA (50, 100, and 200 pM). Cells were lysed, and an equal amount of proteins for each sample were loaded onto the SDS‐polyacrylamide gel. Membranes were blotted against AKT, SKP2, caspase 3, and HSP60. Original Western blot images and quantification graphs can be found in Files S1 and S2. MM, multiple myeloma; NSP‐B, neosetophomone B; PVDF, polyvinylidene difluoride; SDS‐PAGE, sodium dodecyl‐sulfate polyacrylamide gel electrophoresis; siRNA, small interfering RNA. MM, multiple myeloma; NSP‐B, neosetophomone B.

We investigated the effects of NSP‐B on the AKT/PKB pathway in U266 and RPMI8226 cells by treating them with increasing concentrations of NSP‐B (0, 1, 5, and 10 µM). The results showed that NSP‐B caused a dose‐dependent dephosphorylation of AKT at Ser473 and Thr308 without altering total AKT expression (Figure 3a). Furthermore, NSP‐B treatment led to a reduction in the levels of inhibitors of apoptosis protein (IAP), including XIAP, cIAP1, and cIAP2 in both U266 and RPMI8226 cells (Figure 3a). In addition, NSP‐B decreased SKP2 expression while increasing p21Cip1 and p27/kip1 levels (Figures 3b and S7a,b), suggesting a mechanism for NSP‐B‐induced apoptosis through the inhibition of AKT and SKP2 signaling.

To further confirm the involvement of AKT in NSP‐B‐induced apoptosis, we used siRNA to silence the AKT gene in U266 cells. AKT‐specific siRNA was transfected into the cells using a Lonza nucleofector system, and the levels of AKT, SKP2, caspase‐3, and HSP60 were measured using various antibodies. The results showed that knocking down the AKT gene reduced SKP2 expression and caspase‐3 levels (Figure 3c), suggesting that AKT inhibition inhibits cell growth and induces apoptosis in MM cells by downregulating SKP2.

### 3.4 NSP‐B causes the inhibition of cyclins and cyclin‐dependent kinases (CDKs) in MM cells

**Figure 4:**
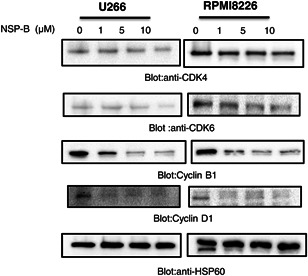
NSP‐B inhibits the cyclins and CDKs in MM cells. (a) NSP‐B treatment caused the downregulation of CDK‐4 and ‐6 and cyclins B1 and D1. U266 and RPMI8226 cells were treated with various doses of NSP‐B, and equal amounts of proteins were immunoblotted with antibodies against CDK‐4, CDK‐6, cyclin B1, cyclin D1, and HSP60 as indicated. Original Western blot images and quantification graphs can be found in Files S1 and S2. CDK, cyclin‐dependent kinase; MM, multiple myeloma; NSP‐B, neosetophomone B.

Our study aimed to examine the effects of NSP‐B treatment on the expression of cyclins and CDKs in MM cells. Following 48 h of treatment with NSP‐B, the expression of cyclin B1 and D1, as well as the cyclin‐dependent kinases CDK‐6 and ‐4, was observed to decrease dose‐dependent (Figure 4).

### 3.5 Cotreatment with NSP‐B and BTZ enhances cytotoxicity in MM cells

**Figure 5:**
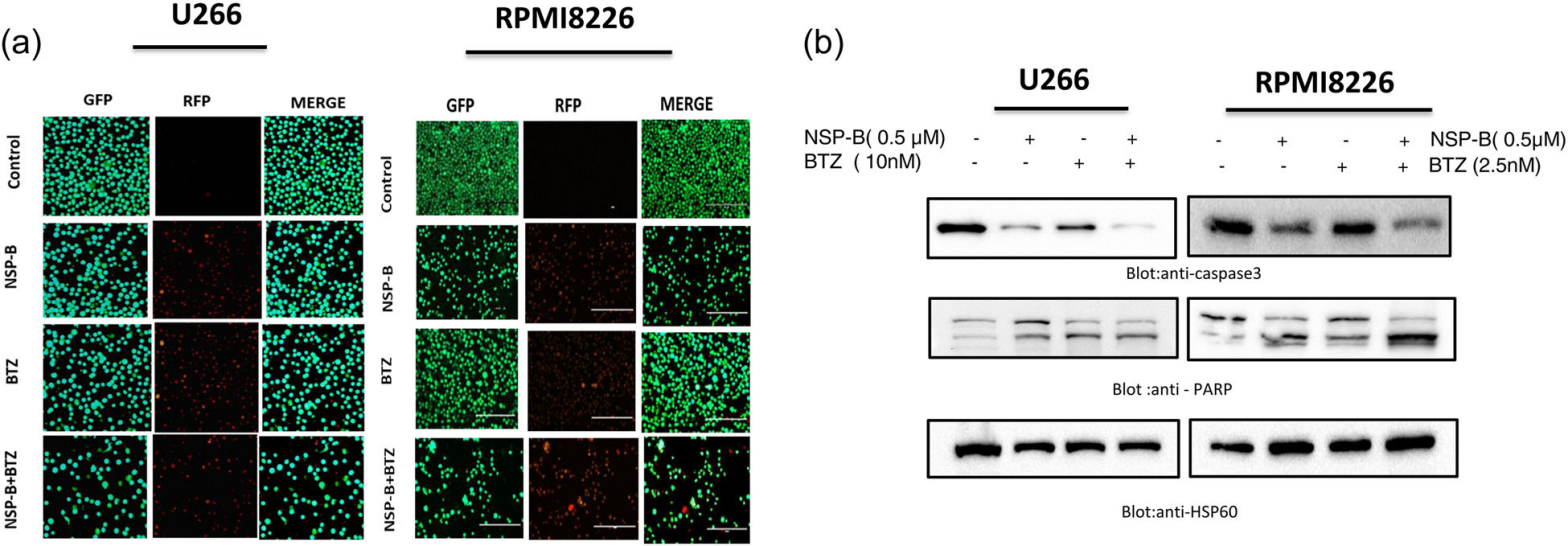
Combination of NSP‐B and BTZ augmented the inhibition of cell viability and induces apoptosis in myeloma cells. (a) U266 and RPMI8226 were treated with NSP‐B and BTZ alone and in combination for 48 h. After incubation, the cells were stained with live and dead reagent and visualized under a fluorescent microscope. (b) U266 and RPMI8226 were treated with 0.5 μM of NSP‐B and 10 nM BTZ for U266 and 2.5 nM BTZ for RPMI8226 alone and in combination. Cells were lysed and separated using SDS‐PAGE, transferred to a PVDF membrane, and immunoblotted with antibodies such as caspase 3, PARP, and HSP60. Original Western blots, microscopic images, and quantification graphs can be found at Files S1 and S2. BTZ, bortezomib; NSP‐B, neosetophomone B; PARP, poly(ADP‐ribose)polymerases; PVDF, polyvinylidene difluoride; SDS‐PAGE, sodium dodecyl‐sulfate polyacrylamide gel electrophoresis.

We proceeded to identify the subtoxic doses of NSP‐B and BTZ for their anticancer efficacy on myeloma cells. BTZ, a Food and Drug Administration (FDA)‐approved proteasome inhibitor, is commonly used to treat refractory MM (Iskandarani et al., 2016). However, its effectiveness is reduced by developing resistance and relapse (Murray et al., 2014; Zaal et al., 2017). We treated U266 and RPMI8226 cells with varying doses of NSP‐B and BTZ to determine their cytotoxic effects. Interestingly, subtoxic doses of NSP‐B (0.5 μM) and BTZ (10 nM for U266 and 2.5 nM for RPMI8226) significantly decreased cell viability in both cell lines after 48 h, as determined by a live and dead assay kit (Figures 5a and S5a). Furthermore, the combination treatment activated caspase‐3 and PARP, resulting in apoptosis, as evidenced by Western blot analysis results (Figure 5b).

## 4 DISCUSSION

Over the years, natural compounds have emerged as a promising approach for treating various malignancies, including cancer. These bioactive compounds act by targeting different signaling molecules and pathways, and growing evidence shows they can boost chemotherapy's effectiveness (Boulos et al., 2019; Mondal et al., 2019; Yuan et al., 2017). Fungal‐derived natural compounds, in particular, have been found to be extremely useful in the pharmaceutical industry, with several showing anticancer activity against cancer cell lines (Bills & Gloer, 2016; El‐Elimat et al., 2021; Kuttikrishnan, Prabhu et al., 2022; Prabhu et al., 2019). One such compound is NSP‐B, a fungal meroterpenoid secondary metabolite derived from *Neosetophoma* sp., which has been found to have anticancer action against various cancer cell lines, including leukemic cells (El‐Elimat et al., 2019; Kuttikrishnan, Bhat, et al., 2022; Kuttikrishnan, Masoodi, et al., 2022).

Our study found that NSP‐B induces apoptosis, also known as programmed cell death, in leukemic cells by triggering mechanisms such as the extrinsic receptor and intrinsic mitochondrial pathways (Elmore, 2007; Vasilikos et al., 2017). By increasing the Bax/Bcl2 ratio, NSP‐B triggers the release of cytochrome c into the cytoplasm, which produces an apoptosome complex that activates procaspase 9 and provides the PARP cleavage signal via activated caspase‐3 (Duriez & Shah, 1997). NSP‐B dose‐dependently cleaved caspase‐9, caspase‐3, and PARP, initiating the intrinsic caspase‐mediated apoptosis pathway, and induced the DNA damage marker H2AX, leading to DNA degradation and apoptosis (Oben et al., 2017).

The constitutive activation of PI3‐kinase/AKT signaling in malignant cells contributes to resistance to apoptosis (Tang et al., 2018). Aberrant activation of PI3‐kinase/AKT signaling has been observed in multiple human cancers (Dummler & Hemmings, 2007; Manning & Cantley, 2007). SKP2, an F‐box protein, is an oncogene and enhances cancer cell growth (Wang et al., 2020). Overexpression of SKP2 and high levels of AKT activity have been observed in numerous cancers (Kuttikrishnan, Prabhu, et al., 2022). Akt regulates SKP2 by promoting messenger RNA transcription, protecting it from APC/Cdh1‐mediated proteolysis, facilitating the formation of SKP2‐SCF complexes, and improving its cytoplasmic transport (Cai et al., 2020). In contrast, blocking AKT activity prevents SKP2 translocation (El‐Elimat et al., 2021). Our study found that NSP‐B inhibits constitutively active AKT and downregulates AKT‐regulated antiapoptotic proteins, including XIAP, cIAP1, and cIAP2. Treatment with NSP‐B leads to the dephosphorylation of AKT and downregulation of SKP2, increasing the expression of p27, p21, and ubiquitin in MM cells. These findings suggest that NSP‐B's anticancer effects on MM cells are due to its selective inhibition of the AKT/SKP2 signaling pathway. Inhibition of AKT activity using AKT siRNA leads to decreased SKP2 expression, caspase activity, and ultimately cell death. Cyclins and CDKs play a crucial role in regulating cell cycle progression. Vermeulen et al. (2003). In this study, we investigated the effect of NSP‐B on the expression of cyclins and CDKs, which are commonly overexpressed in various cancers (Shapiro, 2006). Our results showed that treatment with NSP‐B led to a dose‐dependent decrease in CDK‐6 and ‐4 expression and cyclins B1 and D1 in U266 and RPMI8226 cells. In addressing drug resistance in cancer treatment, emerging research posits a potential solution in repurposing FDA‐approved chemotherapeutic agents for use against various malignancies when amalgamated with other drugs or inhibitors (Chakravarty et al., 2022; Goel et al., 2021; Ramisetty et al., 2023; Scuoppo et al., 2019). Collectively, these studies illuminate the versatility and adaptability of existing chemotherapeutic drugs when paired with targeted inhibitors, opening novel therapeutic avenues in our relentless pursuit of effective cancer treatments. In the present study, we evaluated the potential for combination therapy with sub‐toxic concentrations of NSP‐B and BTZ, an FDA‐approved treatment for MM, to elicit a synergistic or additive effect that selectively kills tumor cells without harming healthy cells. Our results demonstrated that the combination of NSP‐B and BTZ reduced the viability of U266 and RPMI8226 cells and induced apoptosis more effectively than either drug alone. NSP‐B sensitized MM cells to BTZ, significantly reducing cell proliferation and enhancing cell death. These findings suggest that the use of sub‐toxic doses of NSP‐B in combination with BTZ may offer a promising strategy for improving the therapeutic outcomes of MM treatment.

## 5 CONCLUSION

**Figure 6:**
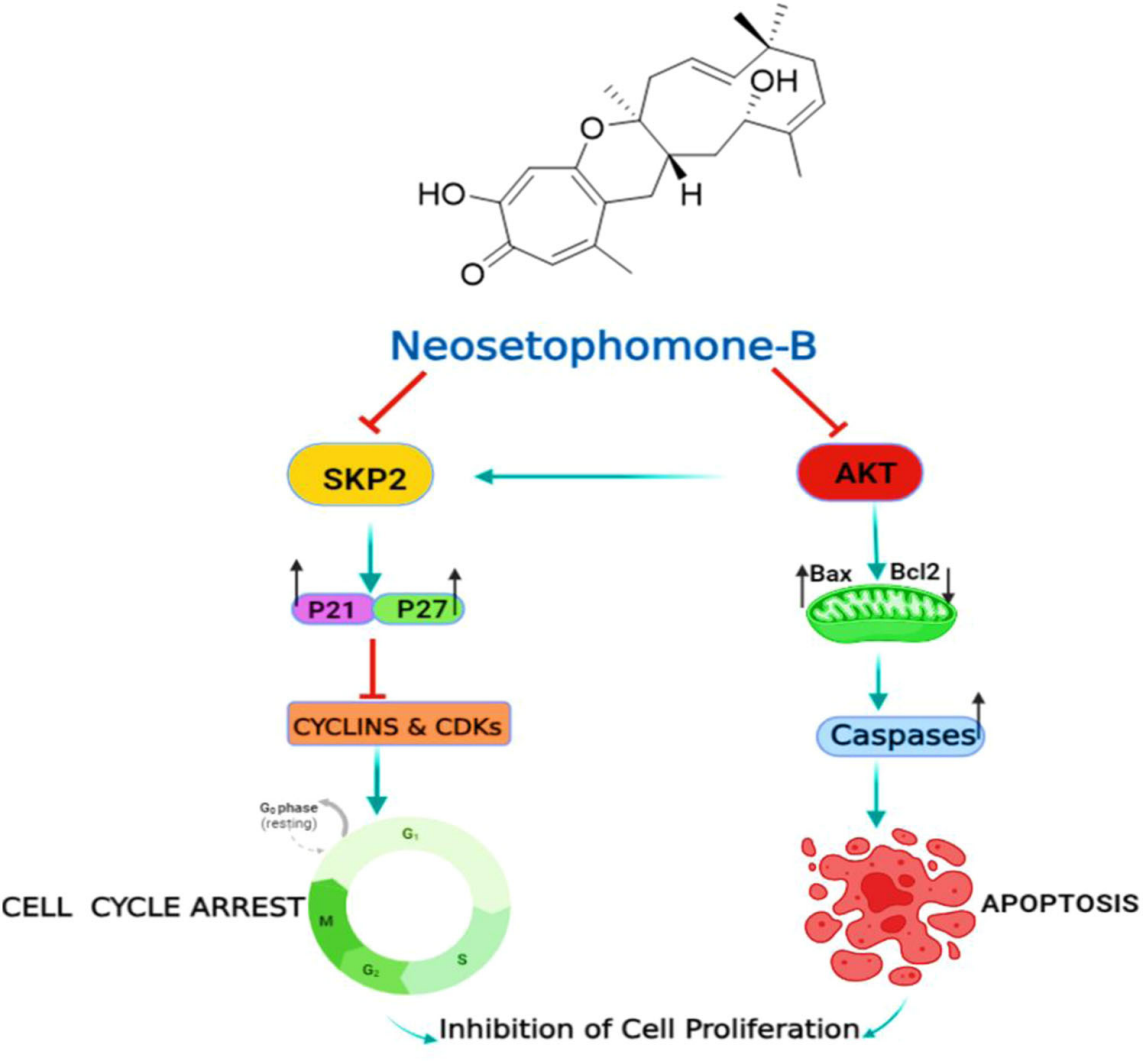
Schematic digram showing possible mechanisms of NSP‐B‐mediated anticancer activity in MM cells. MM, multiple myeloma; NSP‐B, neosetophomone B.

Our findings indicate that NSP‐B effectively inhibits growth and induces apoptosis in MM cell lines (U266 and RPMI8226). NSP‐B treatment reduced the activation of AKT, decreased the IAPs, and modulated the expression of SKP2, p21Kip1, and p27Cip1. Moreover, we observed a higher Bax/BCL2 ratio, loss of MMP, and cytochrome c release into the cytoplasm, thereby initiating caspase‐dependent apoptosis (Figure 6). NSP‐B also enhanced the cytotoxic effects of BTZ in cotreatment. These observations strongly suggest the potential of NSP‐B as a potent anticancer agent against MM.

## AUTHOR CONTRIBUTIONS

**Shilpa Kuttikrishnan**: Data curation; investigation; writing—review and editing. **Fareed Ahmad**: Data curation; formal analysis; methodology. **Jericha M. Mateo**: Data curation; methodology. **Kirti S. Prabhu**: Data curation; writing—review and editing. **Tamam El‐Elimat**: Conceptualization; resources; writing—review and editing. **Nicholas H. Oberlies**: Conceptualization; resources; writing—review and editing. **Cedric J. Pearce**: Resources; writing—review and editing. **Ammira S. Alshabeeb Akil**: Investigation; writing—review and editing. **Ajaz A. Bhat**: Conceptualization; Data curation; investigation; writing—review and editing. **Feras Q. Alali**: Conceptualization; data curation; supervision; writing—original draft. **Shahab Uddin**: Conceptualization; data curation; funding acquisition; investigation; supervision; writing—original draft; writing—review and editing.

## CONFLICT OF INTEREST STATEMENT

The authors declare no conflict of interest.

## Supporting information

Supporting information.

Supporting information.

**Supplementary Figure 1**: NSP‐B mediated apoptosis in MM cells.**A,B)** U266 and RPMI8226 cells were treated with lower doses of NSP‐B (0.1, 0.5,1, 2.5 and 5 µM), followed by staining with fluorescein‐conjugated annexin‐V/PI, and apoptotic cells were determined by flow cytometry. The graph displays the mean ± SD (standard deviation) of three independent experiments. *p < 0.05, **p < 0.01,***p < 0.001.

**Supplementary Figure 2**. NSP‐B mediated phosphorylation of H2AX in MM cells.**A,B)**U266 and RPMI8226 cells were treated with NSP‐B and P‐H2AX were determined by flow cytometry. The graph displays the mean ± SD (standard deviation) of three independent experiments. *p < 0.05, **p < 0.01,***p < 0.001. **C)** U266 and RPMI8226 cells were treated with various doses of NSP‐B, and equal amounts of proteins were immuno‐blotted with antibodies against P‐H2AX and HSP60. Original Western blot images and quantification graphs can be found at Supplementary Files 1 and 2.

**Supplementary Figure 3**: NSP‐B induced caspase and PARP activity in MM cells. **A,B,C and D)** U266 and RPMI8226 cells were treated with NSP‐B(0.1, 0.5,1, 2.5 and 5 µM) for 48 hours and caspase and PARP activity was determined by flow cytometry as described in Materials and Methods section. The graph displays the mean ± SD (standard deviation) of three independent experiments. *p < 0.05, **p < 0.01,***p < 0.001.

**Supplementary Figure 4**: NSP‐B induced caspase and PARP activity in MM cells. U266 and RPMI8226 cells were treated with NSP‐B (0.1, 0.5,1, 2.5 and 5 µM) for 48 hours and equal amounts of proteins were immuno‐blotted with antibodies against Caspase‐3,cleaved caspase‐3. PARP and HSP60. Original Western blot images and quantification graphs can be found at Supplementary Files 1 and 2.

**Supplementary Figure 5**: NSP‐B mediated apoptosis in MM cells.**A,B)** U266 and RPMI8226 cells were treated with and without NSP‐B (0 and 5 µM), followed by staining with fluorescein‐conjugated annexin‐V/PI, and apoptotic cells were determined by flow cytometry. The graph displays the mean ± SD (standard deviation) of three independent experiments. *p < 0.05, **p < 0.01,***p < 0.001. NSP‐B mediated phosphorylation of H2AX in MM cells.**C,D)** U266 and RPMI8226 cells were treated with and without NSP‐B (0 and 5 µM) and P‐H2AX were determined by flow cytometry. The graph displays the mean ± SD (standard deviation) of three independent experiments. *p < 0.05, **p < 0.01,***p < 0.001.

**Supplementary Figure 6**: NSP‐B induced caspase and PARP activity in MM cells. **A,B,C and D)** U266 and RPMI8226 cells were treated with and without NSP‐B(0 and 5 µM) for 48 hours and caspase and PARP activity was determined by flow cytometry as described in Materials and Methods section. The graph displays the mean ± SD (standard deviation) of three independent experiments. *p < 0.05, **p < 0.01,***p < 0.001.

**Supplementary Figure 7**: NSP‐B induced the activation of caspases and downregulated the expression of SKP2.**A)** U266 and RPMI8226 cells were treated with and without NSP‐B (0 and 5 µM) for 48 hours and equal amounts of proteins were immuno‐blotted with antibodies against Caspase‐3,PARP, SKP2,cleaved caspase‐8 and HSP60. Original Western blot images can be found at Supplementary File 2. **B)** The blots were quantified against house keeing gene (HSP60).The graph displays the mean ± SD (standard deviation) of three independent experiments. *p < 0.05, **p < 0.01,***p < 0.001.

## Data Availability

Data sharing is not applicable as no new data are generated, or the article describes entirely theoretical research.

## References

[cbin12101-bib-0001] Akhtar, S. , Achkar, I. W. , Siveen, K. S. , Kuttikrishnan, S. , Prabhu, K. S. , Khan, A. Q. , Ahmed, E. I. , Sahir, F. , Jerobin, J. , Raza, A. , Merhi, M. , Elsabah, H. M. , Taha, R. , Omri, H. E. , Zayed, H. , Dermime, S. , Steinhoff, M. , & Uddin, S. (2019). Sanguinarine induces apoptosis pathway in multiple myeloma cell lines via inhibition of the JaK2/STAT3 signaling. Frontiers in Oncology, 9, 285. 31058086 10.3389/fonc.2019.00285 PMC6478801

[cbin12101-bib-0002] Akhtar, S. , Zarif, L. , Kuttikrishnan, S. , Prabhu, K. S. , Patil, K. , Nisar, S. , Abou‐Saleh, H. , Merhi, M. , Dermime, S. , Bhat, A. A. , & Uddin, S. (2022). Guggulsterone induces apoptosis in multiple myeloma cells by targeting high mobility group box 1 via janus activated kinase/signal transducer and activator of transcription pathway. Cancers, 14(22), 5621. 36428714 10.3390/cancers14225621 PMC9688888

[cbin12101-bib-0003] Al‐Tamimi, M. , Khan, A. Q. , Anver, R. , Ahmad, F. J. , Raza, S. S. , Alam, M. , Buddenkotte, J. , Steinhoff, M. , Uddin, S. , & Mateo, J. M. (2022). Pristimerin mediated anticancer effects and sensitization of human skin cancer cells through modulation of MAPK signaling pathways. Biomedicine & Pharmacotherapy, 156, 113950. 36411635 10.1016/j.biopha.2022.113950

[cbin12101-bib-0004] Bills, G. F. , & Gloer, J. B. (2016). Biologically from the active secondary metabolitesfungi. Microbiology Spectrum, 4(6). 10.1128/microbiolspec.FUNK-0009-2016 27809954

[cbin12101-bib-0005] Boulos, J. C. , Rahama, M. , Hegazy, M. E. F. , & Efferth, T. (2019). Shikonin derivatives for cancer prevention and therapy. Cancer Letters, 459, 248–267. 31132429 10.1016/j.canlet.2019.04.033

[cbin12101-bib-0006] Cai, Z. , Moten, A. , Peng, D. , Hsu, C. C. , Pan, B. S. , Manne, R. , Li, H. , & Lin, H. K. (2020). The Skp2 pathway: A critical target for cancer therapy. Seminars in Cancer Biology, 67(Pt 2), 16–33. 32014608 10.1016/j.semcancer.2020.01.013 PMC9201937

[cbin12101-bib-0007] Cantley, L. C. (2002). The phosphoinositide 3‐kinase pathway. Science, 296(5573), 1655–1657. 12040186 10.1126/science.296.5573.1655

[cbin12101-bib-0008] Cardone, M. H. , Roy, N. , Stennicke, H. R. , Salvesen, G. S. , Franke, T. F. , Stanbridge, E. , Frisch, S. , & Reed, J. C. (1998). Regulation of cell death protease caspase‐9 by phosphorylation. Science, 282(5392), 1318–1321. 9812896 10.1126/science.282.5392.1318

[cbin12101-bib-0009] Chakravarty, D. , Ratnani, P. , Huang, L. , Dovey, Z. , Sobotka, S. , Berryhill, R. , Merisaari, H. , Al Shaarani, M. , Rai, R. , Jambor, I. , Yadav, K. K. , Mittan, S. , Parekh, S. , Kodysh, J. , Wagaskar, V. , Brody, R. , Cordon‐Cardo, C. , Rykunov, D. , Reva, B. , … Tewari, A. K. (2022). Association between incidental pelvic inflammation and aggressive prostate cancer. Cancers, 14(11), 2734. 35681714 10.3390/cancers14112734 PMC9179284

[cbin12101-bib-0010] Chan, C. H. , Lee, S. W. , Li, C. F. , Wang, J. , Yang, W. L. , Wu, C. Y. , Wu, J. , Nakayama, K. I. , Kang, H. Y. , Huang, H. Y. , Hung, M. C. , Pandolfi, P. P. , & Lin, H. K. (2010). Deciphering the transcriptional complex critical for RhoA gene expression and cancer metastasis. Nature Cell Biology, 12(5), 457–467. 20383141 10.1038/ncb2047 PMC3855841

[cbin12101-bib-0011] Chan, C. H. , Li, C. F. , Yang, W. L. , Gao, Y. , Lee, S. W. , Feng, Z. , Huang, H. Y. , Tsai, K. K. C. , Flores, L. G. , Shao, Y. , Hazle, J. D. , Yu, D. , Wei, W. , Sarbassov, D. , Hung, M. C. , Nakayama, K. I. , & Lin, H. K. (2012). The Skp2‐SCF E3 ligase regulates akt ubiquitination, glycolysis, herceptin sensitivity, and tumorigenesis. Cell, 151(4), 913–914. 30360292 10.1016/j.cell.2012.10.025

[cbin12101-bib-0013] Cross, D. A. E. , Alessi, D. R. , Cohen, P. , Andjelkovich, M. , & Hemmings, B. A. (1995). Inhibition of glycogen synthase kinase‐3 by insulin mediated by protein kinase B. Nature, 378(6559), 785–789. 8524413 10.1038/378785a0

[cbin12101-bib-0014] Datta, S. R. , Dudek, H. , Tao, X. , Masters, S. , Fu, H. , Gotoh, Y. , & Greenberg, M. E. (1997). Akt phosphorylation of BAD couples survival signals to the cell‐intrinsic death machinery. Cell, 91(2), 231–241. 9346240 10.1016/s0092-8674(00)80405-5

[cbin12101-bib-0015] Dummler, B. , & Hemmings, B. A. (2007). Physiological roles of PKB/Akt isoforms in development and disease. Biochemical Society Transactions, 35(Pt 2), 231–235. 17371246 10.1042/BST0350231

[cbin12101-bib-0016] Duriez, P. , & Shah, G. M. (1997). Cleavage of poly(ADP‐ribose) polymerase: A sensitive parameter to study cell death. Biochemistry and Cell Biology, 75(4), 337–349. 9493956

[cbin12101-bib-0017] El‐Elimat, T. , Raja, H. A. , Ayers, S. , Kurina, S. J. , Burdette, J. E. , Mattes, Z. , Sabatelle, R. , Bacon, J. W. , Colby, A. H. , Grinstaff, M. W. , Pearce, C. J. , & Oberlies, N. H. (2019). Meroterpenoids from *Neosetophoma* sp.: A dioxa[4.3.3]propellane ring system, potent cytotoxicity, and prolific expression. Organic Letters, 21(2), 529–534. 30620608 10.1021/acs.orglett.8b03769 PMC6343109

[cbin12101-bib-0018] El‐Elimat, T. , Raja, H. A. , Figueroa, M. , Al Sharie, A. H. , Bunch, R. L. , & Oberlies, N. H. (2021). Freshwater fungi as a source of chemical diversity: A review. Journal of Natural Products, 84(3), 898–916. 33662206 10.1021/acs.jnatprod.0c01340 PMC8127292

[cbin12101-bib-0019] Elmore, S. (2007). Apoptosis: A review of programmed cell death. Toxicologic Pathology, 35(4), 495–516. 17562483 10.1080/01926230701320337 PMC2117903

[cbin12101-bib-0020] Firth, J. (2019). Haematology: Multiple myeloma. Clinical Medicine, 19(1), 58–60. 30651246 10.7861/clinmedicine.19-1-58 PMC6399642

[cbin12101-bib-0021] Geyer, F. C. , Li, A. , Papanastasiou, A. D. , Smith, A. , Selenica, P. , Burke, K. A. , Edelweiss, M. , Wen, H. C. , Piscuoglio, S. , Schultheis, A. M. , Martelotto, L. G. , Pareja, F. , Kumar, R. , Brandes, A. , Fan, D. , Basili, T. , Da Cruz Paula, A. , Lozada, J. R. , Blecua, P. , … Reis‐Filho, J. S. (2018). Recurrent hotspot mutations in HRAS Q61 and PI3K‐AKT pathway genes as drivers of breast adenomyoepitheliomas. Nature Communications, 9(1), 1816. 10.1038/s41467-018-04128-5 PMC5940840 29739933

[cbin12101-bib-0022] Goel, H. , Rahul, E. , Gupta, I. , Chopra, A. , Ranjan, A. , Gupta, A. K. , Meena, J. P. , Viswanathan, G. K. , Bakhshi, S. , Misra, A. , Hussain, S. , Kumar, R. , Singh, A. , Rath, G. K. , Sharma, A. , Mittan, S. , & Tanwar, P. (2021). Molecular and genomic landscapes in secondary & therapy related acute myeloid leukemia. American Journal of Blood Research, 11(5), 472–497. 34824881 PMC8610791

[cbin12101-bib-0024] Gstaiger, M. , Jordan, R. , Lim, M. , Catzavelos, C. , Mestan, J. , Slingerland, J. , & Krek, W. (2001). Skp2 is oncogenic and overexpressed in human cancers. Proceedings of the National Academy of Sciences of the United States of America, 98(9), 5043–5048. 11309491 10.1073/pnas.081474898 PMC33160

[cbin12101-bib-0026] Iskandarani, A. , Bhat, A. A. , Siveen, K. S. , Prabhu, K. S. , Kuttikrishnan, S. , Khan, M. A. , Krishnankutty, R. , Kulinski, M. , Nasr, R. R. , Mohammad, R. M. , & Uddin, S. (2016). Bortezomib‐mediated downregulation of S‐phase kinase protein‐2 (SKP2) causes apoptotic cell death in chronic myelogenous leukemia cells. Journal of Translational Medicine, 14, 69. 26956626 10.1186/s12967-016-0823-y PMC4784454

[cbin12101-bib-0028] Kinghorn, A. D. , de Blanco, E. J. C. , Lucas, D. M. , Rakotondraibe, H. L. , Orjala, J. , Soejarto, D. D. , Oberlies, N. H. , Pearce, C. J. , Wani, M. C. , Stockwell, B. R. , Burdette, J. E. , Swanson, S. M. , Fuchs, J. R. , Phelps, M. A. , Xu, L. , Zhang, X. , & Shen, Y. Y. (2016). Discovery of anticancer agents of diverse natural origin. Anticancer Research, 36(11), 5623–5638. 27793884 10.21873/anticanres.11146 PMC5098703

[cbin12101-bib-0029] Kinghorn, A. D. , Chin, Y. W. , & Swanson, S. M. (2009). Discovery of natural product anticancer agents from biodiverse organisms. Current Opinion in Drug Discovery & Development, 12(2), 189–196. 19333864 PMC2877274

[cbin12101-bib-0030] Kulinski, M. , Achkar, I. W. , Haris, M. , Dermime, S. , Mohammad, R. M. , & Uddin, S. (2018). Dysregulated expression of SKP2 and its role in hematological malignancies. Leukemia & Lymphoma, 59(5), 1051–1063. 28797197 10.1080/10428194.2017.1359740

[cbin12101-bib-0031] Kuttikrishnan, S. , Bhat, A. A. , Mateo, J. M. , Ahmad, F. , Alali, F. Q. , El‐Elimat, T. , Oberlies, N. H. , Pearce, C. J. , & Uddin, S. (2022). Anticancer activity of neosetophomone B by targeting AKT/SKP2/MTH1 axis in leukemic cells. Biochemical and Biophysical Research Communications, 601, 59–64. 35228122 10.1016/j.bbrc.2022.02.071

[cbin12101-bib-0032] Kuttikrishnan, S. , Masoodi, T. , Sher, G. , Bhat, A. A. , Patil, K. , El‐Elimat, T. , Oberlies, N. H. , Pearce, C. J. , Haris, M. , Ahmad, A. , Alali, F. Q. , & Uddin, S. (2022). Bioinformatics analysis reveals FOXM1/BUB1B signaling pathway as a key target of neosetophomone B in human leukemic cells: A gene network‐based microarray analysis. Frontiers in Oncology, 12, 929996. 35847923 10.3389/fonc.2022.929996 PMC9283897

[cbin12101-bib-0033] Kuttikrishnan, S. , Prabhu, K. S. , Al Sharie, A. H. , Al Zu'bi, Y. O. , Alali, F. Q. , Oberlies, N. H. , Ahmad, A. , El‐Elimat, T. , & Uddin, S. (2022). Natural resorcylic acid lactones: A chemical biology approach for anticancer activity. Drug Discovery Today, 27(2), 547–557. 34655796 10.1016/j.drudis.2021.10.001

[cbin12101-bib-0034] Kuttikrishnan, S. , Siveen, K. S. , Prabhu, K. S. , Khan, A. Q. , Ahmed, E. I. , Akhtar, S. , Ali, T. A. , Merhi, M. , Dermime, S. , Steinhoff, M. , & Uddin, S. (2019). Curcumin induces apoptotic cell death via inhibition of PI3‐kinase/AKT pathway in B‐precursor acute lymphoblastic leukemia. Frontiers in Oncology, 9, 484. 31275848 10.3389/fonc.2019.00484 PMC6593070

[cbin12101-bib-0035] Li, C. , Cao, W. , Que, Y. , Wang, Q. , Xiao, Y. , Gu, C. , Wang, D. , Wang, J. , Jiang, L. , Xu, H. , Xu, J. , Zhou, X. , Hong, Z. , Wang, N. , Huang, L. , Zhang, S. , Chen, L. , Mao, X. , Xiao, M. , … Zhou, J. (2021). A phase I study of anti‐BCMA CAR T cell therapy in relapsed/refractory multiple myeloma and plasma cell leukemia. Clinical and Translational Medicine, 11(3), e346. 33784005 10.1002/ctm2.346 PMC7943908

[cbin12101-bib-0036] Li, Z. , Wang, C. , Prendergast, G. , & Pestell, R. G. (2006). Cyclin D1 functions in cell migration. Cell Cycle, 5(21), 2440–2442. 17106256 10.4161/cc.5.21.3428

[cbin12101-bib-0037] Lin, H. K. , Chen, Z. , Wang, G. , Nardella, C. , Lee, S. W. , Chan, C. H. , Yang, W. L. , Wang, J. , Egia, A. , Nakayama, K. I. , Cordon‐Cardo, C. , Teruya‐Feldstein, J. , & Pandolfi, P. P. (2010). Skp2 targeting suppresses tumorigenesis by Arf‐p53‐independent cellular senescence. Nature, 464(7287), 374–379. 20237562 10.1038/nature08815 PMC2928066

[cbin12101-bib-0040] Liu, R. , Gao, Q. , Foltz, S. M. , Fowles, J. S. , Yao, L. , Wang, J. T. , Cao, S. , Sun, H. , Wendl, M. C. , Sethuraman, S. , Weerasinghe, A. , Rettig, M. P. , Storrs, E. P. , Yoon, C. J. , Wyczalkowski, M. A. , McMichael, J. F. , Kohnen, D. R. , King, J. , Goldsmith, S. R. , … Ding, L. (2021). Co‐evolution of tumor and immune cells during progression of multiple myeloma. Nature Communications, 12(1), 2559. 10.1038/s41467-021-22804-x PMC8105337 33963182

[cbin12101-bib-0041] Manning, B. D. , & Cantley, L. C. (2007). AKT/PKB signaling: Navigating downstream. Cell, 129(7), 1261–1274. 17604717 10.1016/j.cell.2007.06.009 PMC2756685

[cbin12101-bib-0042] Mondal, A. , Gandhi, A. , Fimognari, C. , Atanasov, A. G. , & Bishayee, A. (2019). Alkaloids for cancer prevention and therapy: Current progress and future perspectives. European Journal of Pharmacology, 858, 172472. 31228447 10.1016/j.ejphar.2019.172472

[cbin12101-bib-0043] Murray, M. Y. , Auger, M. J. , & Bowles, K. M. (2014). Overcoming bortezomib resistance in multiple myeloma. Biochemical Society Transactions, 42(4), 804–808. 25109961 10.1042/BST20140126

[cbin12101-bib-0044] Oben, K. Z. , Gachuki, B. W. , Alhakeem, S. S. , McKenna, M. K. , Liang, Y. , St Clair, D. K. , Rangnekar, V. M. , & Bondada, S. (2017). Radiation induced apoptosis of murine bone marrow cells is independent of early growth response 1 (EGR1). PLoS One, 12(1), e0169767. 28081176 10.1371/journal.pone.0169767 PMC5230770

[cbin12101-bib-0045] Prabhu, K. , Siveen, K. , Kuttikrishnan, S. , Jochebeth, A. , Ali, T. , Elareer, N. , Iskandarani, A. , Quaiyoom Khan, A. , Merhi, M. , Dermime, S. , El‐Elimat, T. , Oberlies, N. , Alali, F. , Steinhoff, M. , & Uddin, S. (2019). Greensporone A, a fungal secondary metabolite suppressed constitutively activated AKT via ROS generation and induced apoptosis in leukemic cell lines. Biomolecules, 9(4), 126. 30934922 10.3390/biom9040126 PMC6523683

[cbin12101-bib-0046] Prabhu, K. S. , Bhat, A. A. , Siveen, K. S. , Kuttikrishnan, S. , Raza, S. S. , Raheed, T. , Jochebeth, A. , Khan, A. Q. , Chawdhery, M. Z. , Haris, M. , Kulinski, M. , Dermime, S. , Steinhoff, M. , & Uddin, S. (2021). Sanguinarine mediated apoptosis in non‐small cell lung cancer via generation of reactive oxygen species and suppression of JAK/STAT pathway. Biomedicine & Pharmacotherapy, 144, 112358. 34794241 10.1016/j.biopha.2021.112358

[cbin12101-bib-0047] Prabhu, K. S. , Siveen, K. S. , Kuttikrishnan, S. , Iskandarani, A. , Tsakou, M. , Achkar, I. W. , Therachiyil, L. , Krishnankutty, R. , Parray, A. , Kulinski, M. , Merhi, M. , Dermime, S. , Mohammad, R. M. , & Uddin, S. (2017). Targeting of X‐linked inhibitor of apoptosis protein and PI3‐kinase/AKT signaling by embelin suppresses growth of leukemic cells. PLoS One, 12(7), e0180895. 28704451 10.1371/journal.pone.0180895 PMC5509148

[cbin12101-bib-0048] Radke, S. , Pirkmaier, A. , & Germain, D. (2005). Differential expression of the F‐box proteins Skp2 and Skp2B in breast cancer. Oncogene, 24(21), 3448–3458. 15782142 10.1038/sj.onc.1208328

[cbin12101-bib-0049] Ramisetty, S. , Kulkarni, P. , Bhattacharya, S. , Nam, A. , Singhal, S. S. , Guo, L. , Mirzapoiazova, T. , Mambetsariev, B. , Mittan, S. , Malhotra, J. , Pisick, E. , Subbiah, S. , Rajurkar, S. , Massarelli, E. , Salgia, R. , & Mohanty, A. (2023). A systems biology approach for addressing cisplatin resistance in non‐small cell lung cancer. Journal of Clinical Medicine, 12(2), 599. 36675528 10.3390/jcm12020599 PMC9861808

[cbin12101-bib-0050] Scuoppo, C. , Wang, J. , Persaud, M. , Mittan, S. K. , Basso, K. , Pasqualucci, L. , Rabadan, R. , Inghirami, G. , Grandori, C. , Bosch, F. , & Dalla‐Favera, R. (2019). Repurposing dasatinib for diffuse large B cell lymphoma. Proceedings of the National Academy of Sciences of the United States of America, 116(34), 16981–16986. 31383760 10.1073/pnas.1905239116 PMC6708382

[cbin12101-bib-0051] Shapiro, G. I. (2006). Cyclin‐dependent kinase pathways as targets for cancer treatment. Journal of Clinical Oncology, 24(11), 1770–1783. 16603719 10.1200/JCO.2005.03.7689

[cbin12101-bib-0052] Shishodia, S. , Sethi, G. , Ahn, K. S. , & Aggarwal, B. B. (2007). Guggulsterone inhibits tumor cell proliferation, induces S‐phase arrest, and promotes apoptosis through activation of c‐Jun N‐terminal kinase, suppression of Akt pathway, and downregulation of antiapoptotic gene products. Biochemical Pharmacology, 74(1), 118–130. 17475222 10.1016/j.bcp.2007.03.026 PMC2744036

[cbin12101-bib-0053] Tang, F. , Wang, Y. , Hemmings, B. A. , Rüegg, C. , & Xue, G. (2018). PKB/Akt‐dependent regulation of inflammation in cancer. Seminars in Cancer Biology, 48, 62–69. 28476657 10.1016/j.semcancer.2017.04.018

[cbin12101-bib-0055] Thorsteinsdottir, S. , Dickman, P. W. , Landgren, O. , Blimark, C. , Hultcrantz, M. , Turesson, I. , Björkholm, M. , & Kristinsson, S. Y. (2018). Dramatically improved survival in multiple myeloma patients in the recent decade: Results from a Swedish population‐based study. Haematologica, 103(9), e412–e415. 29567776 10.3324/haematol.2017.183475 PMC6119139

[cbin12101-bib-0056] Tsvetkov, L. M. , Yeh, K. H. , Lee, S. J. , Sun, H. , & Zhang, H. (1999). p27(Kip1) ubiquitination and degradation is regulated by the SCF(Skp2) complex through phosphorylated Thr187 in p27. Current Biology, 9(12), 661–664. 10375532 10.1016/s0960-9822(99)80290-5

[cbin12101-bib-0057] Uddin, S. , Bhat, A. A. , Krishnankutty, R. , Mir, F. , Kulinski, M. , & Mohammad, R. M. (2016). Involvement of F‐BOX proteins in progression and development of human malignancies. Seminars in Cancer Biology, 36, 18–32. 26410033 10.1016/j.semcancer.2015.09.008

[cbin12101-bib-1057] Uddin, S. , Siraj, A. K. , Al‐Rasheed, M. , Ahmed, M. , Bu, R. , Myers, J. N. R. , & Al‐Kuraya, K. S. (2008). Fatty acid synthase and AKT pathway signaling in a subset of papillary thyroid cancers. The Journal of Clinical Endocrinology & Metabolism, 93(10), 4088–4097. 10.1210/jc.2008-0503 18682509

[cbin12101-bib-0058] Vasilikos, L. , Spilgies, L. M. , Knop, J. , & Wong, W. W. L. (2017). Regulating the balance between necroptosis, apoptosis and inflammation by inhibitors of apoptosis proteins. Immunology & Cell Biology, 95(2), 160–165. 27904150 10.1038/icb.2016.118

[cbin12101-bib-0059] Vermeulen, K. , Van Bockstaele, D. R. , & Berneman, Z. N. (2003). The cell cycle: A review of regulation, deregulation and therapeutic targets in cancer. Cell Proliferation, 36(3), 131–149. 12814430 10.1046/j.1365-2184.2003.00266.x PMC6496723

[cbin12101-bib-0060] Wang, J. , Sato, K. , O'Donnell, E. , Singla, A. , Yaguare, S. , Aldahamsheh, O. , Batko, B. , Borjihan, H. , Tingling, J. , Zhang, J. , Weiser, D. A. , Loeb, D. M. , Gorlick, R. , Schwartz, E. L. , Yang, R. , Zi, X. , Zhao, H. , Geller, D. S. , & Hoang, B. H. (2020). Skp2 depletion reduces tumor‐initiating properties and promotes apoptosis in synovial sarcoma. Translational Oncology, 13(10), 100809. 32623326 10.1016/j.tranon.2020.100809 PMC7334610

[cbin12101-bib-0062] Wei, W. , Ayad, N. G. , Wan, Y. , Zhang, G. J. , Kirschner, M. W. , & Kaelin, Jr., W. G. (2004). Degradation of the SCF component Skp2 in cell‐cycle phase G1 by the anaphase‐promoting complex. Nature, 428(6979), 194–198. 15014503 10.1038/nature02381

[cbin12101-bib-0064] Yang, W. C. , & Lin, S. F. (2015). Mechanisms of drug resistance in relapse and refractory multiple myeloma. BioMed Research International, 2015, 1–17. 10.1155/2015/341430 PMC4663284 26649299

[cbin12101-bib-0065] Yuan, R. , Hou, Y. , Sun, W. , Yu, J. , Liu, X. , Niu, Y. , Lu, J. J. , & Chen, X. (2017). Natural products to prevent drug resistance in cancer chemotherapy: A review. Annals of the New York Academy of Sciences, 1401(1), 19–27. 28891091 10.1111/nyas.13387

[cbin12101-bib-0066] Zaal, E. A. , Wu, W. , Jansen, G. , Zweegman, S. , Cloos, J. , & Berkers, C. R. (2017). Bortezomib resistance in multiple myeloma is associated with increased serine synthesis. Cancer & Metabolism, 5, 7. 28855983 10.1186/s40170-017-0169-9 PMC5575874

[cbin12101-bib-0067] Zhao, M. , Tang, Y. , Xie, J. , Zhao, Z. , & Cui, H. (2021). Meroterpenoids produced by fungi: Occurrence, structural diversity, biological activities, and their molecular targets. European Journal of Medicinal Chemistry, 209, 112860. 33032085 10.1016/j.ejmech.2020.112860

