# Supplementary material for: Neosetophomone B induces apoptosis in multiple myeloma cells via targeting of AKT/SKP2 signaling pathway
Source: Cell Biol Int. 2023 Oct 26;48(2):190–200. doi: 10.1002/cbin.12101 (PMC10952688; doi:10.1002/cbin.12101)

Figure 1 E

U266

RPMI8226

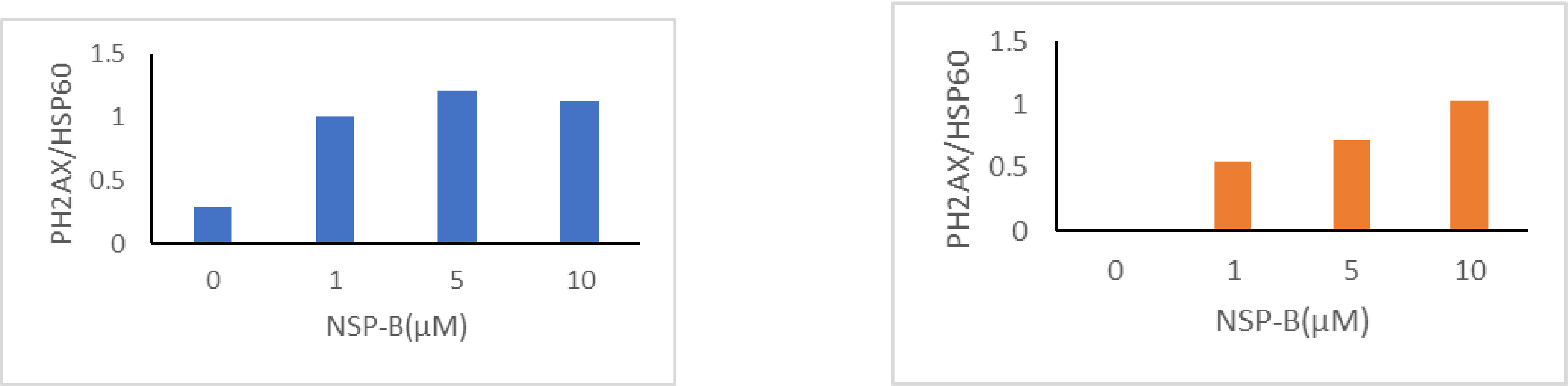

Figure 1 B

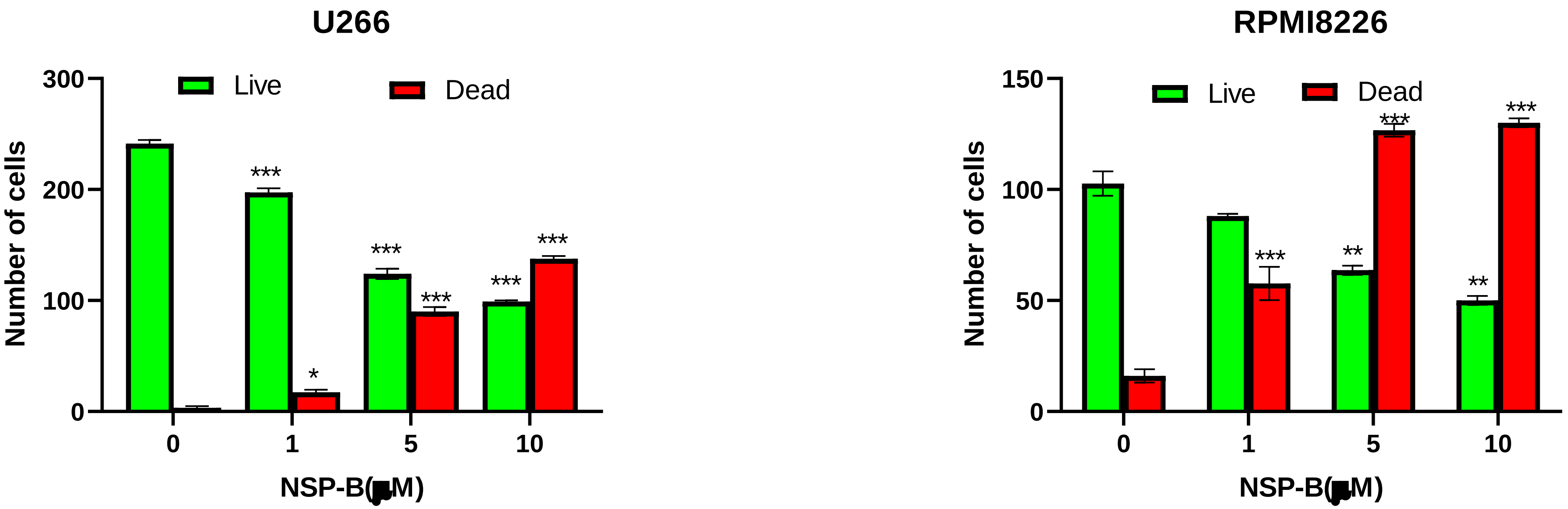

U266
Live
Dead
300
200
100
0
Number of cells
***
*
***
***
***
***
0
1
5
10
NSP-B(μM)
RPMI8226
Live
Dead
150
100
50
0
Number of cells
***
***
***
**
**
0
1
5
10
NSP-B(μM)

Figure 2A

U266

RPMI8226

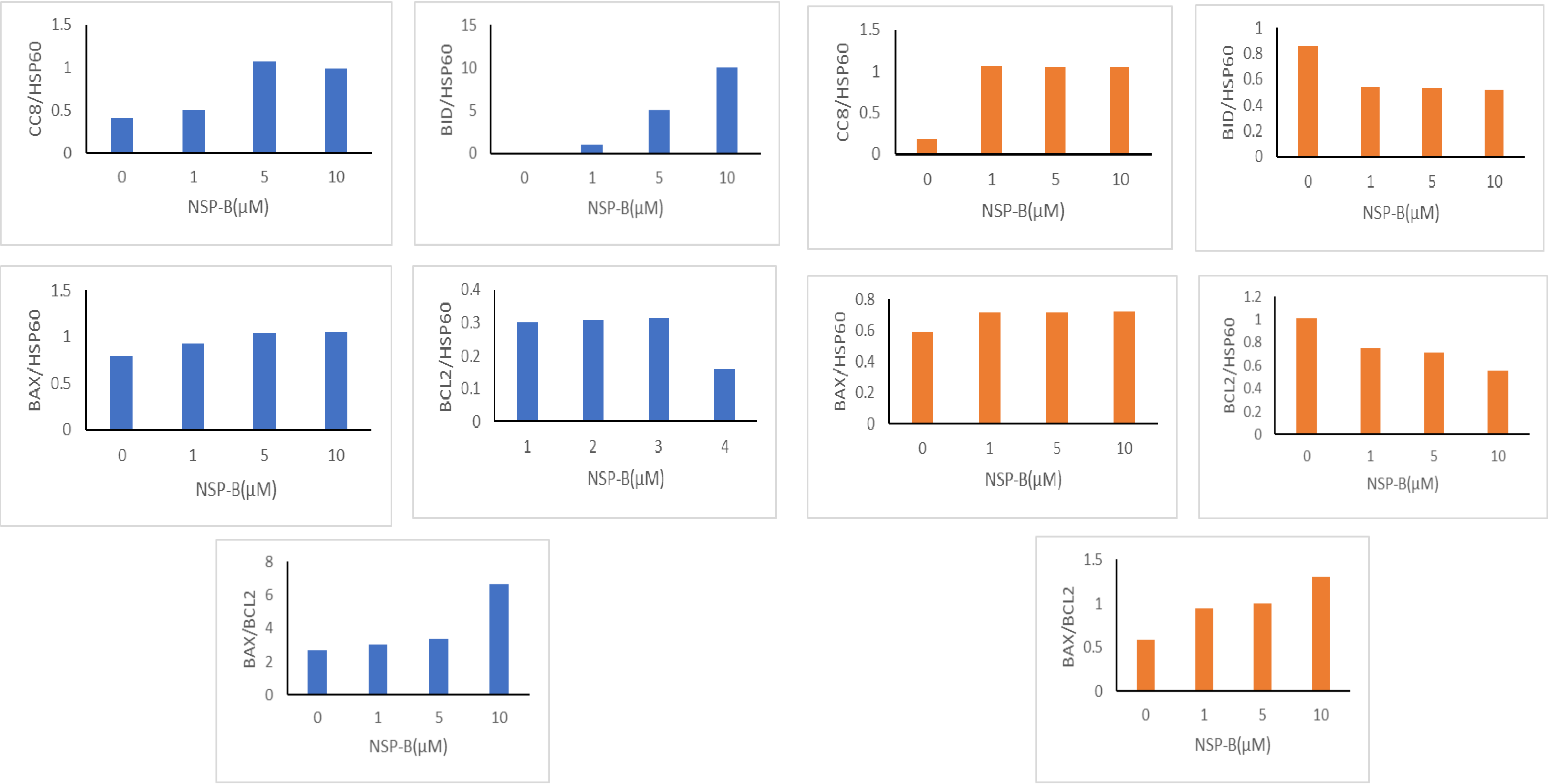

Figure 2C

U266

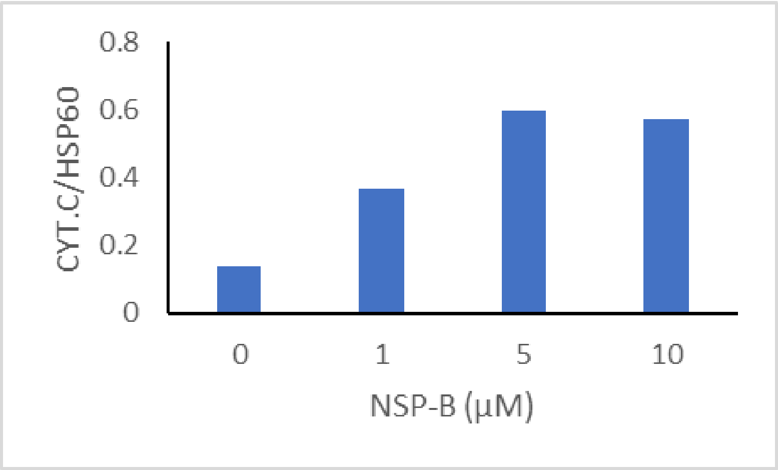

RPMI8226

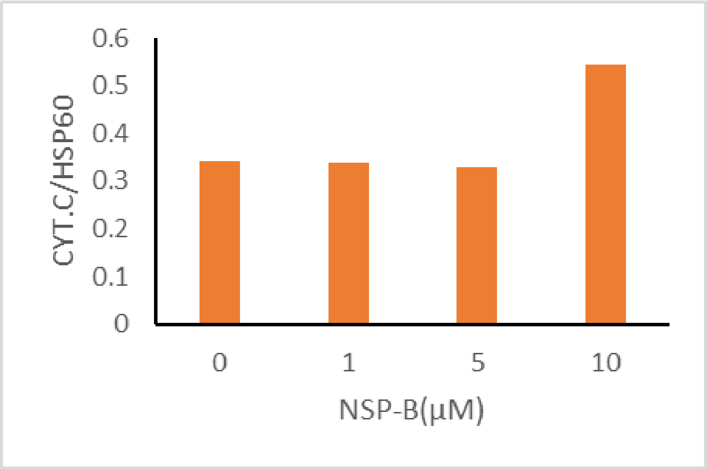

Figure 2 D

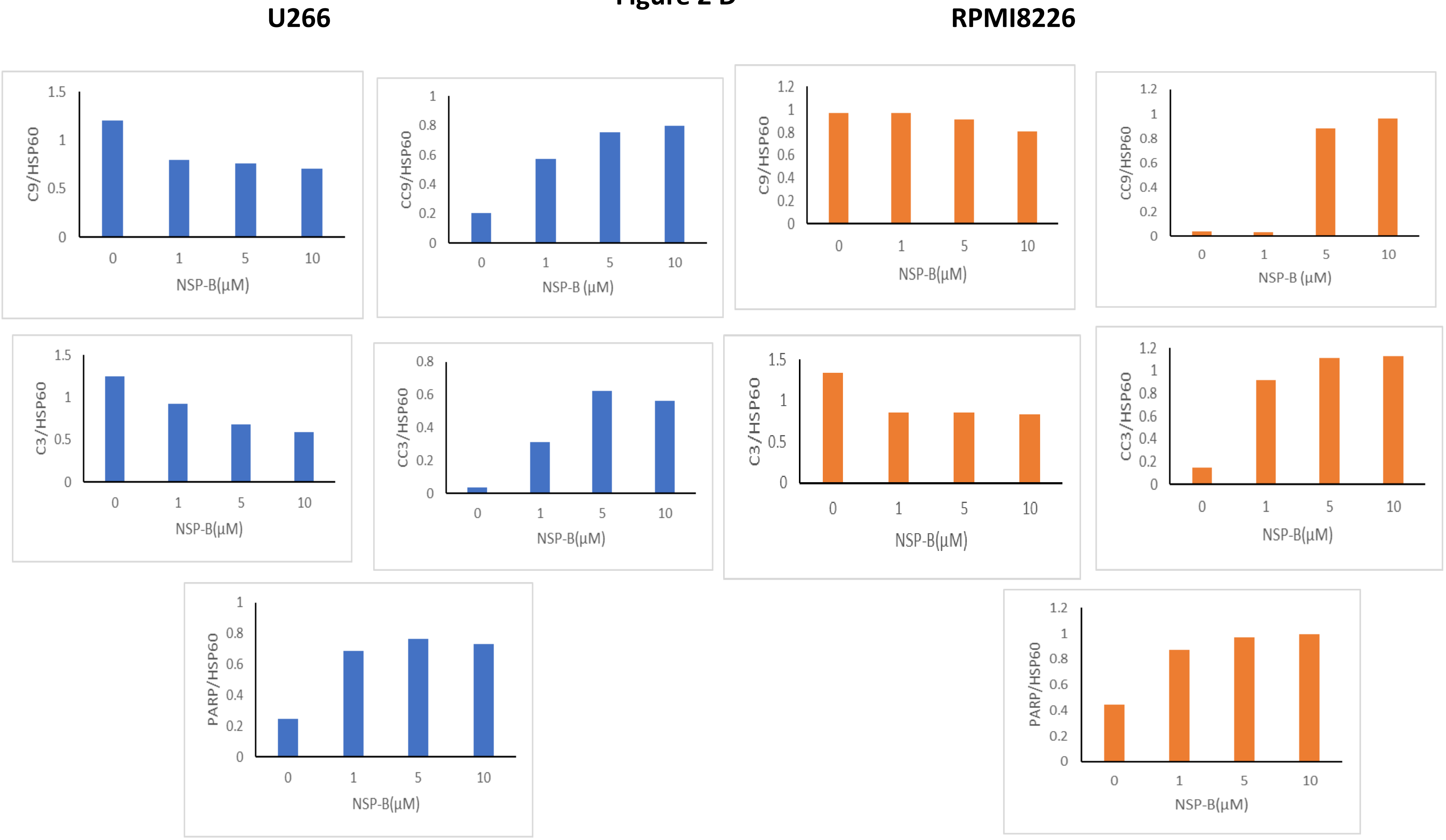
U266
RPMI8226
C9/HSP60
CC9/HSP60
C3/HSP60
CC3/HSP60
PARP/HSP60
NSP-B(μM)
NSP-B (μM)
0
1
5
10

Figure 2 E

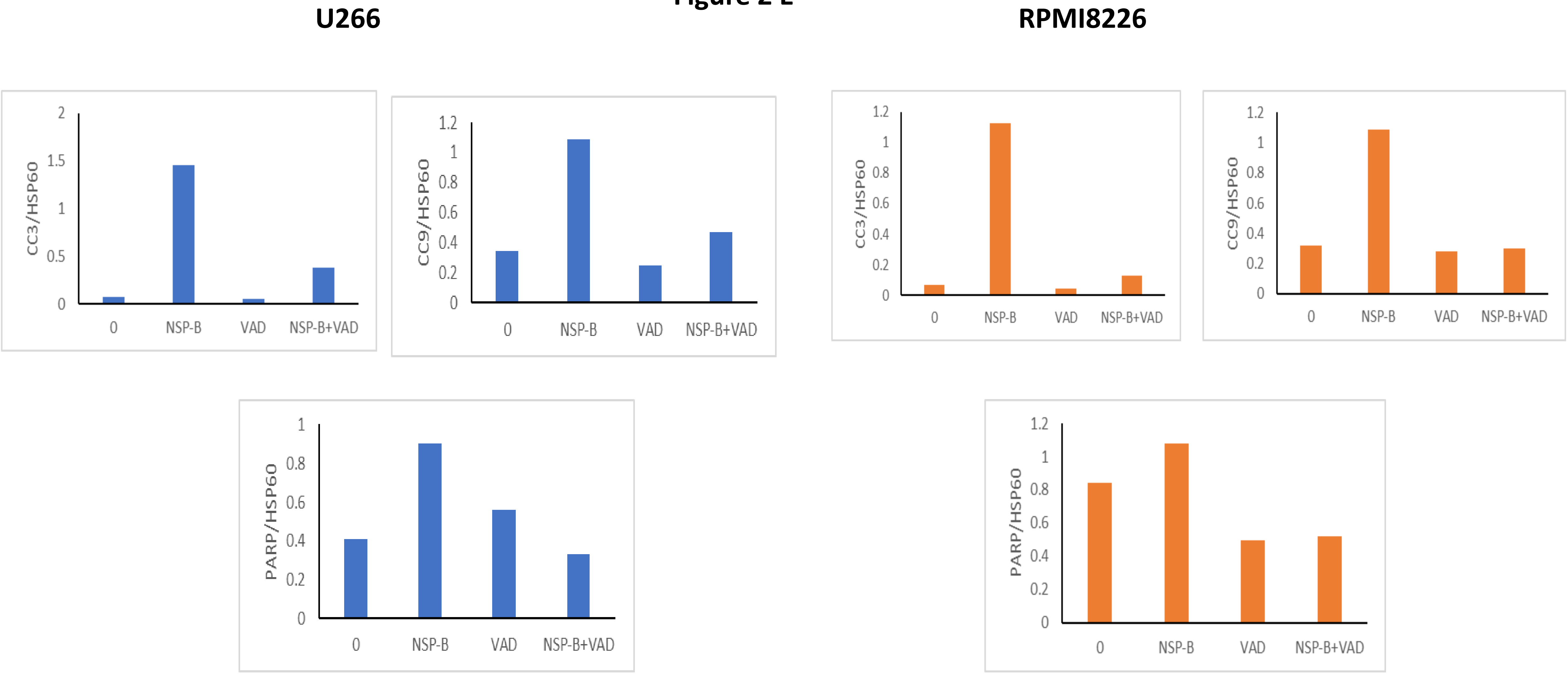
U266
RPMI8226
CC3/HSP60
CC9/HSP60
PARP/HSP60
0
NSP-B
VAD
NSP-B+VAD
0
0.2
0.4
0.5
0.6
0.8
1
1.2
1.5
2

Figure 3 A

U266 **RPMI8226**

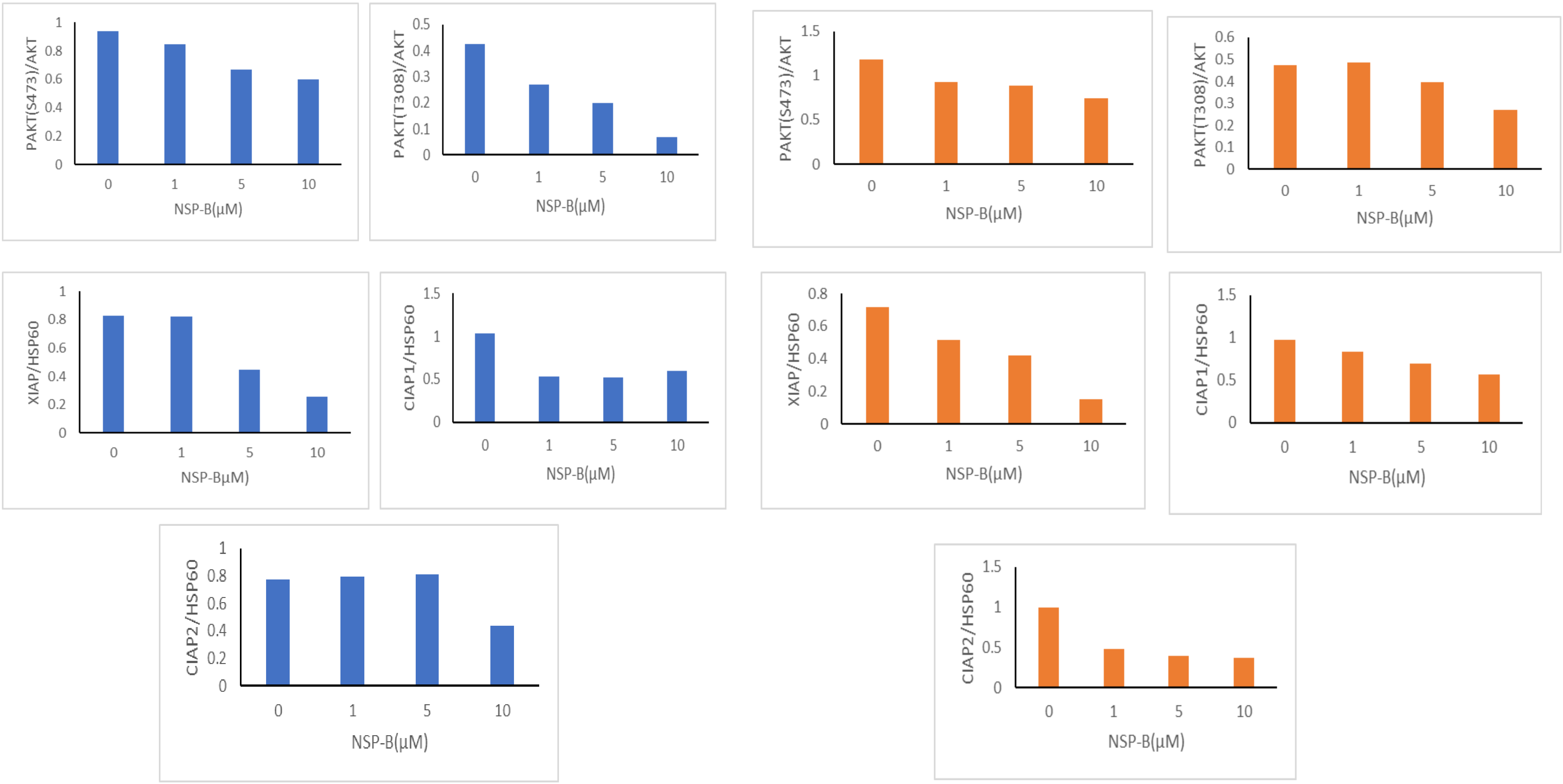

Figure 3 B

U266 **RPMI8226**

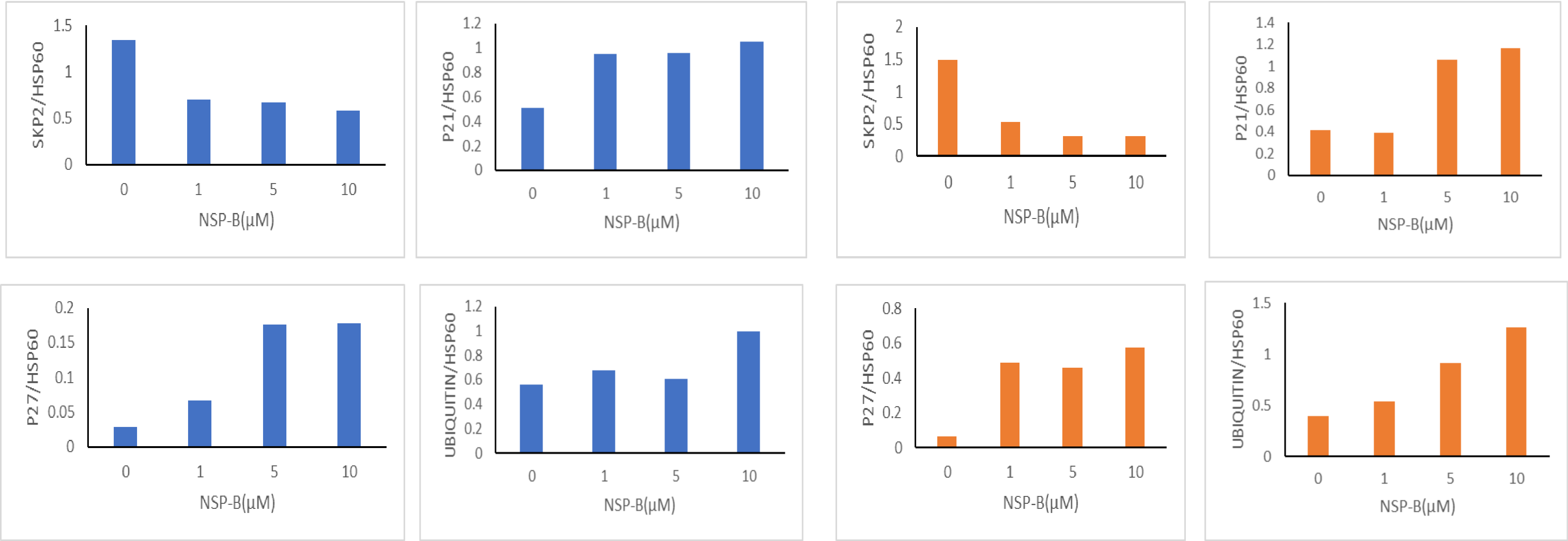

Figure 3 C

U266

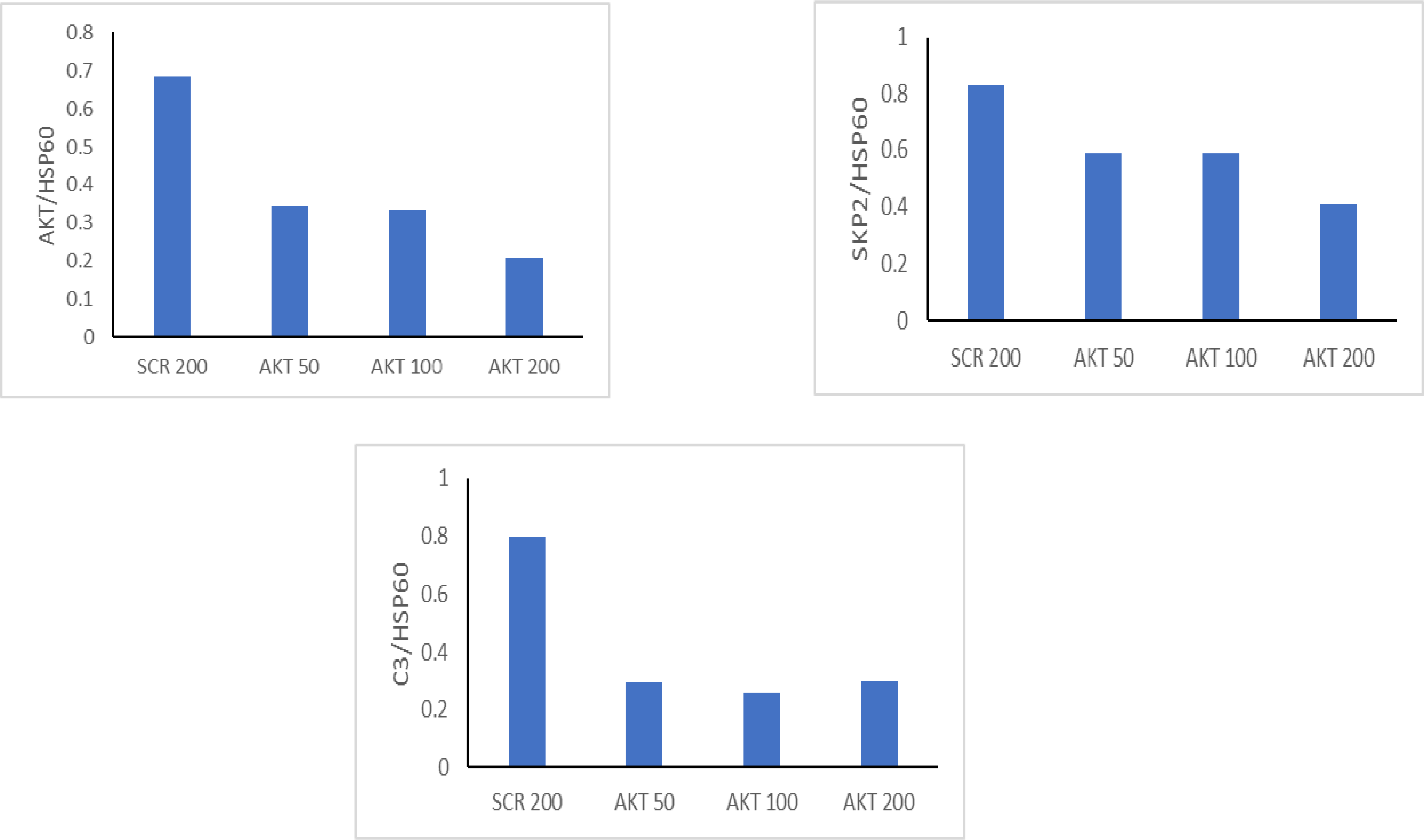

Figure 4

U266 **RPMI8226**

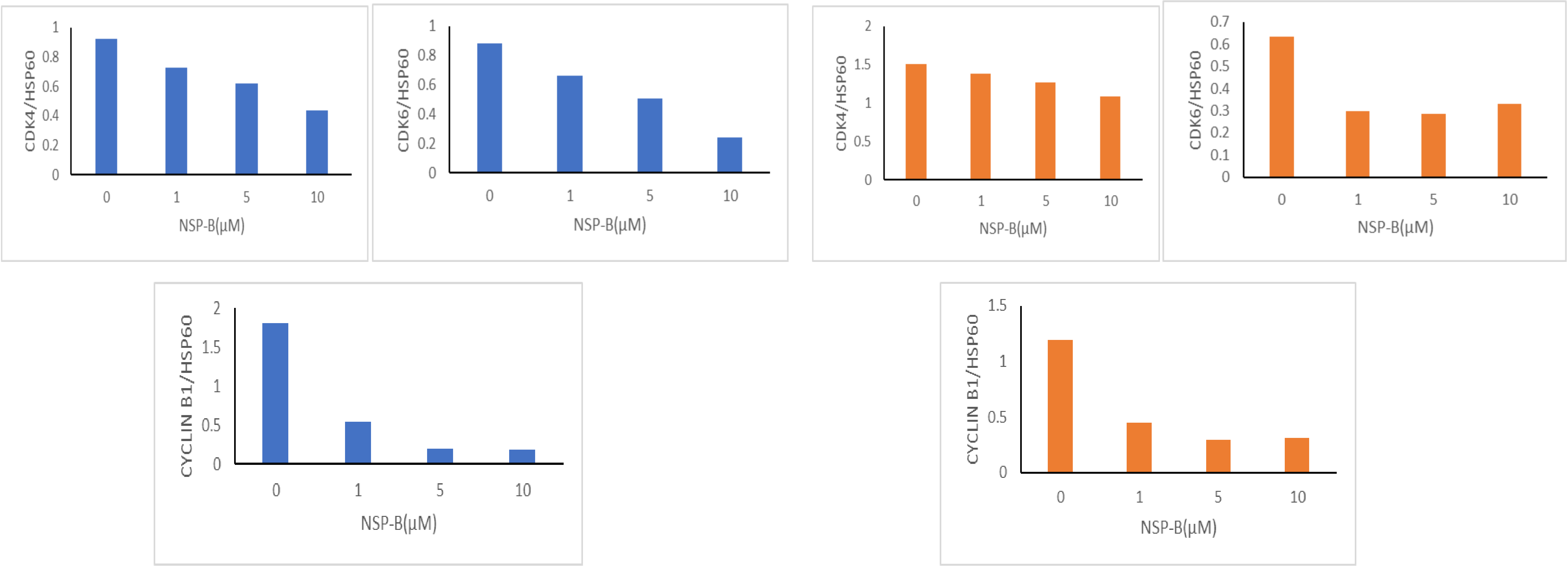

Figure 5A

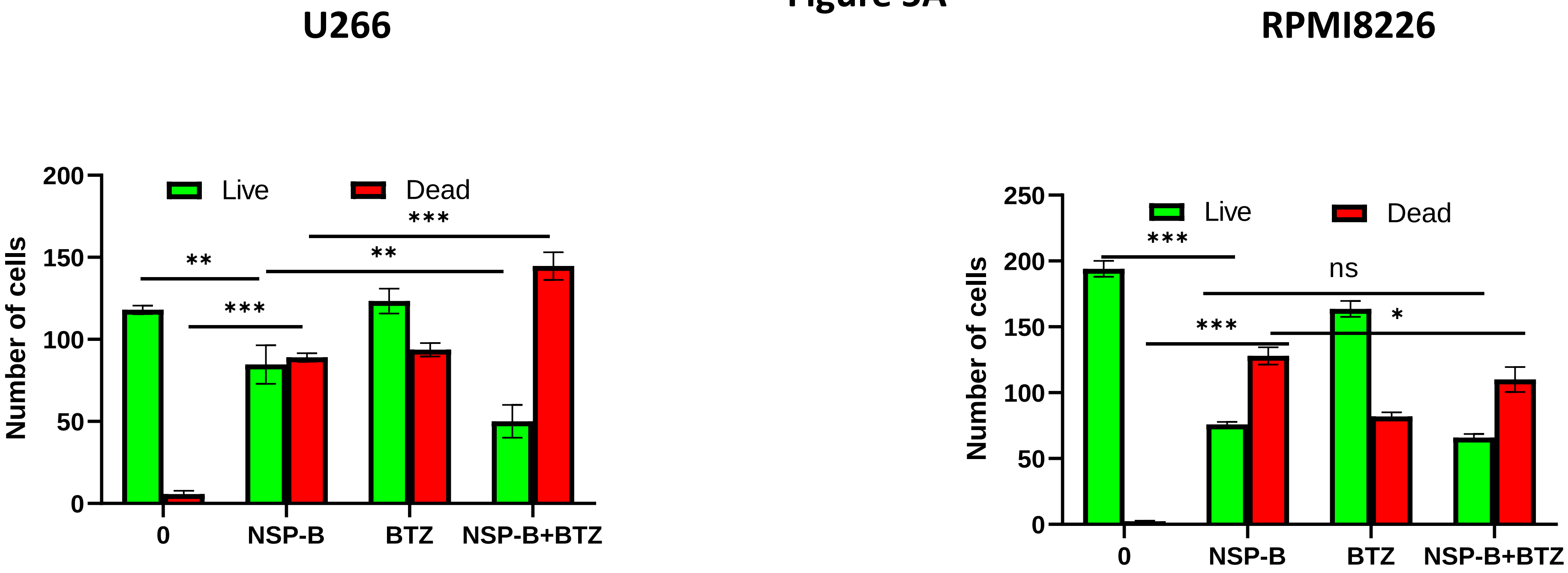
U266
RPMI8226
Live
Dead
Number of cells
200
150
100
50
0
250
**
***
ns
*
0
NSP-B
BTZ
NSP-B+BTZ

Figure 5B

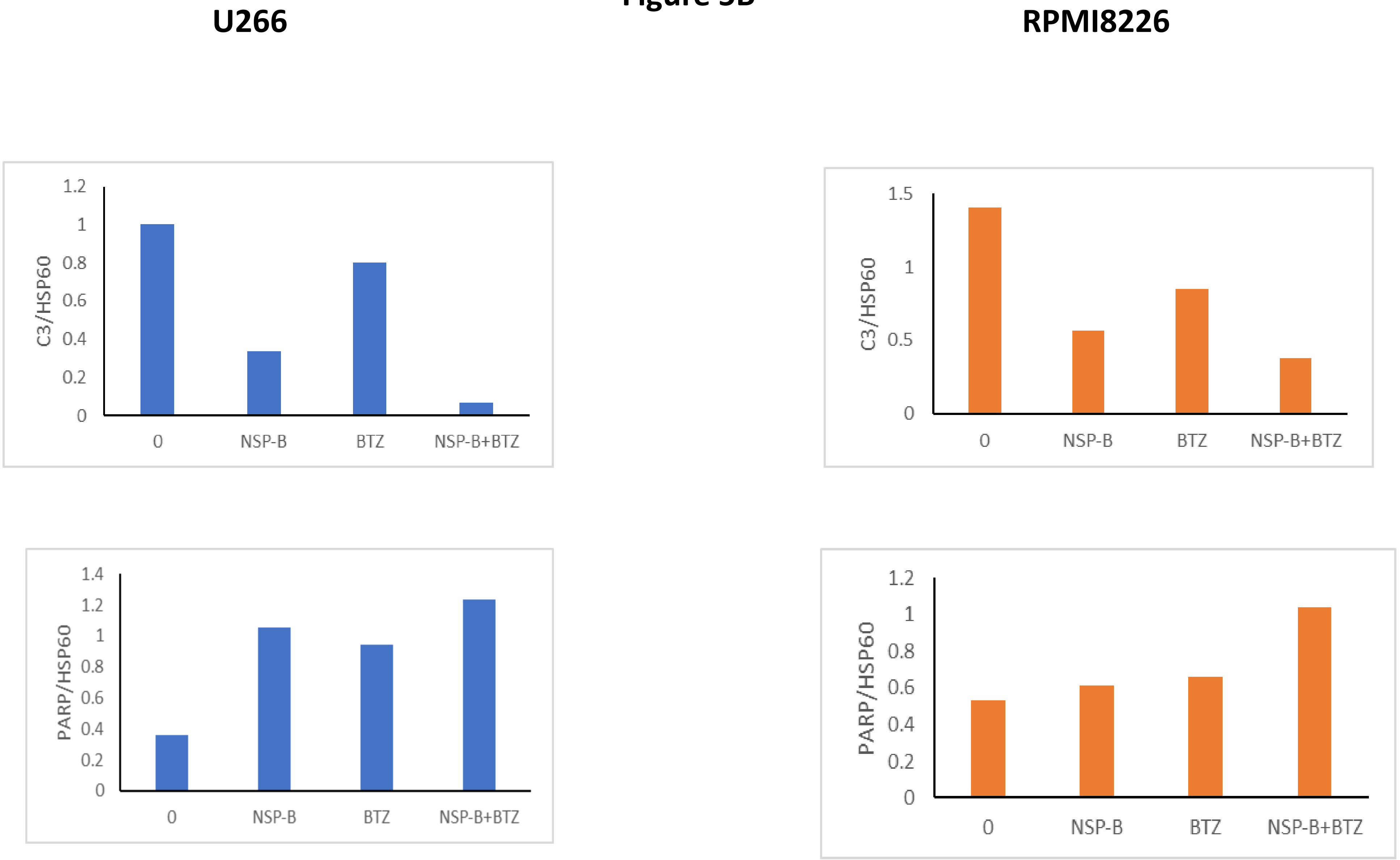

U266
RPMI8226
C3/HSP60
PARP/HSP60
0
NSP-B
BTZ
NSP-B+BTZ
0
0.2
0.4
0.6
0.8
1
1.2
1.4
0.5
1.5

Supplementary Figure 2C

U266

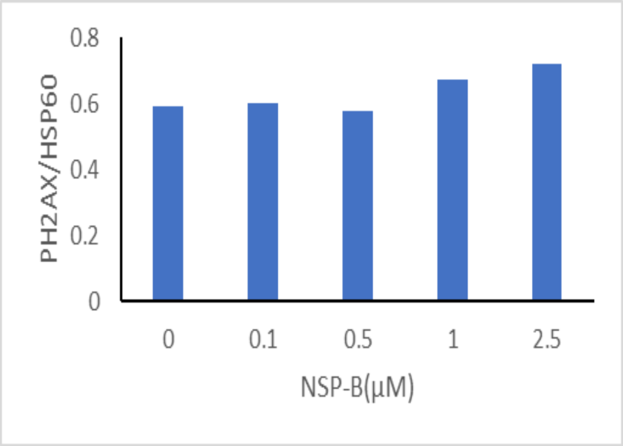

RPMI8226

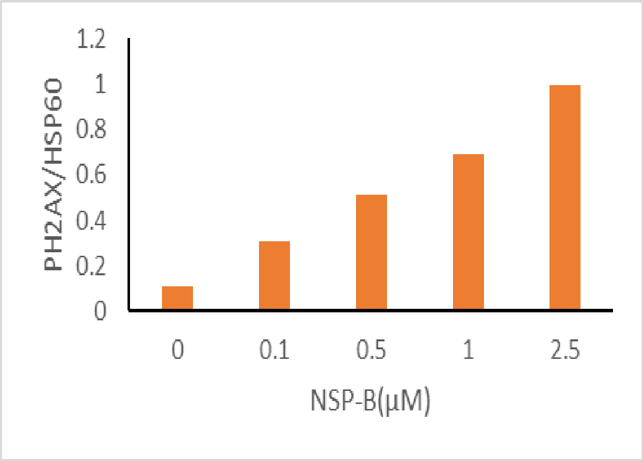

U266 **Supplementary Figure 4** **RPMI8226**

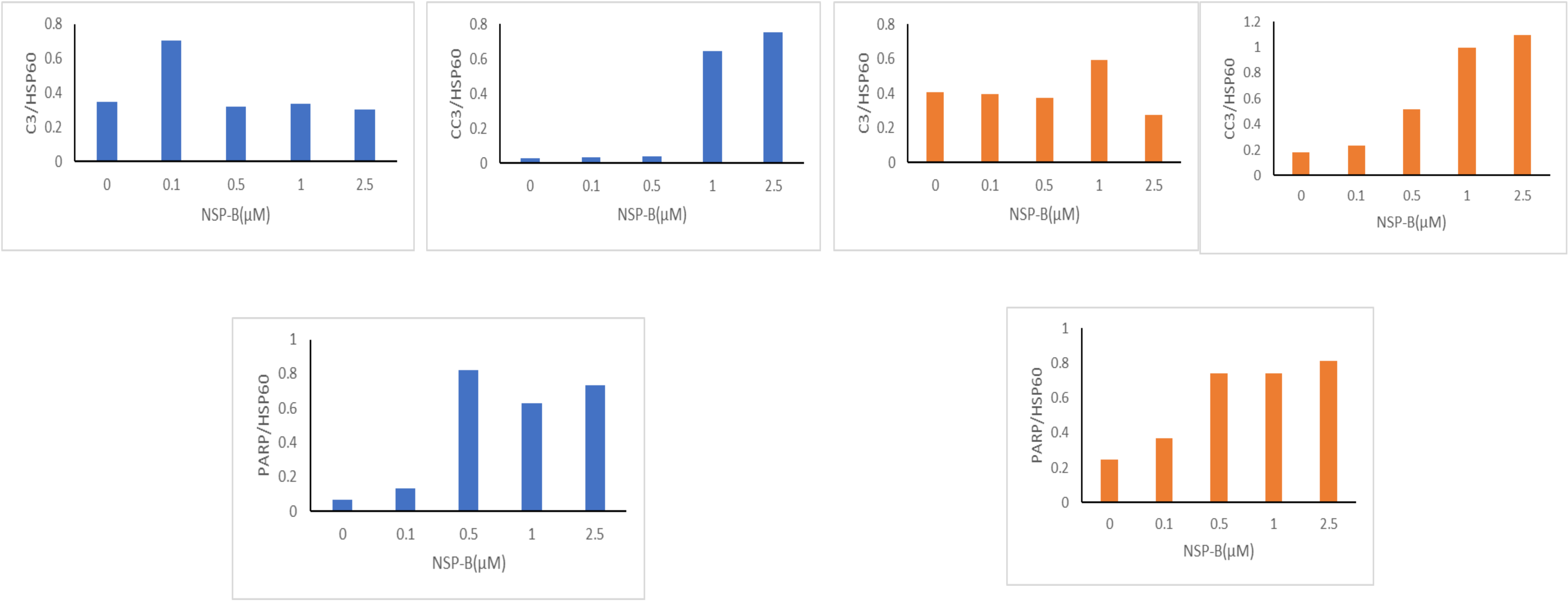

Supplement: Supplementary file 1 — Supporting information. [file CBIN-48-190-s003.pdf]
