# Supplementary material for: Neosetophomone B induces apoptosis in multiple myeloma cells via targeting of AKT/SKP2 signaling pathway
Source: Cell Biol Int. 2023 Oct 26;48(2):190–200. doi: 10.1002/cbin.12101 (PMC10952688; doi:10.1002/cbin.12101)

Figure 1

Figure 1B

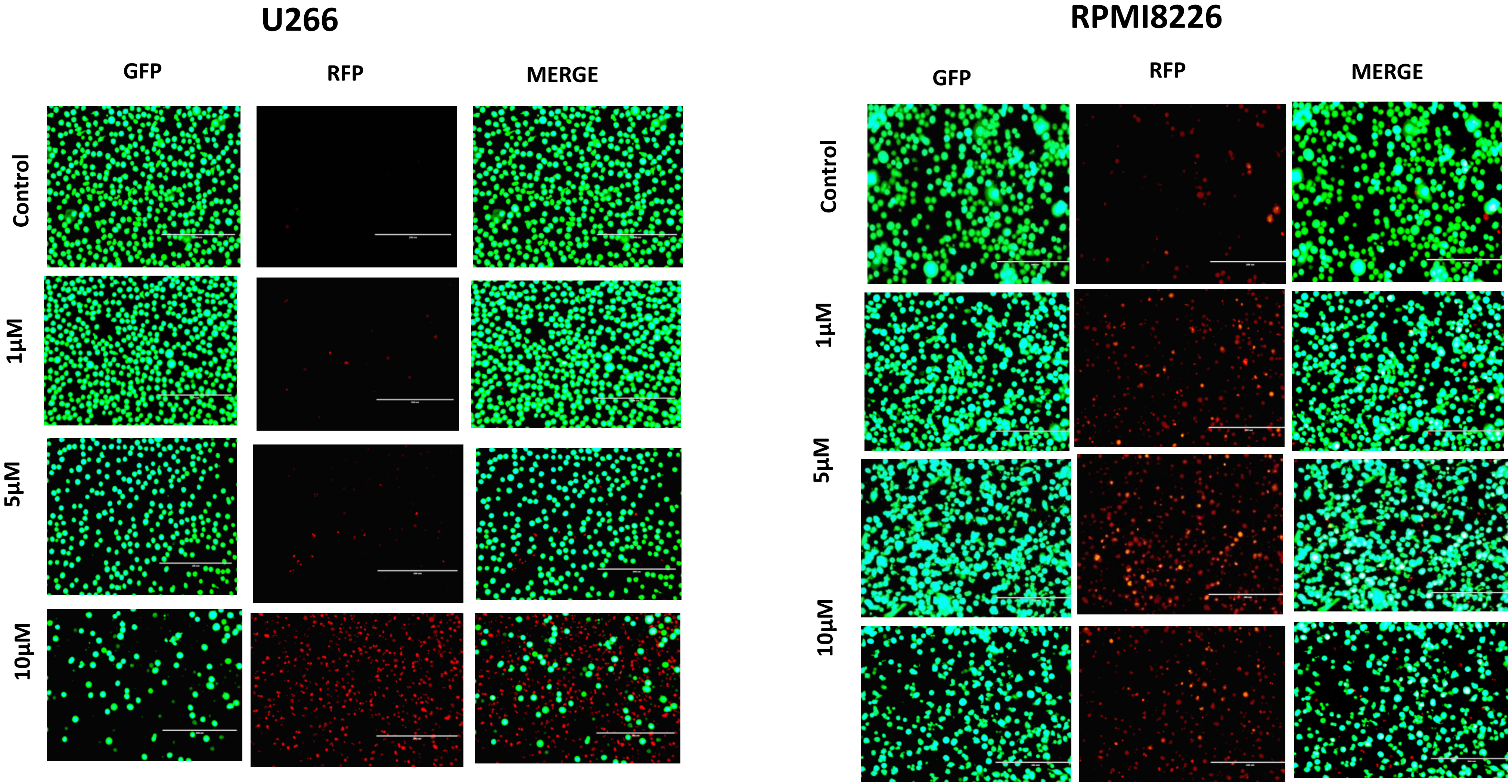

Figure 1

Figure 1 E

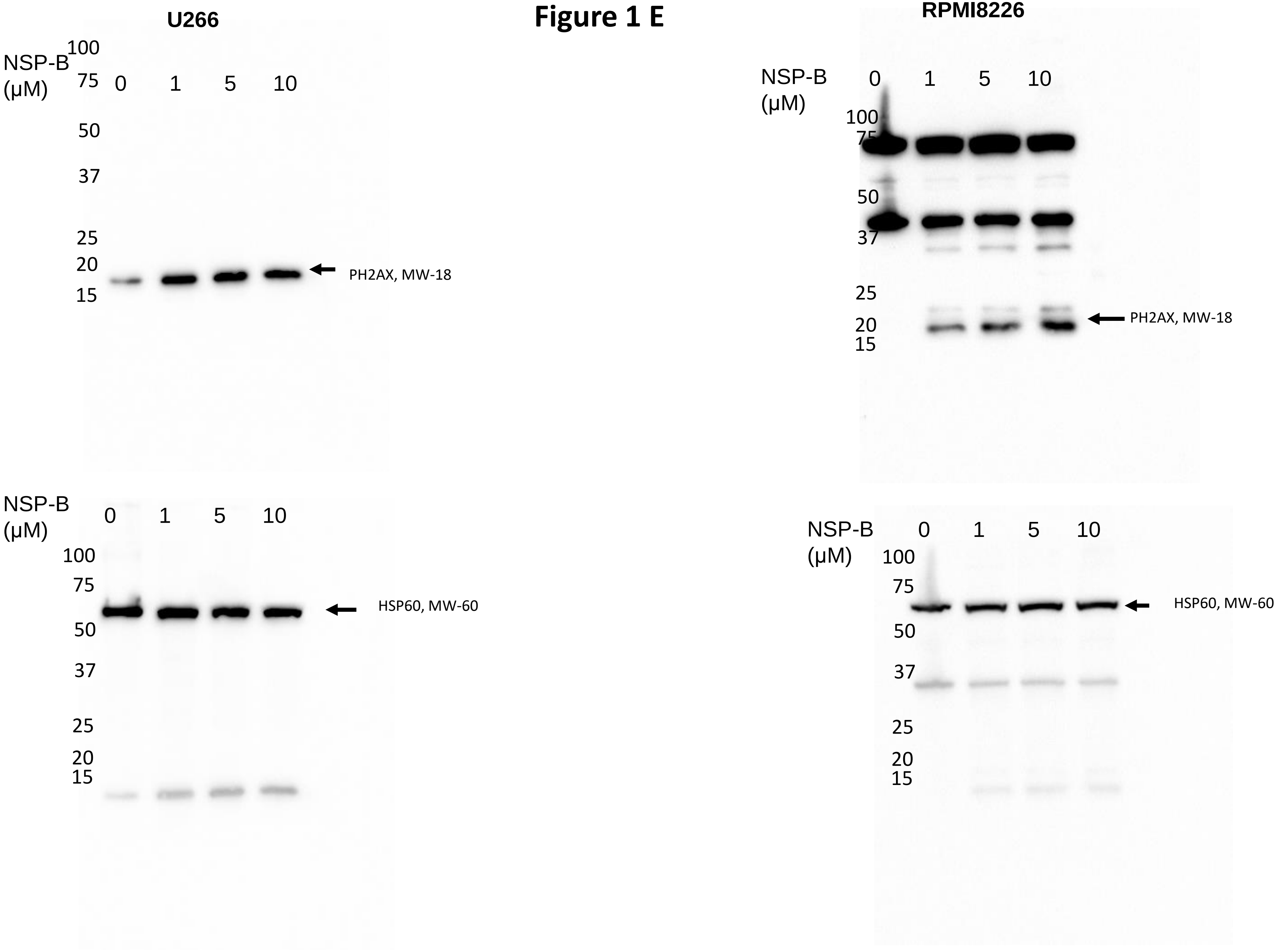

Figure 2A

Figure 2

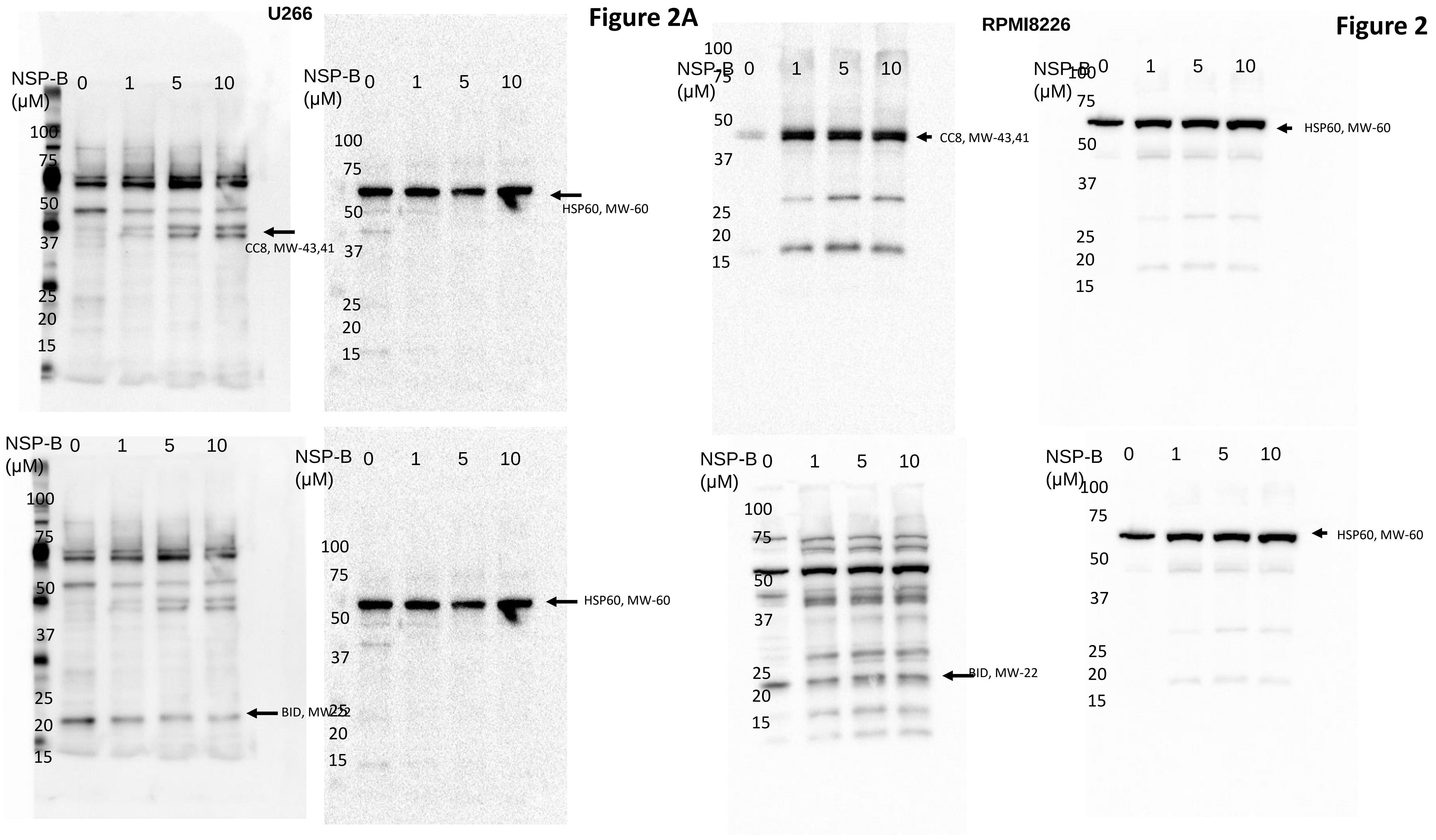

Figure 2A

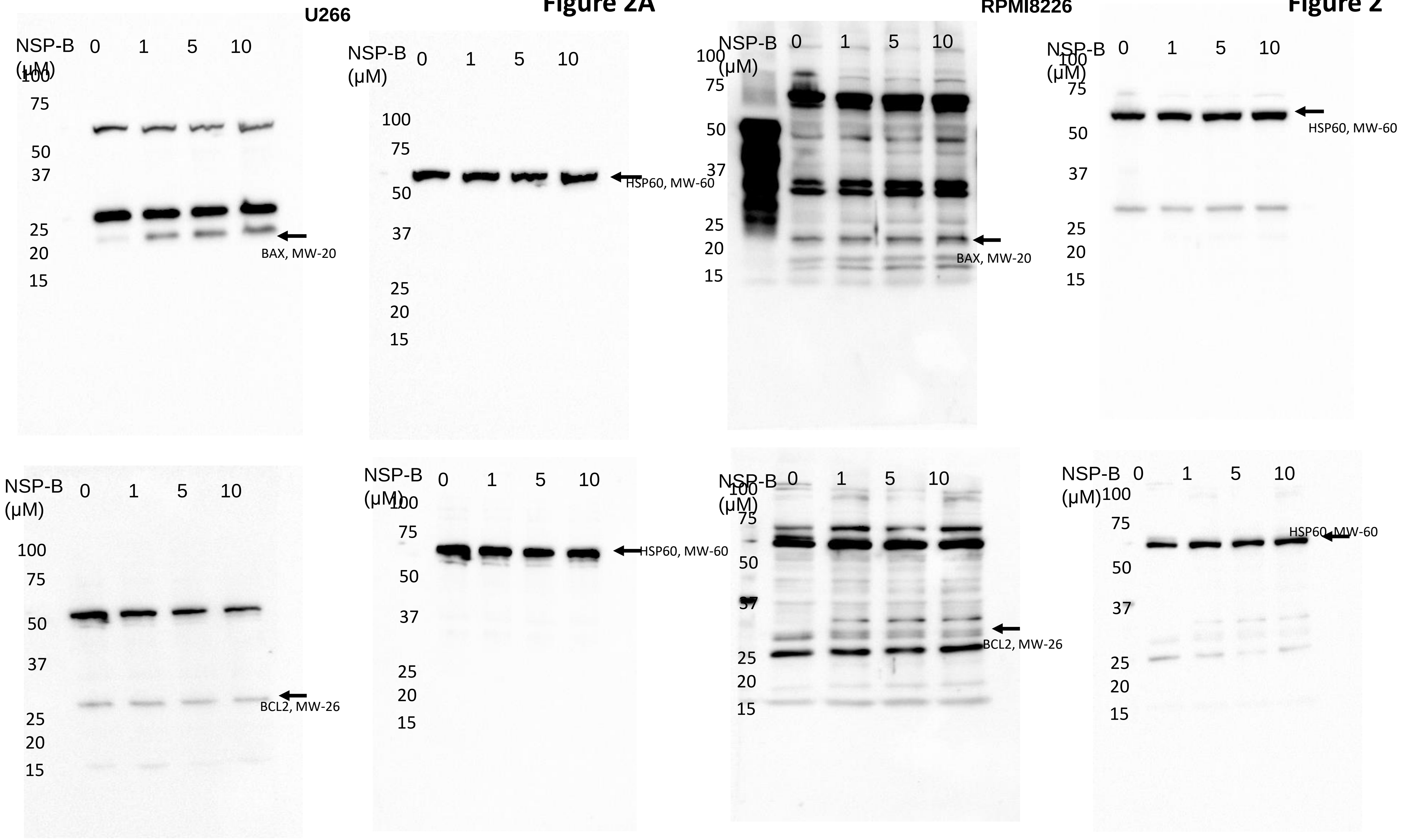

U266
RPMI8226
Figure 2
NSP-B (µM) 0 1 5 10
100
75
50
37
25
20
15
BAX, MW-20
HSP60, MW-60
BCL2, MW-26

Figure 2C

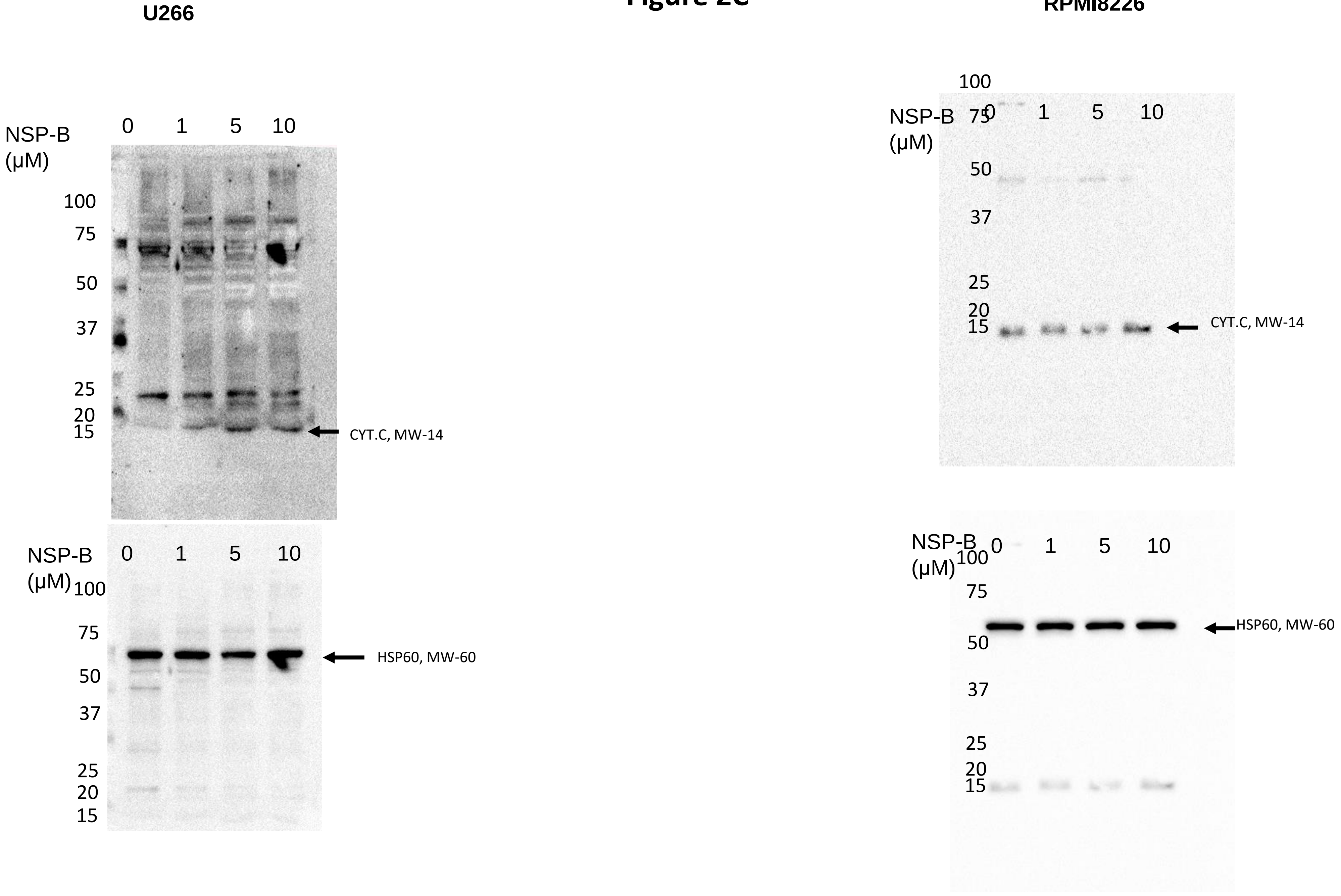
U266
RPMI8226
NSP-B (μM)
0
1
5
10
100
75
50
37
25
20
15
CYT.C, MW-14
HSP60, MW-60

Figure 2

Figure 2 D

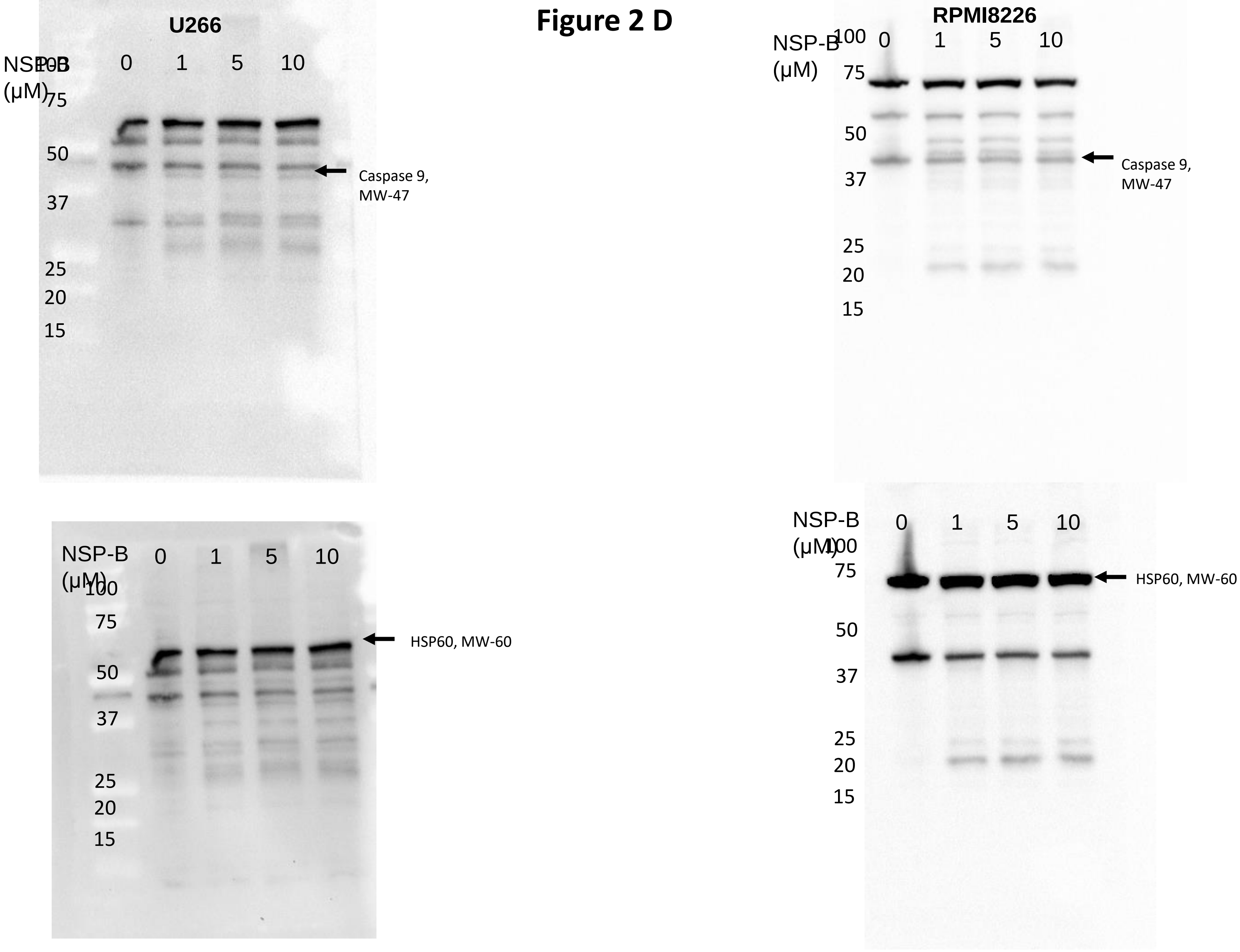

Figure 2

Figure 2 D

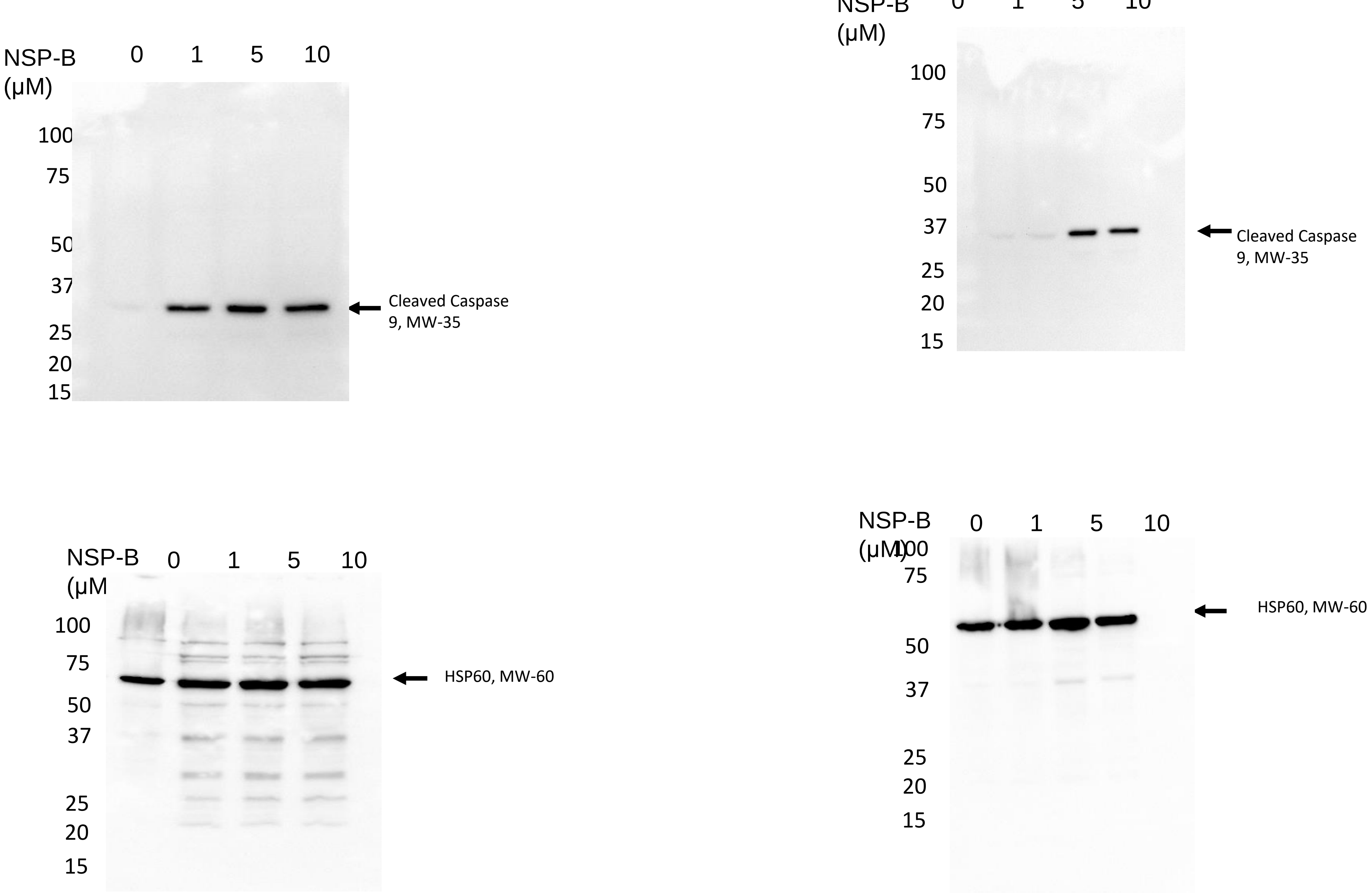

U266

Figure 2 D

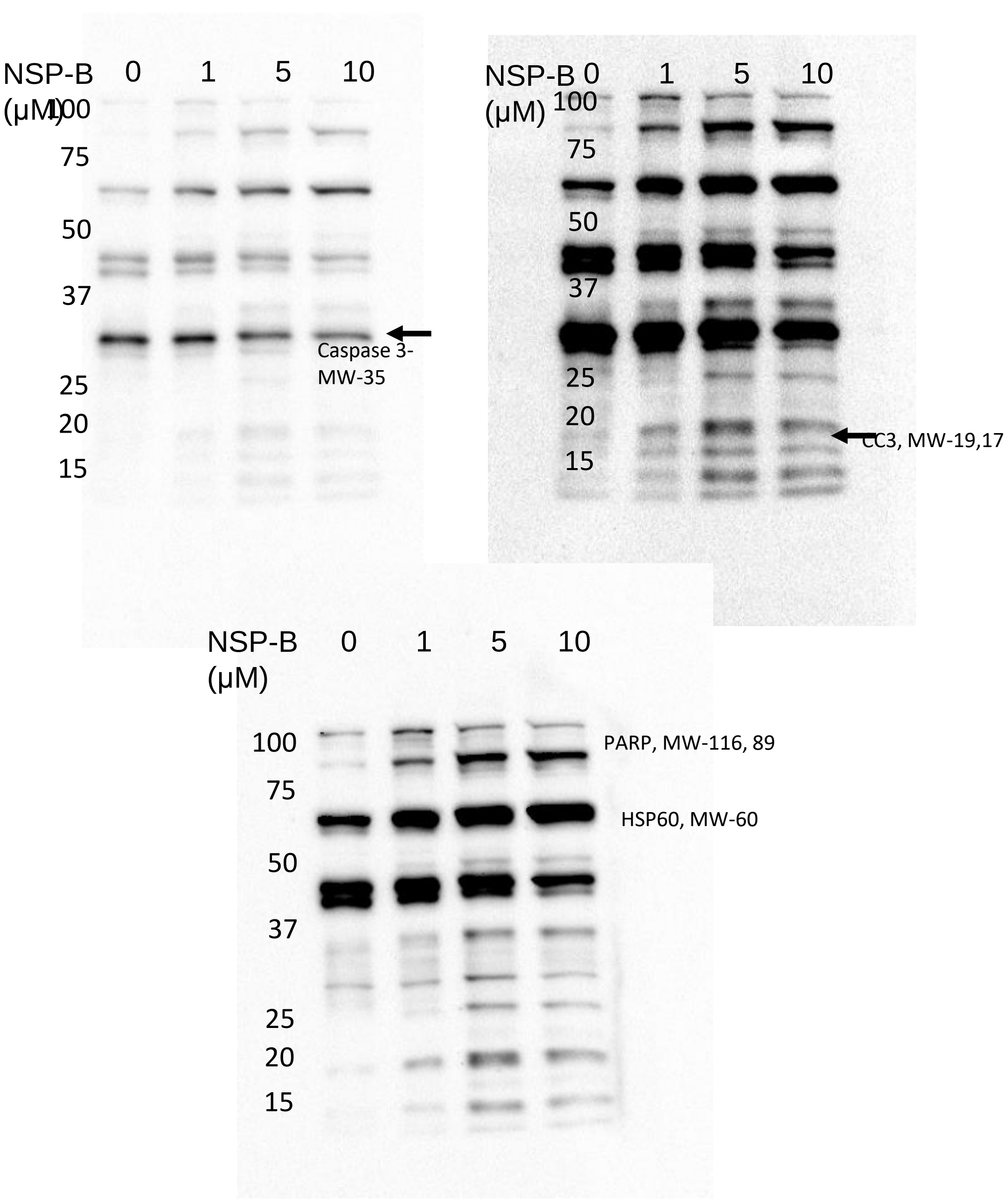

RPMI8226

Figure 2

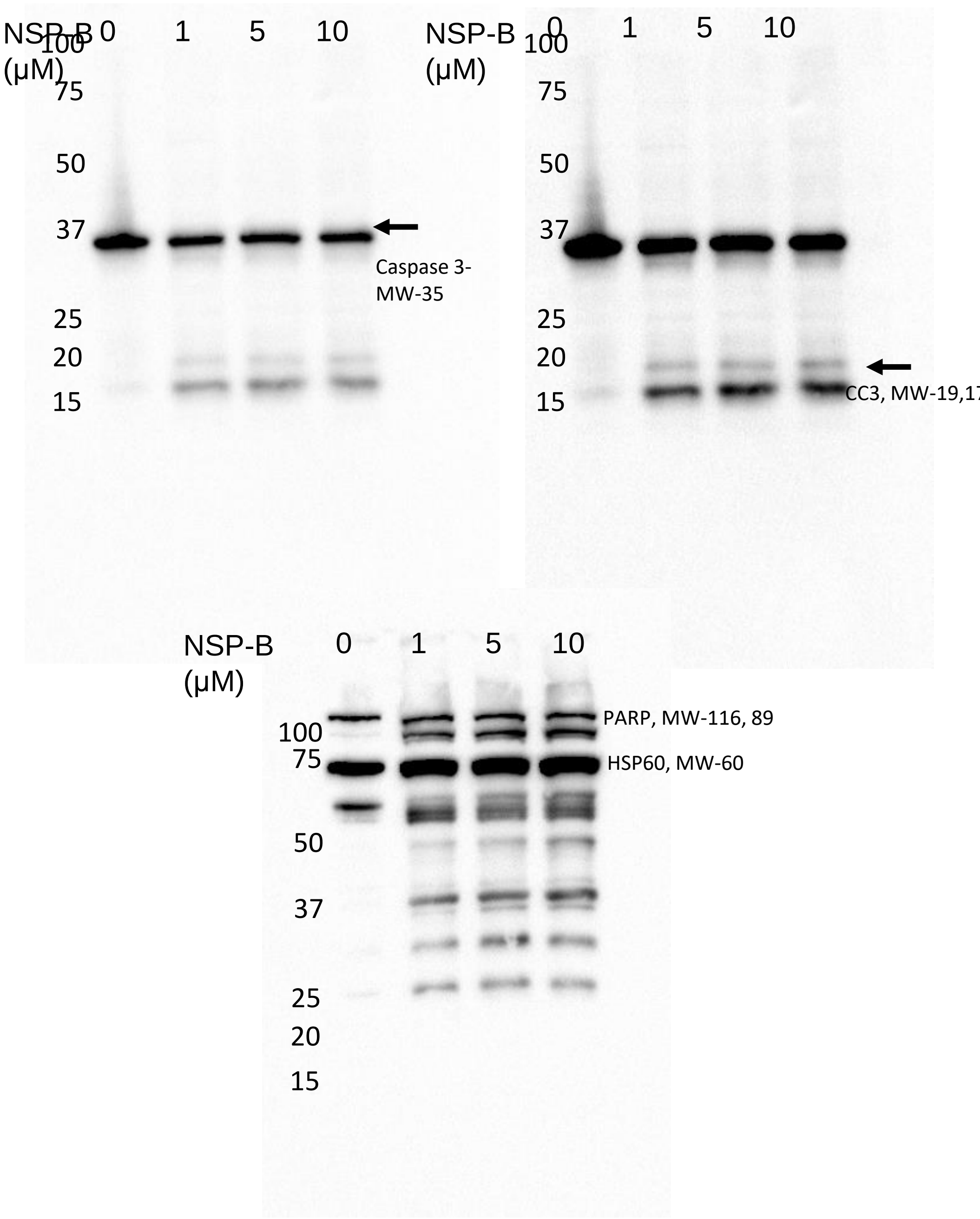

U266

Figure 2 E

RPMI8226

Figure 2

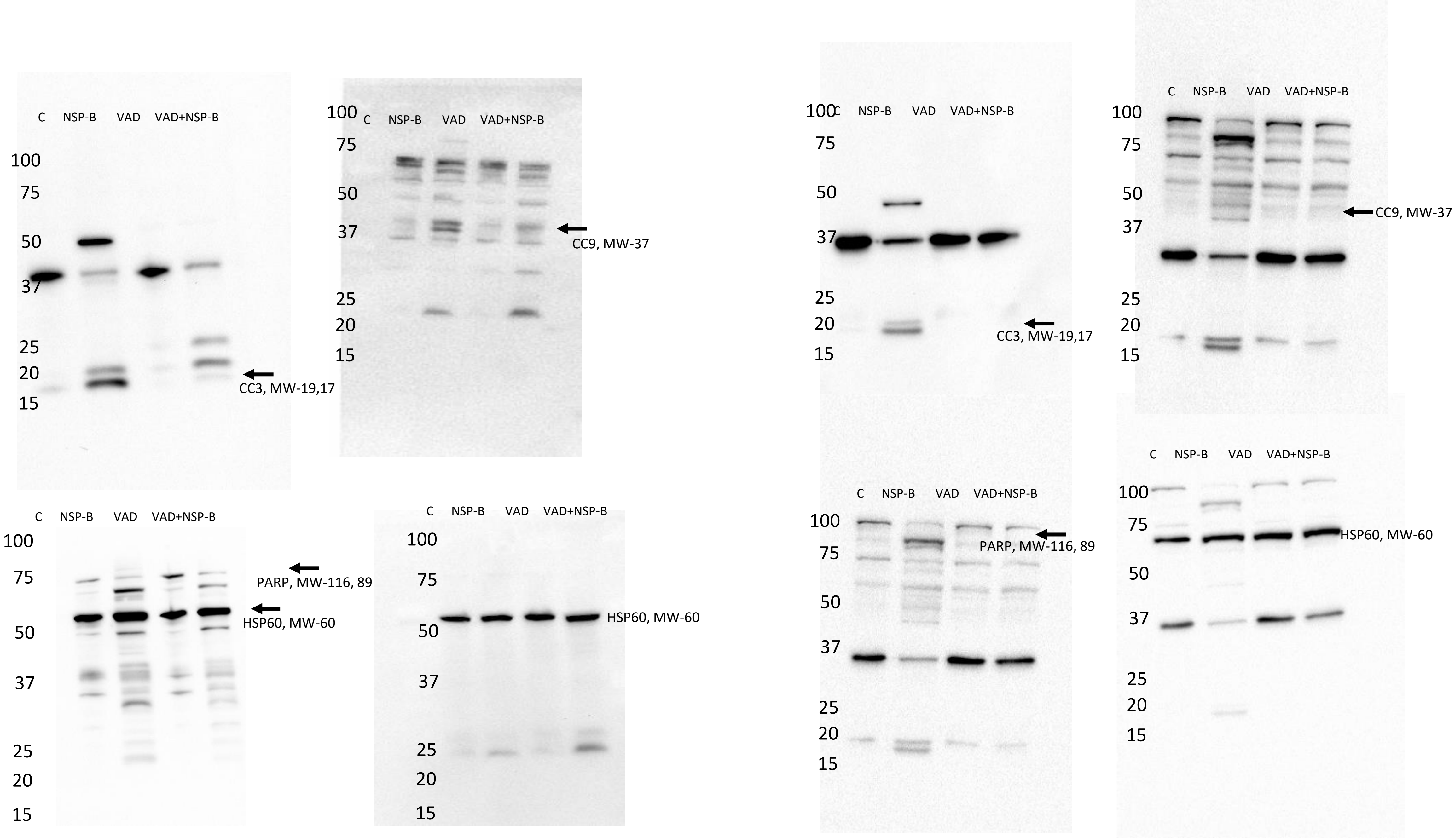

Figure 3 A **Figure 3**

U266 RPMI8226

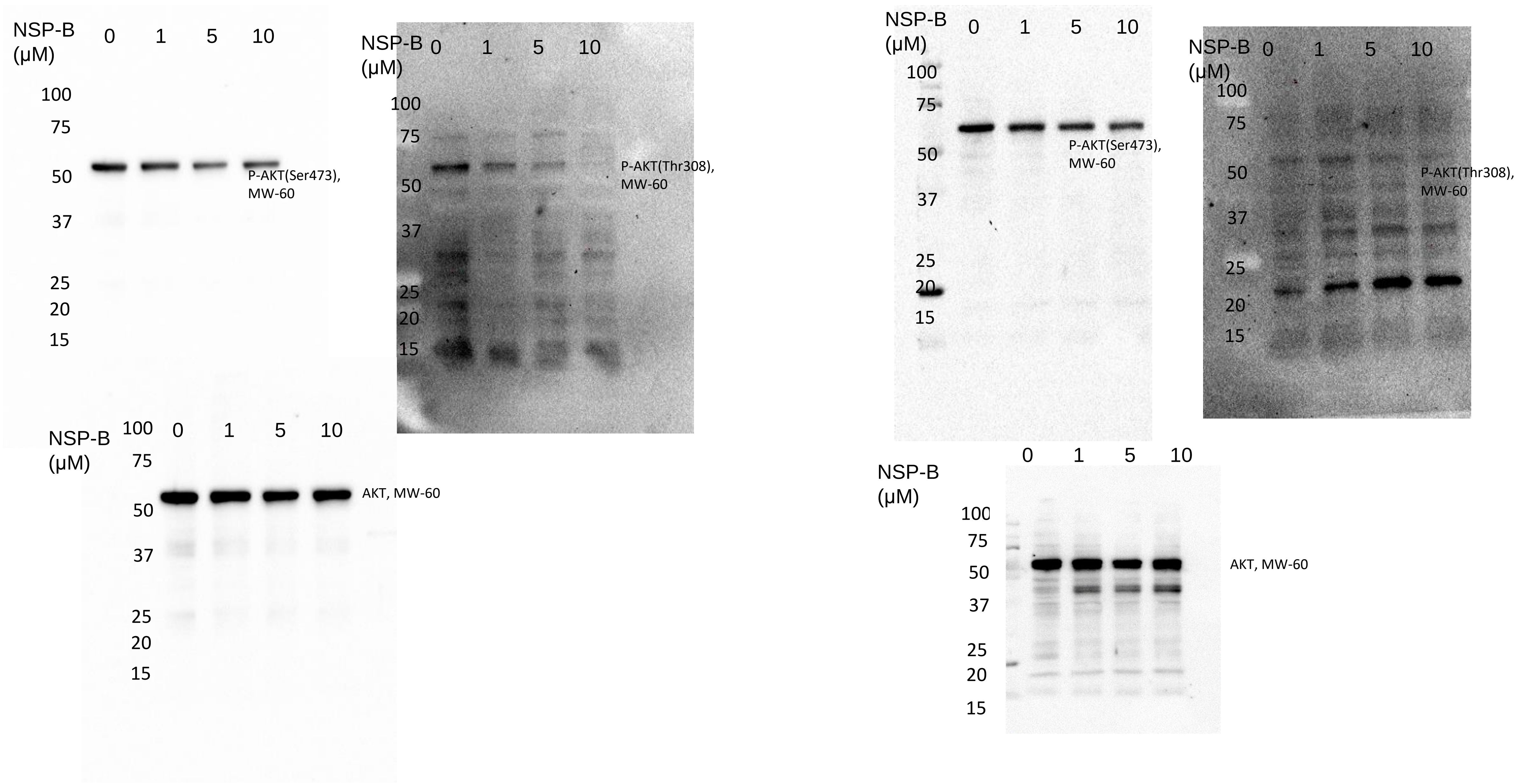

U266

Figure 3 A

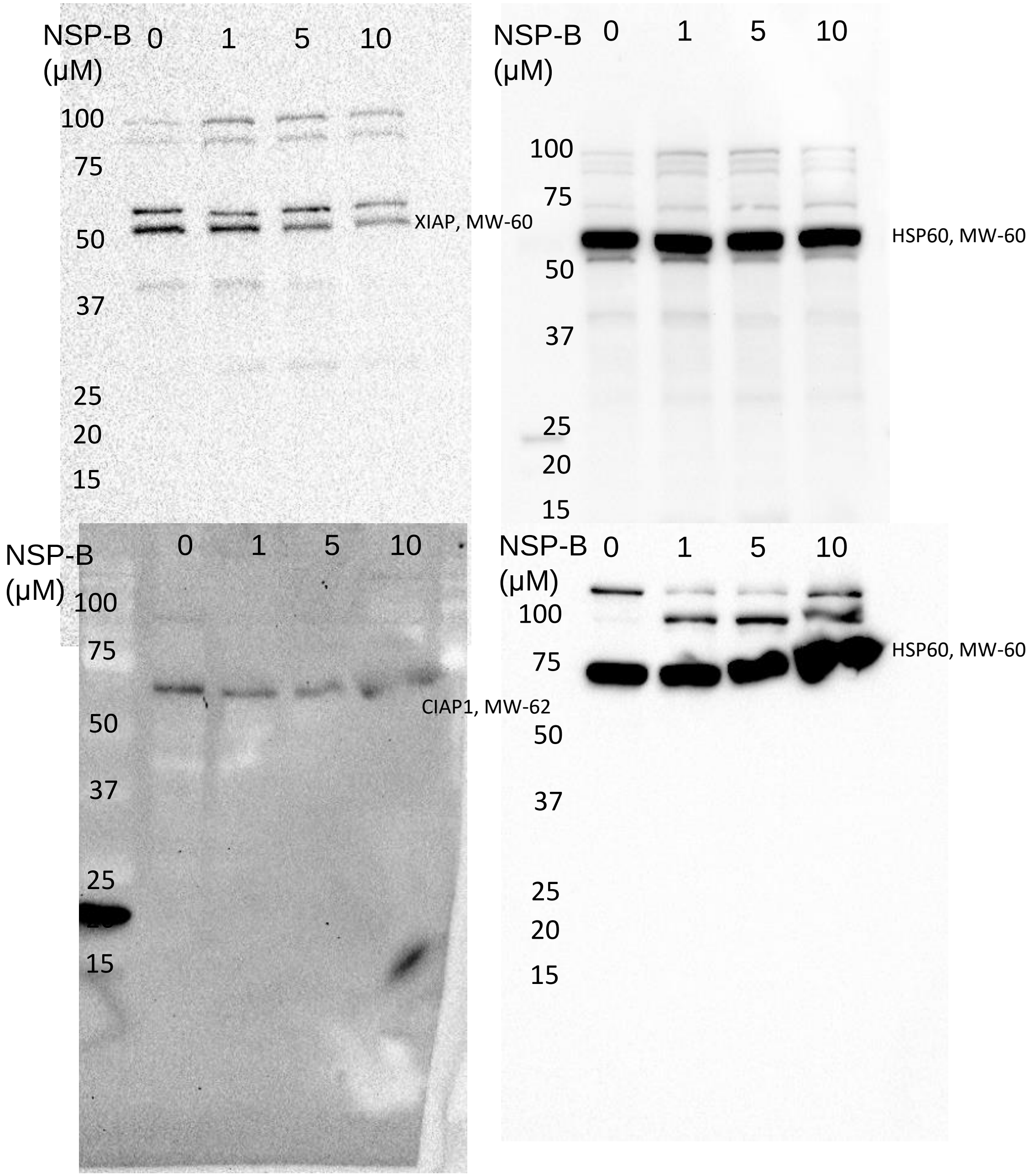

RPMI8226

Figure 3

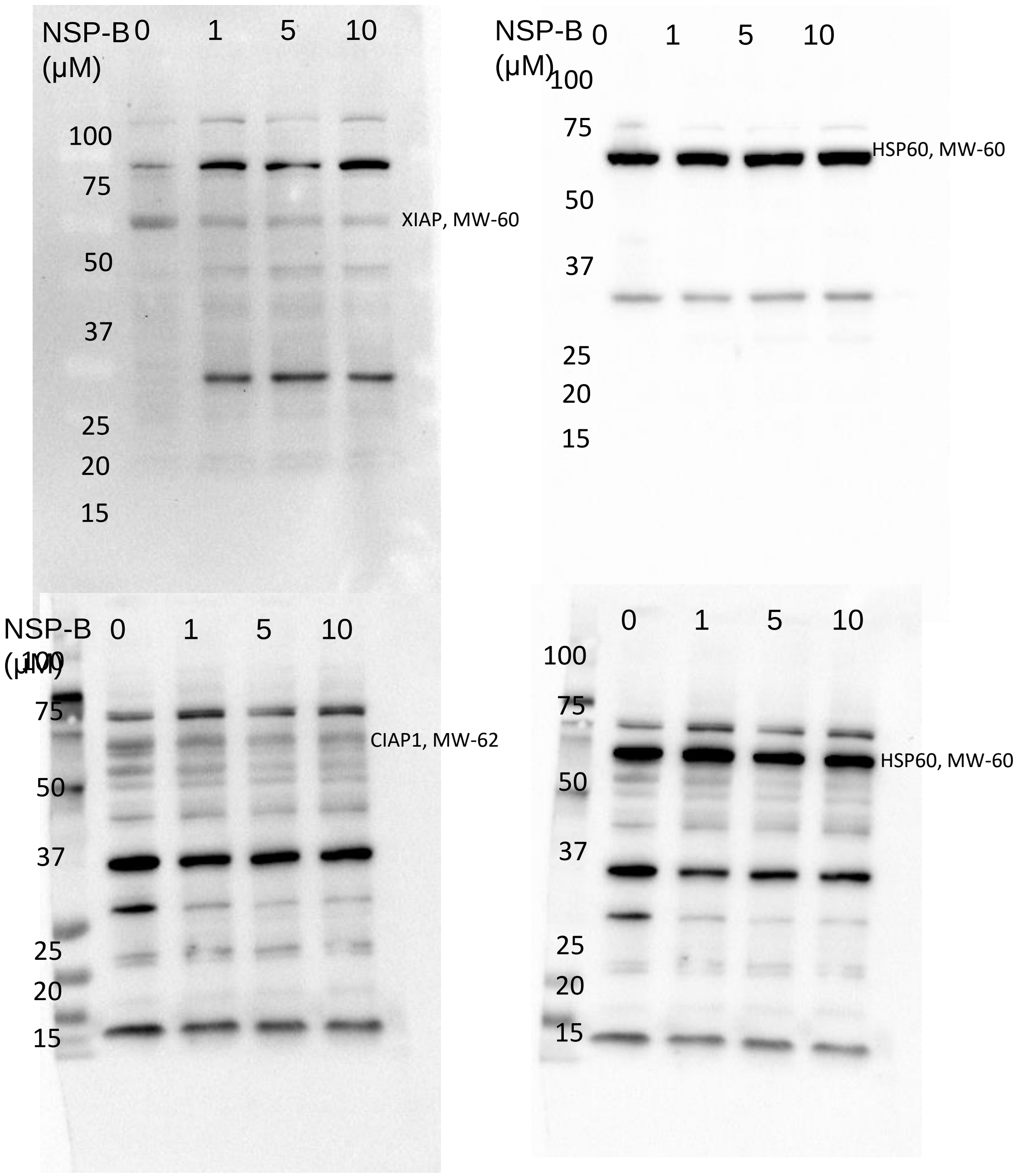

Figure 3

Figure 3 A

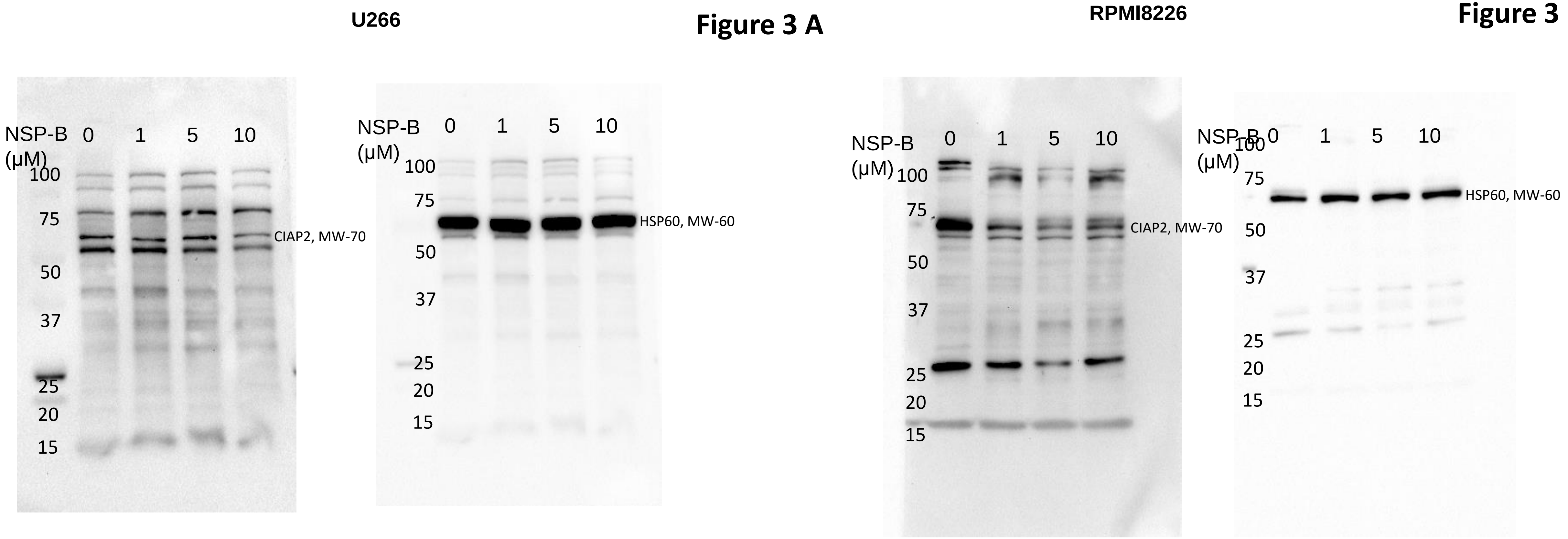

Figure 3

Figure 3 B

U266 RPMI8226

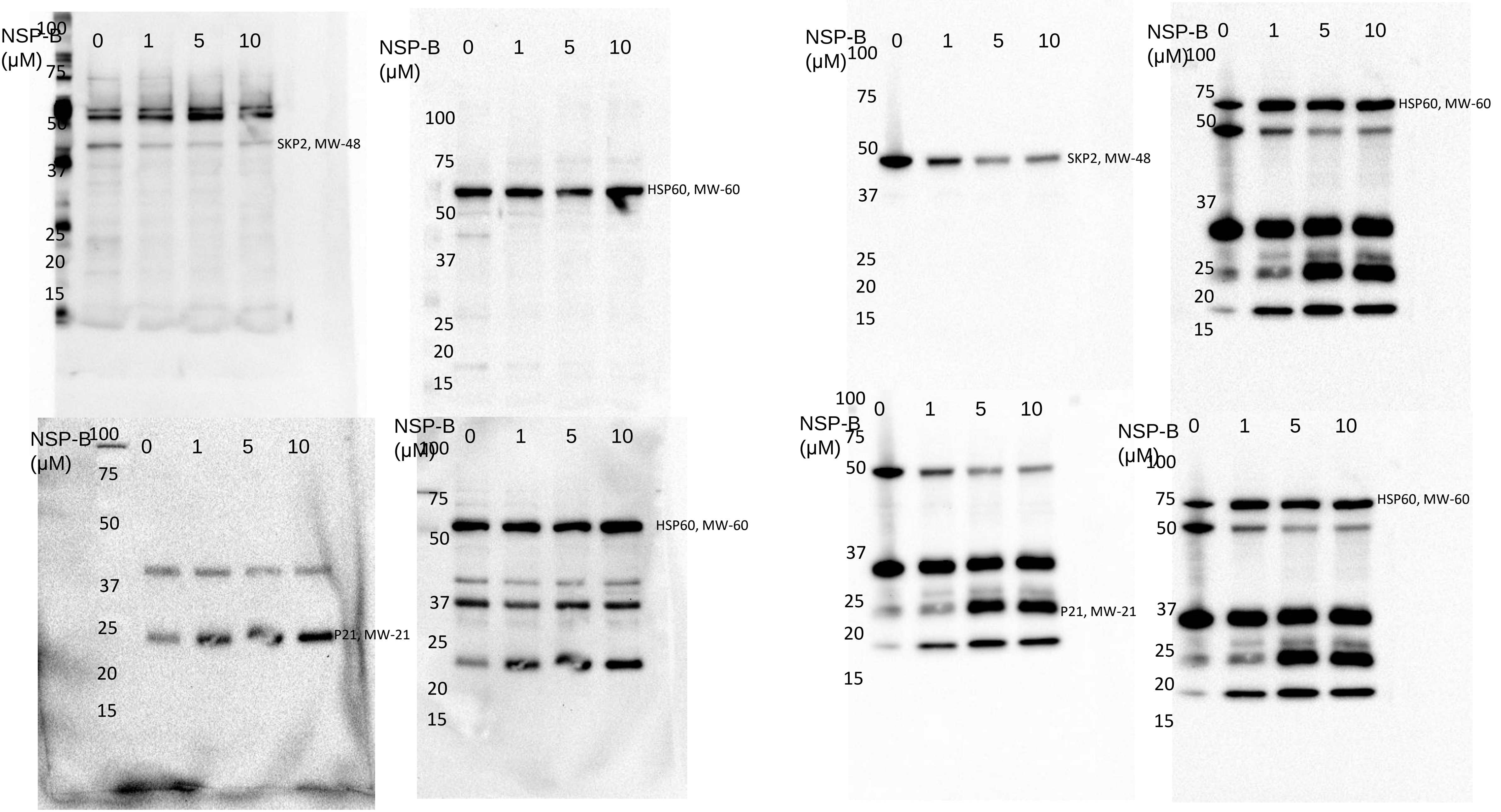

U266

Figure 3 B

RPMI8226

Figure 3

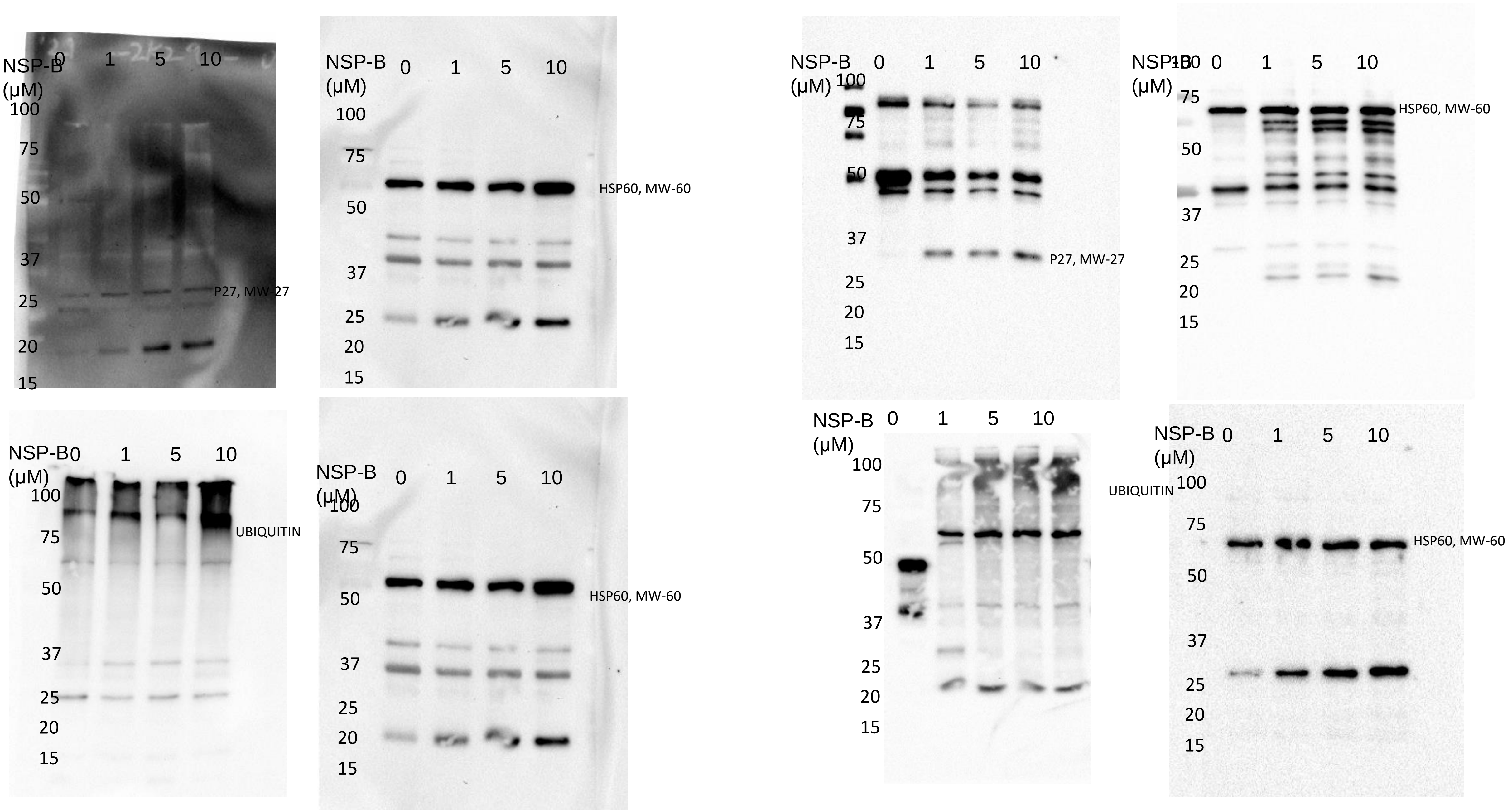

Figure 3 C

Figure 3

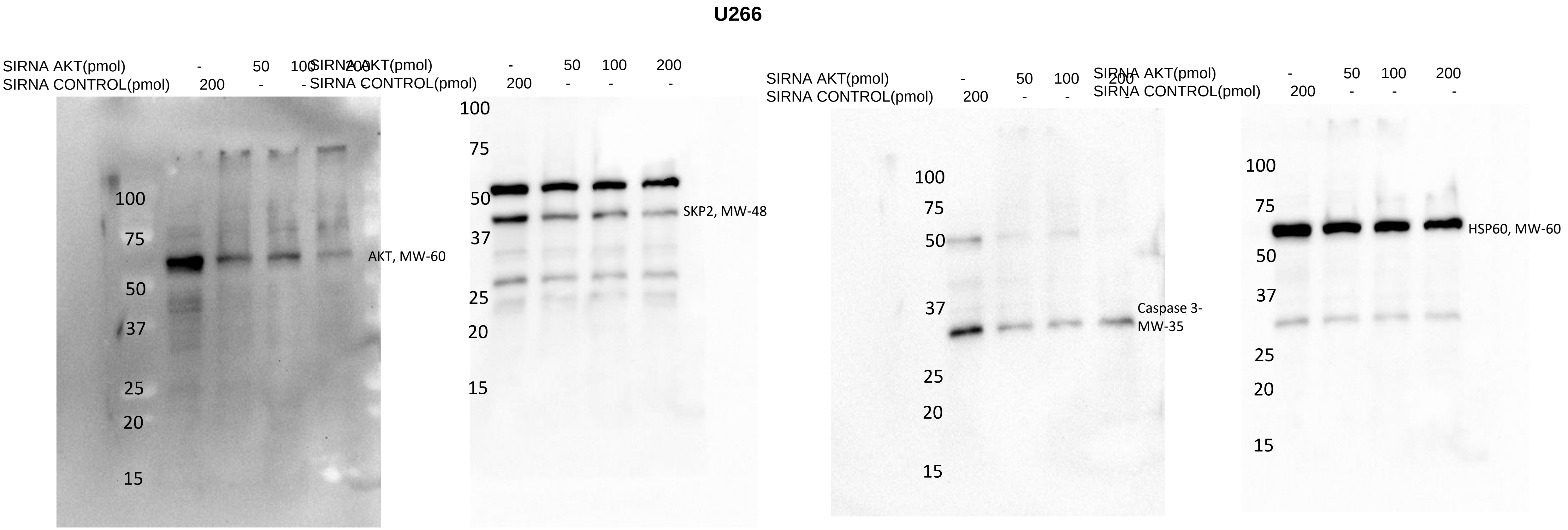

Figure 4

U266

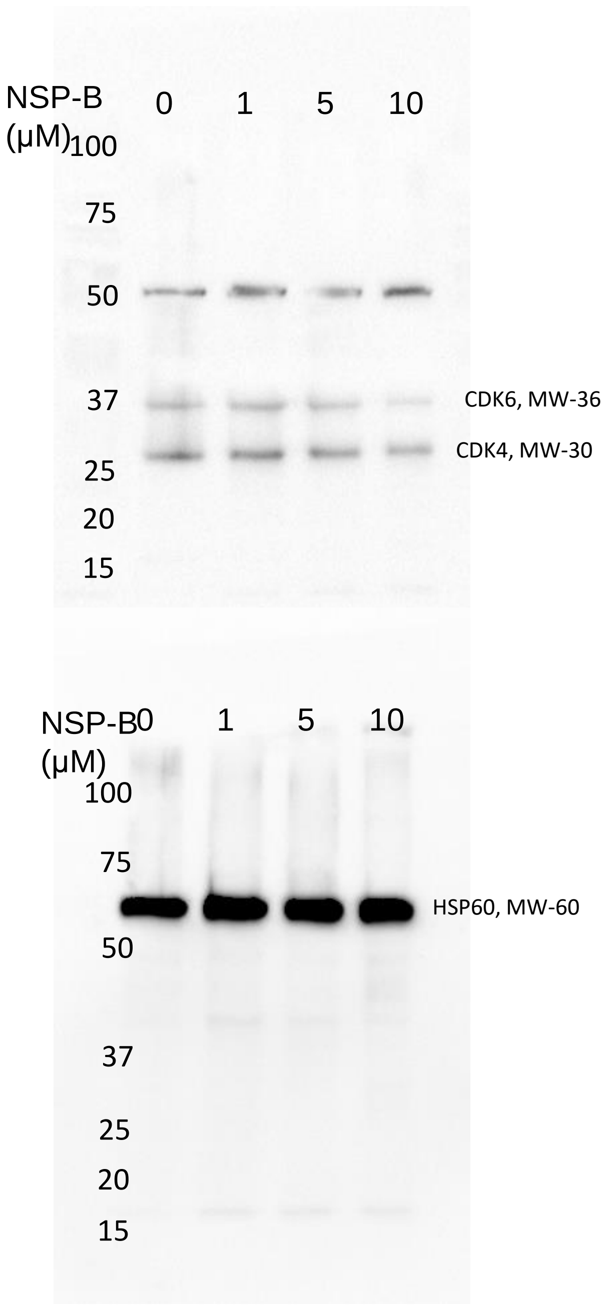

RPMI8226

Figure 4

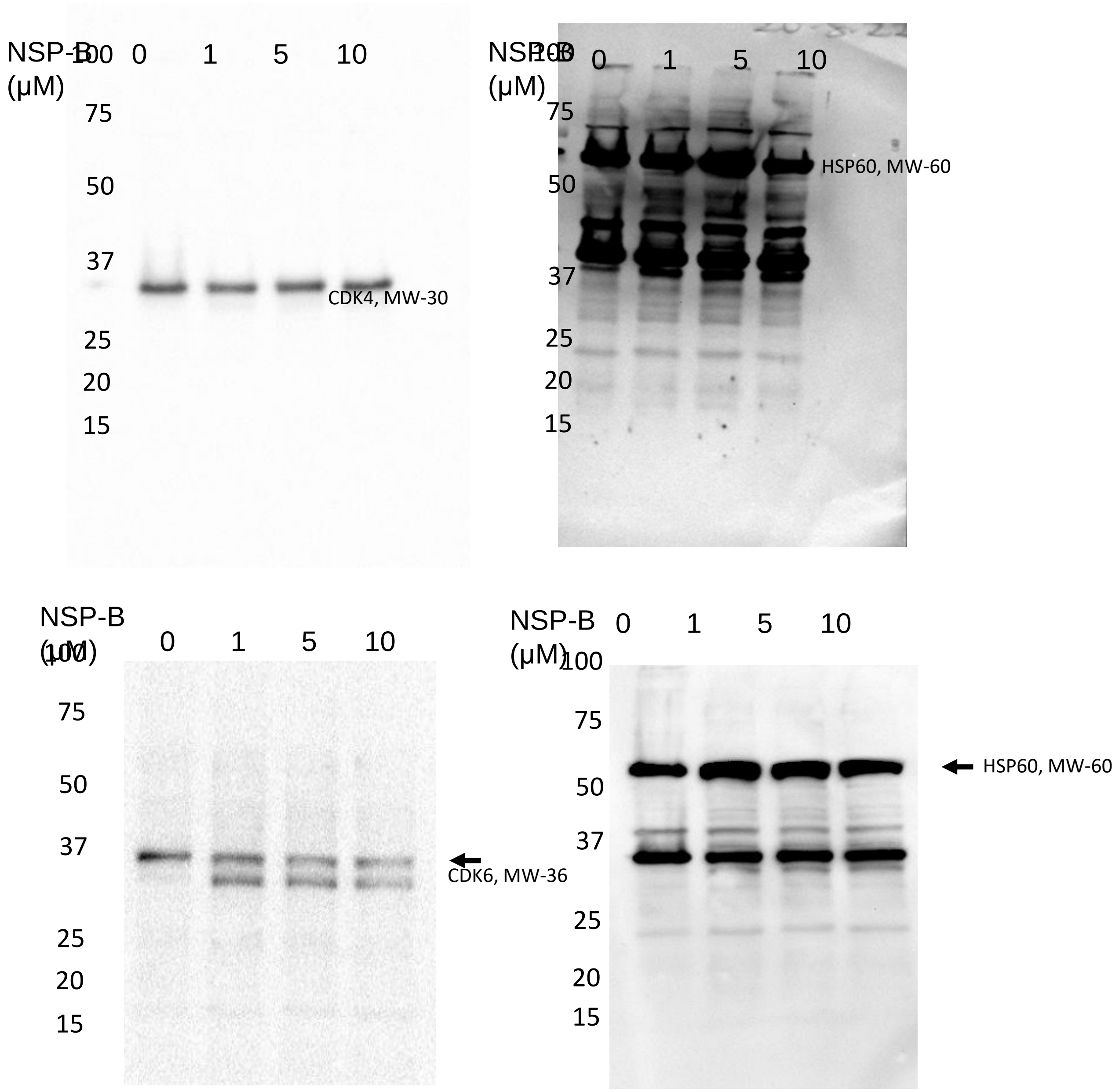

Figure 4

U266

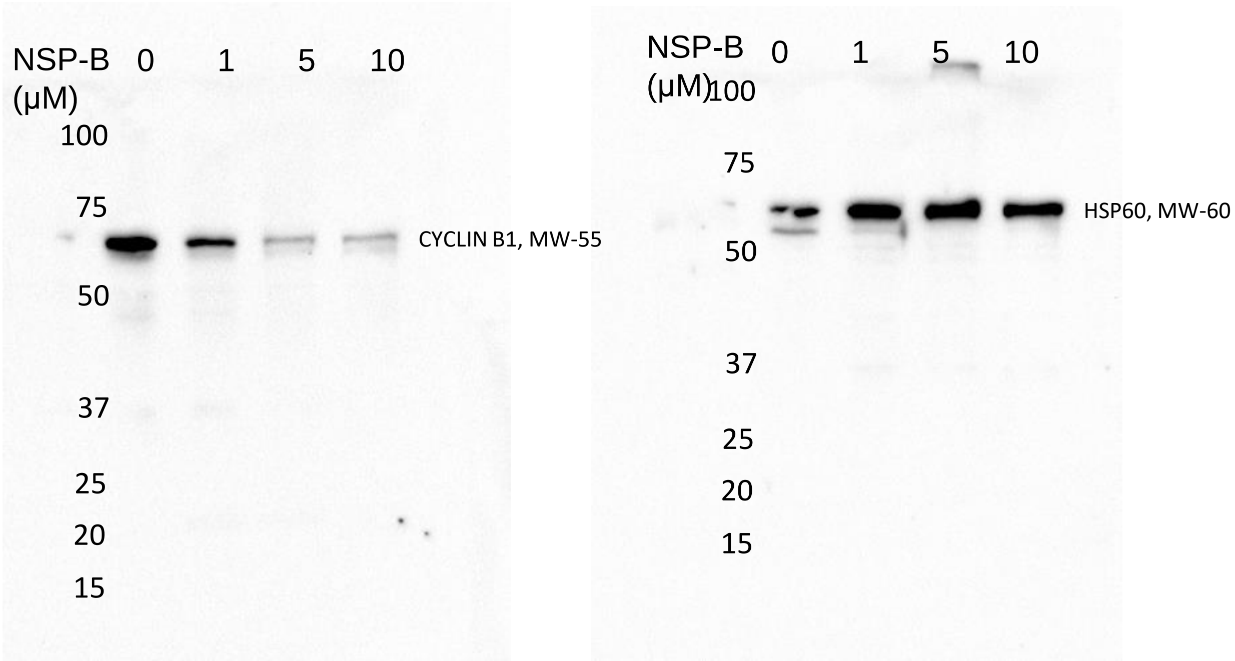

Figure 4

RPMI8226

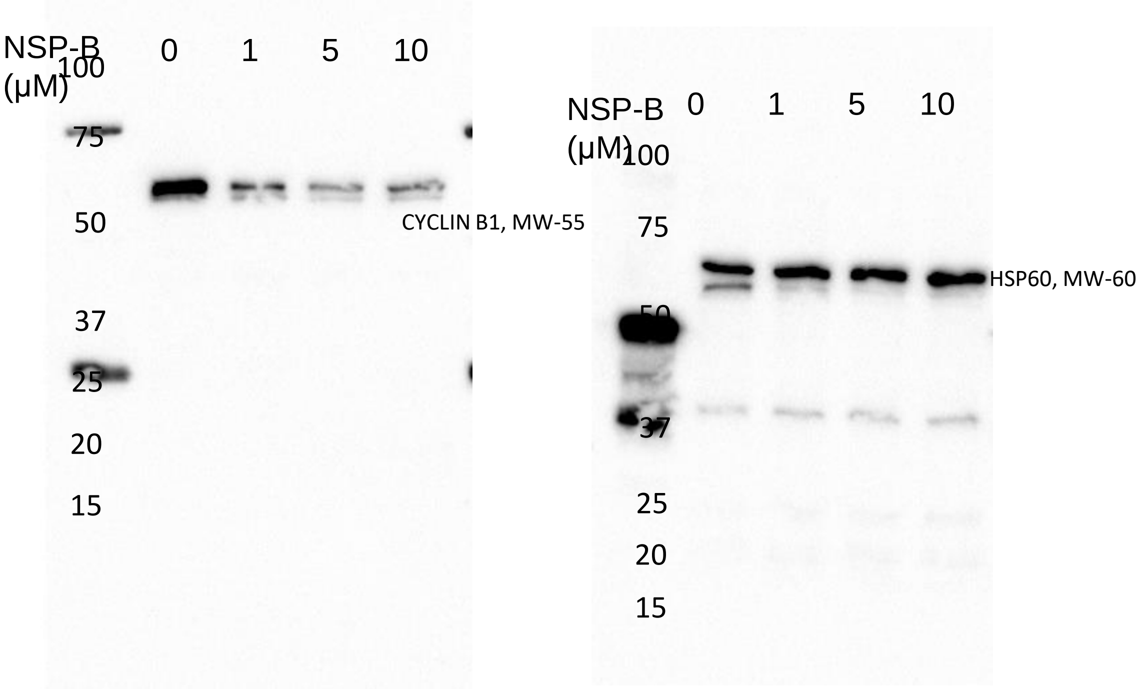

U266 **Figure 5A**

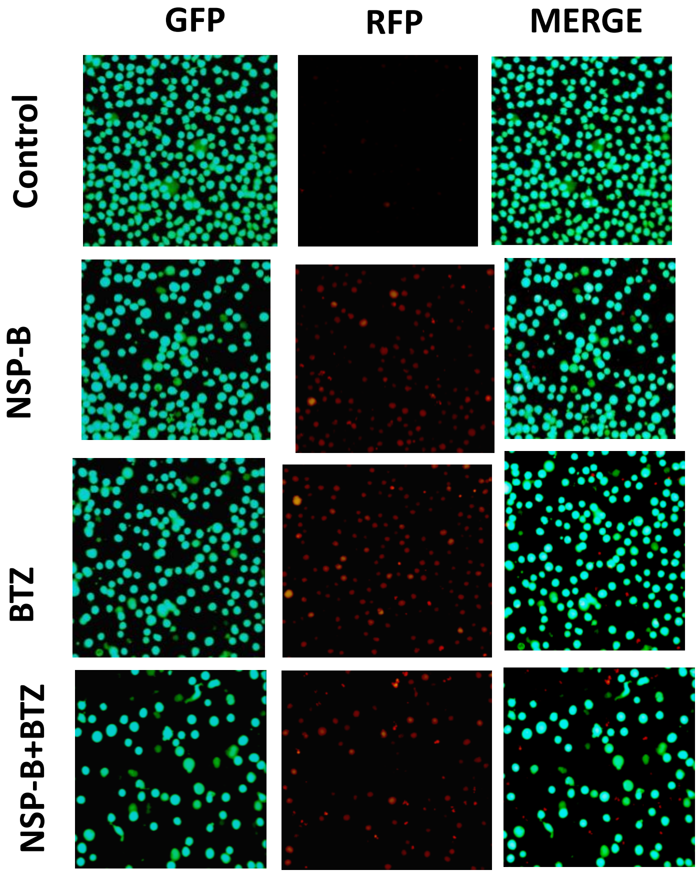

RPMI8226 **Figure 5**

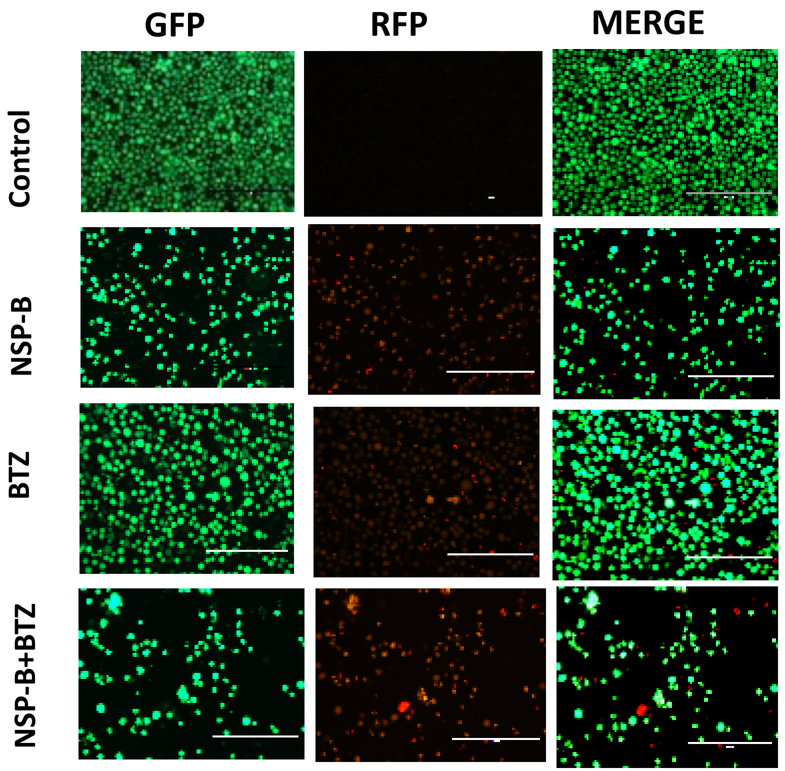

Figure 5B

Figure 5

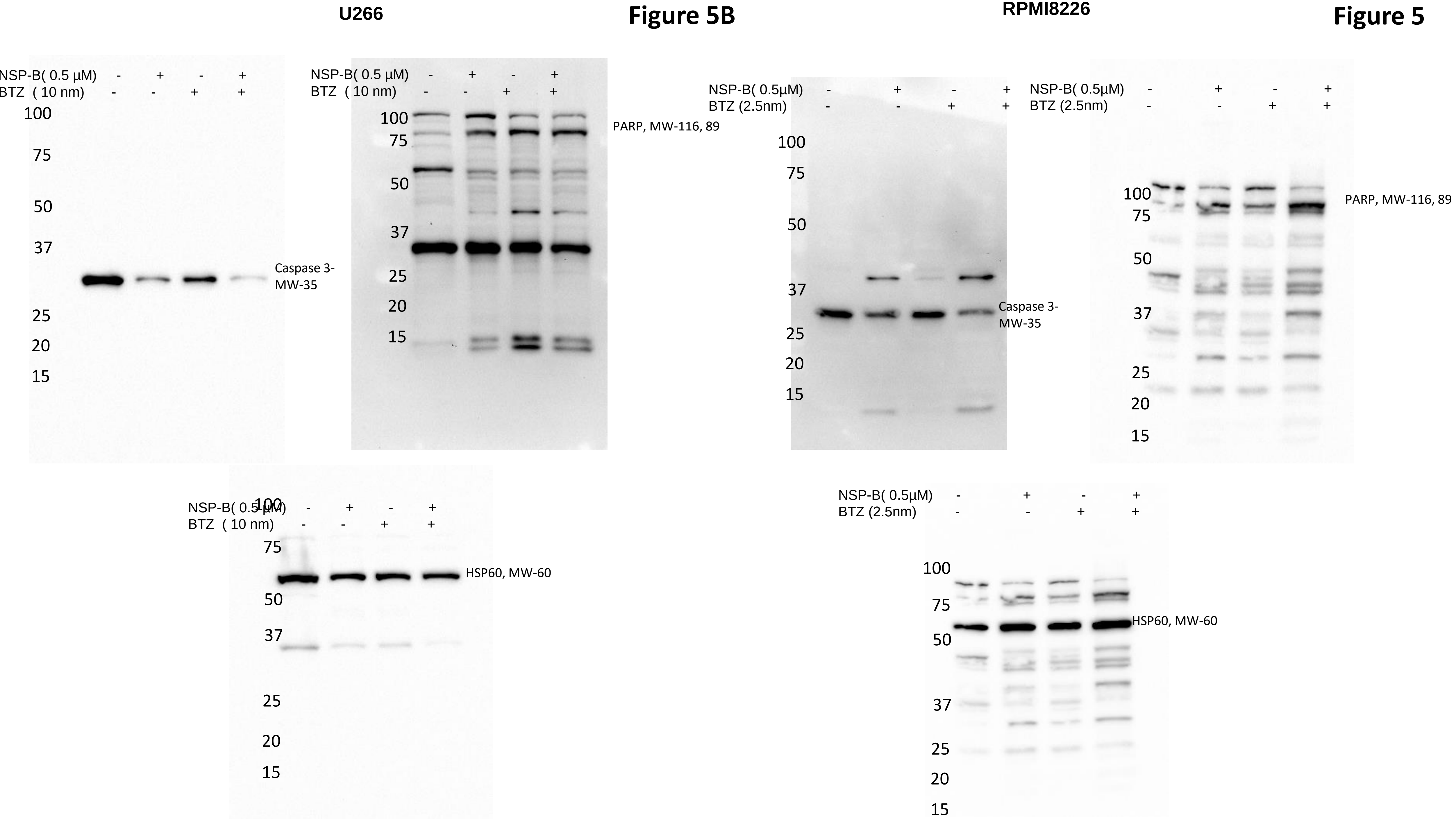

Supplementary Figure 2

Suppl. Fig. 2C

U266

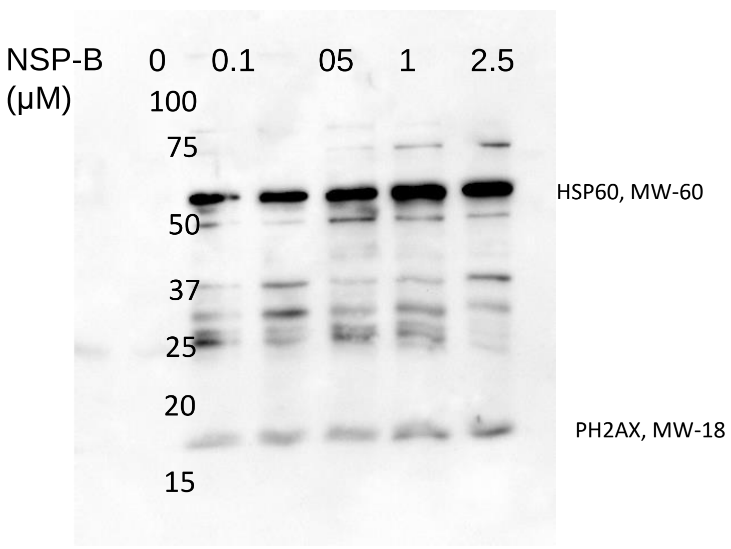

RPMI8226

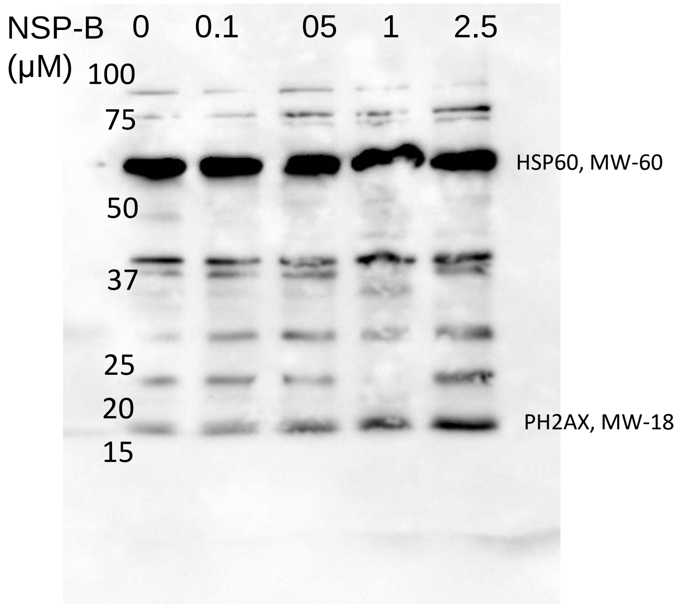

U266

Suppl. Fig. 4

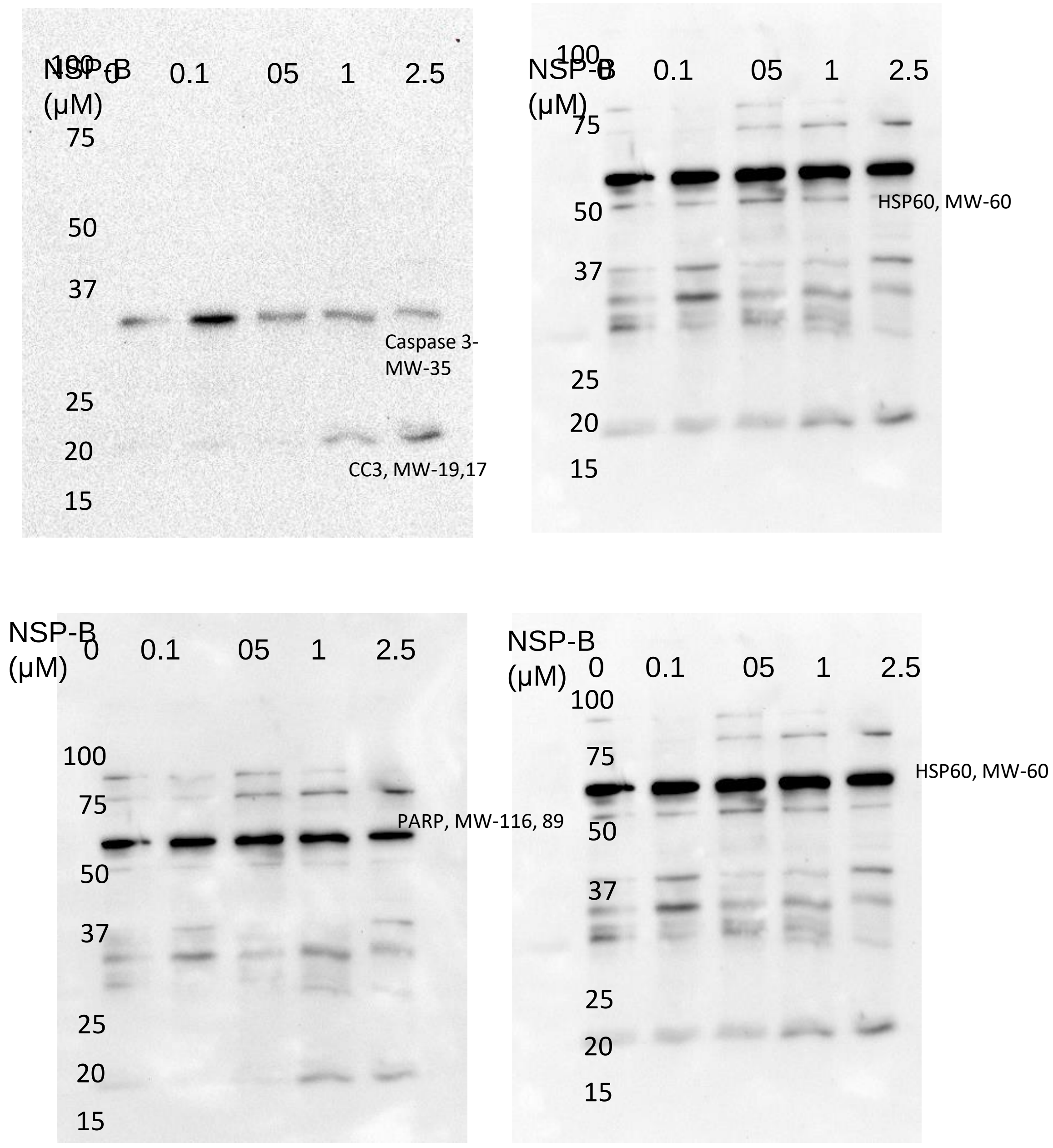

RPMI8226

Supplementary Figure 4

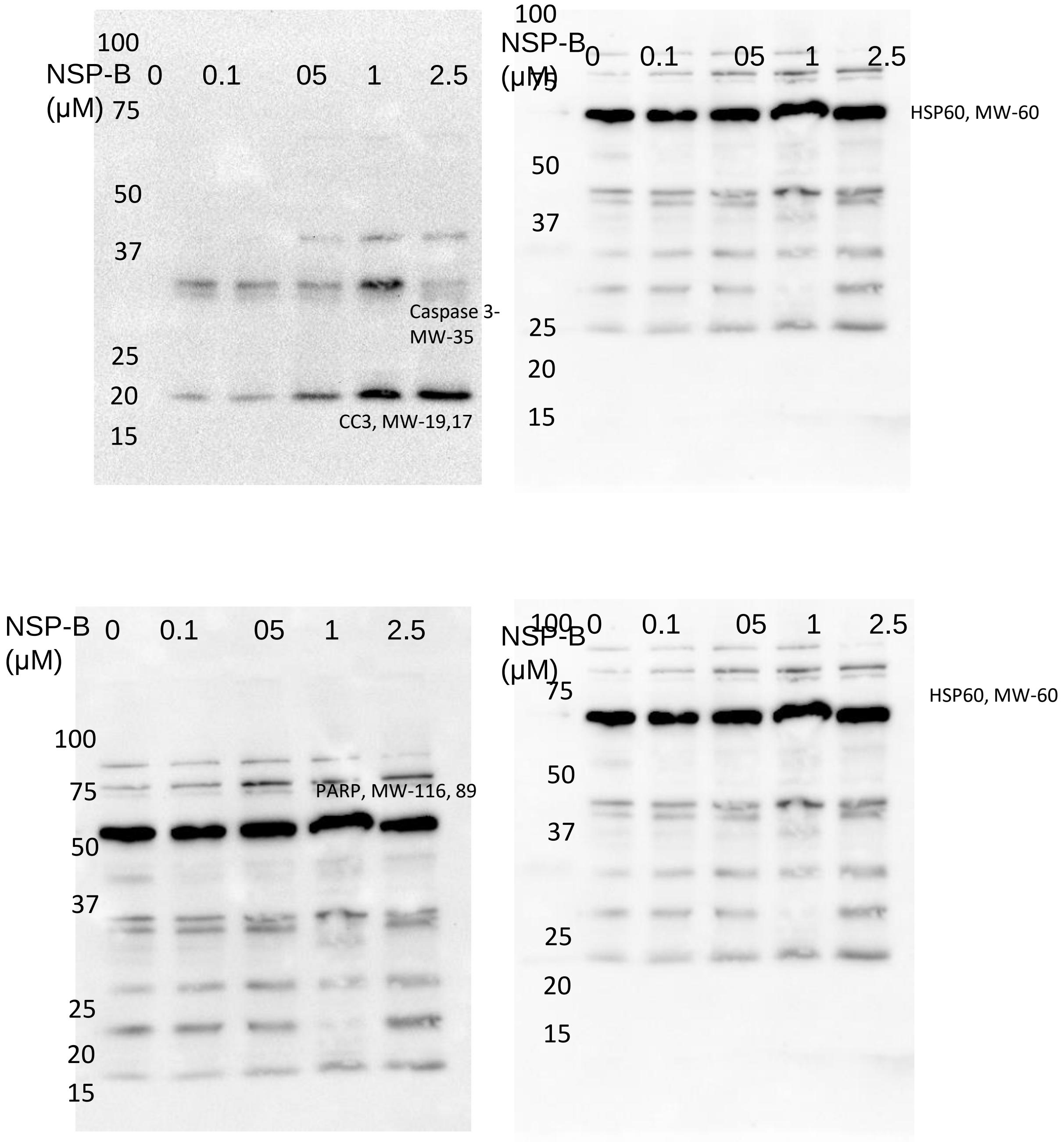

U266 **Suppl. Fig. 7A** RPMI8226 **Supplementary Figure 7**

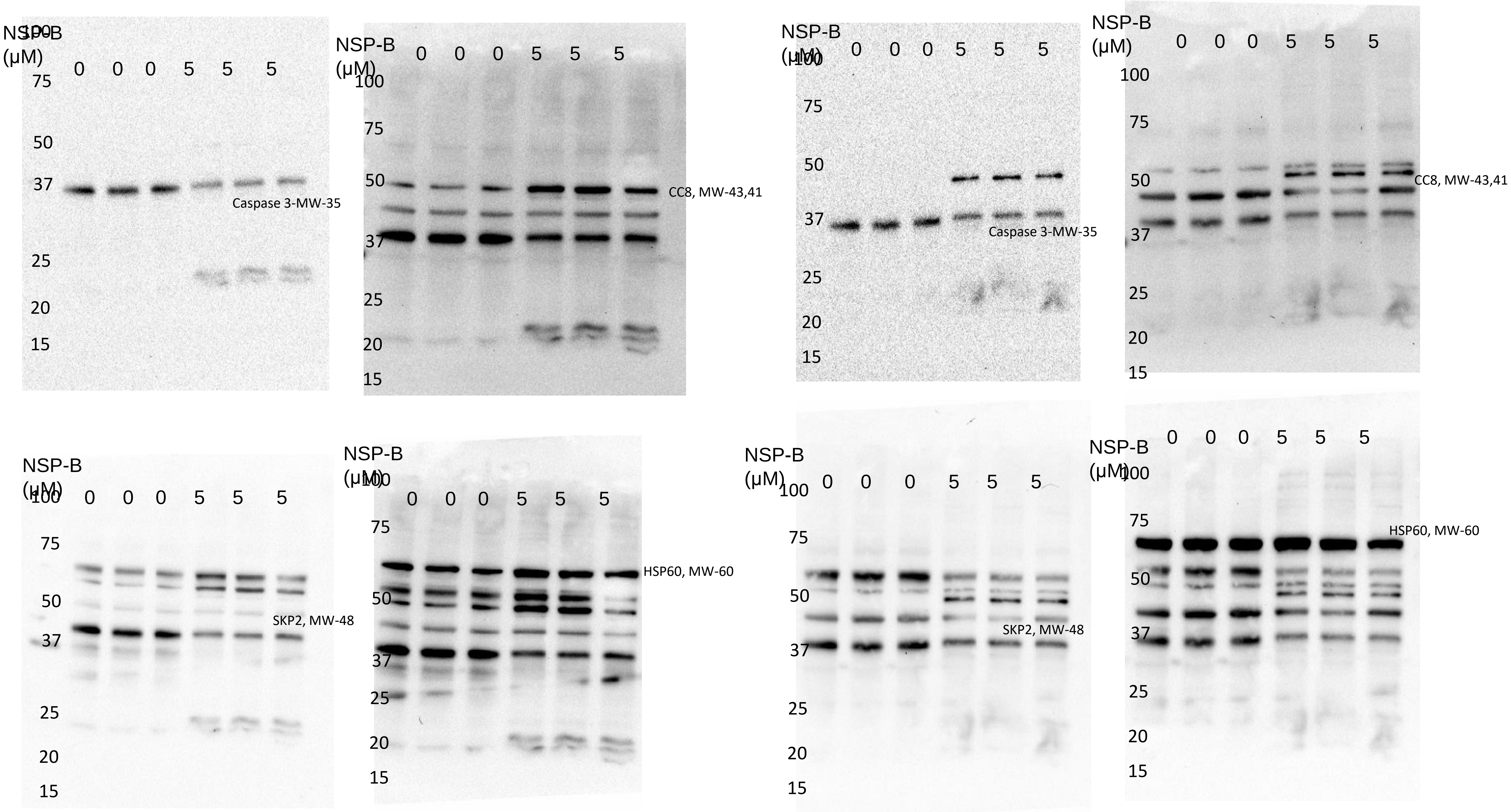

Supplementary Figure 7

Suppl. Fig. 7A

U266

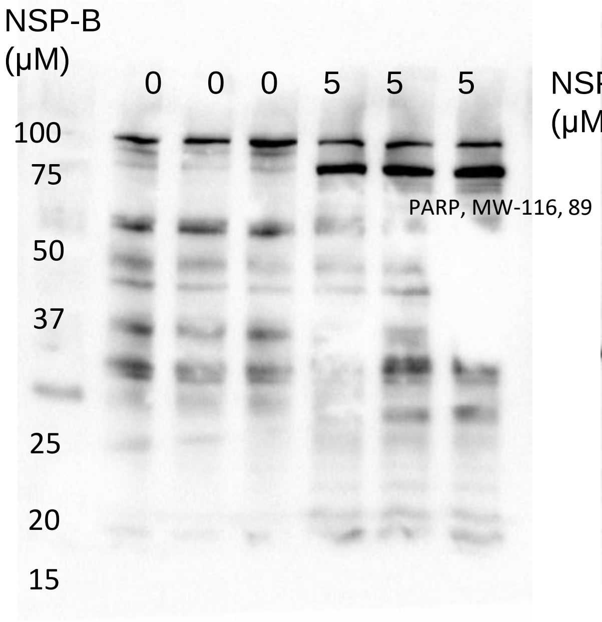

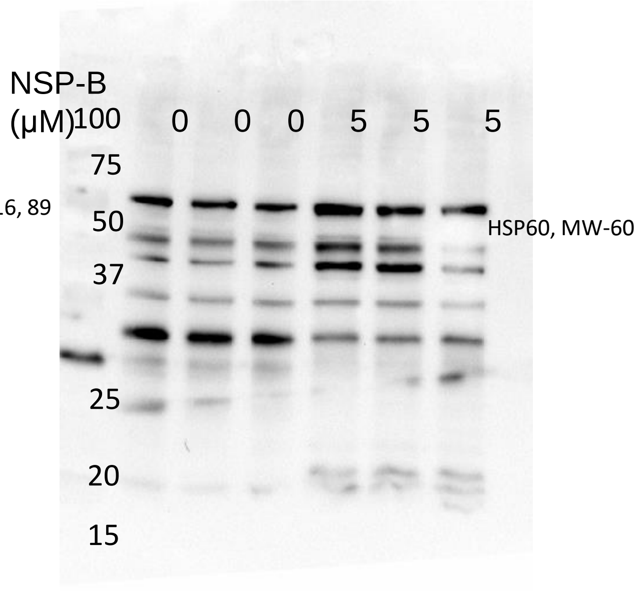

RPMI8226

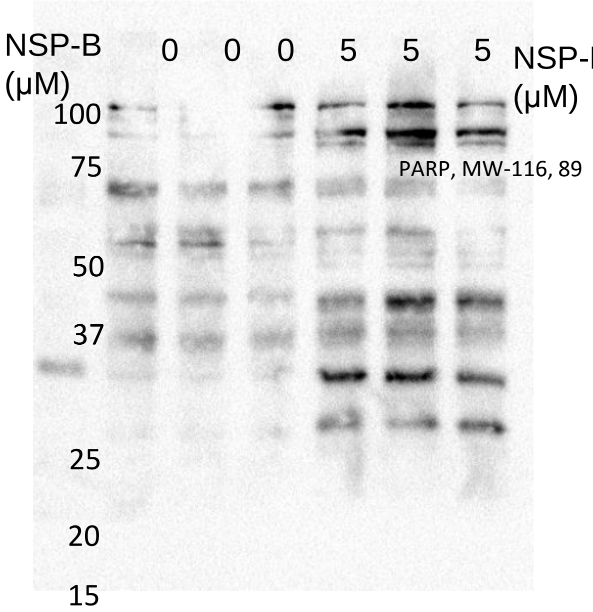

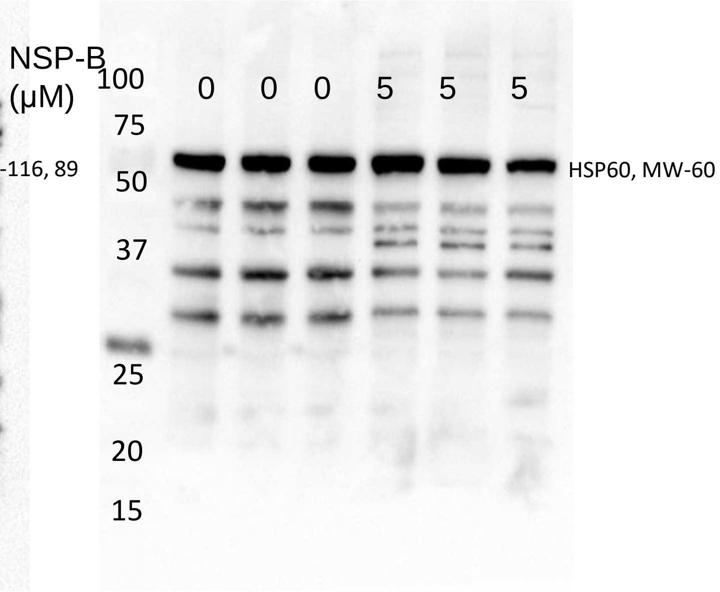

Supplement: Supplementary file 2 — Supporting information. [file CBIN-48-190-s007.pdf]
