# Supplementary figures and images for: Neosetophomone B induces apoptosis in multiple myeloma cells via targeting of AKT/SKP2 signaling pathway
Source: Cell Biol Int. 2023 Oct 26;48(2):190–200. doi: 10.1002/cbin.12101 (PMC10952688; doi:10.1002/cbin.12101)

Supplementary Figure 1

A

NSP-B ( $\mu\text{M}$ ) 0

0.1

0.5

1

2.5

5

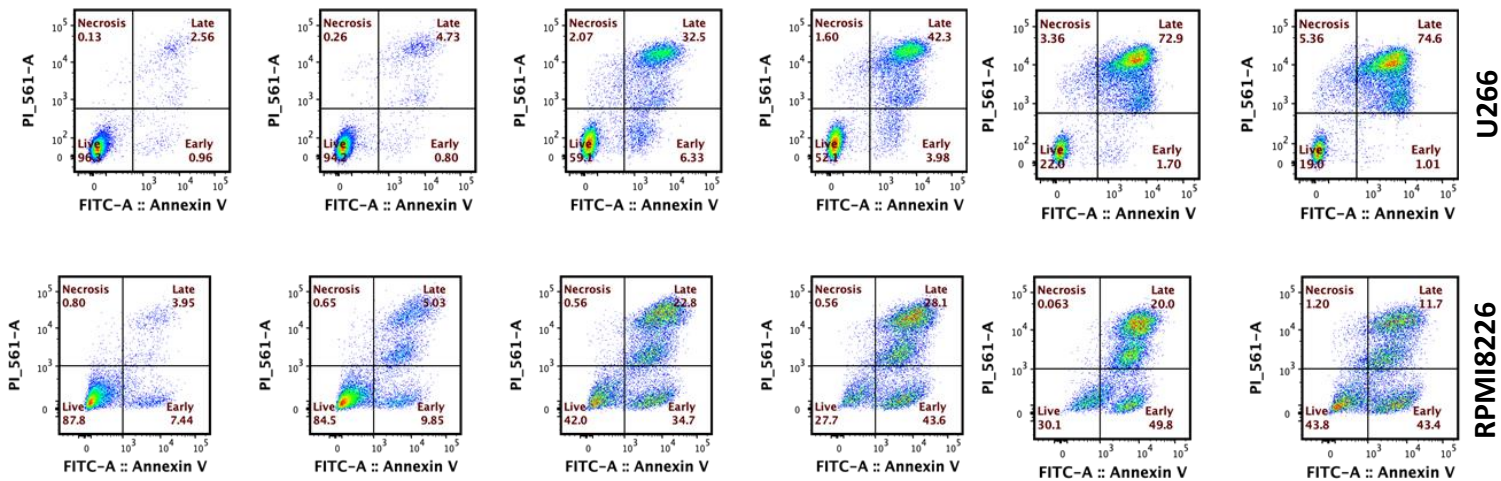

B

U266

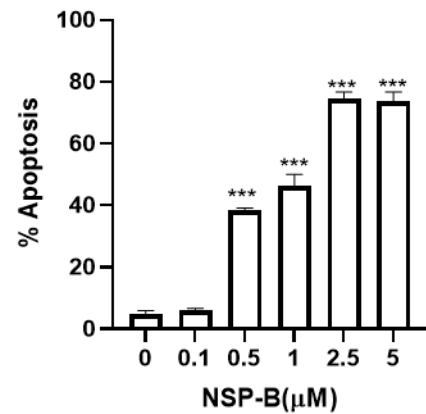

RPMI8226

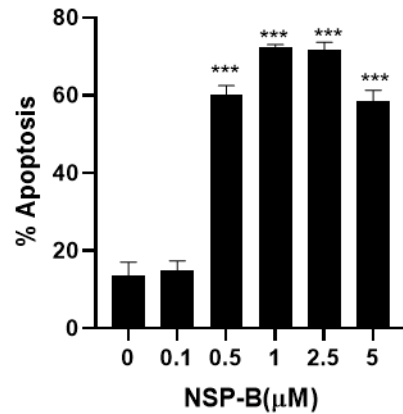

Supplement: Supplementary file 3 — Supplementary Figure 1: NSP‐B mediated apoptosis in MM cells.A,B) U266 and RPMI8226 cells were treated with lower doses of NSP‐B (0.1, 0.5,1, 2.5 and 5 µM), followed by staining with fluorescein‐conjugated annexin‐V/PI, and apoptotic cells were determined by flow cytometry. The graph displays the mean ± SD (standard deviation) of three independent experiments. *p < 0.05, **p < 0.01,***p < 0.001. [file CBIN-48-190-s002.pdf]

Supplementary Figure 2

**A**

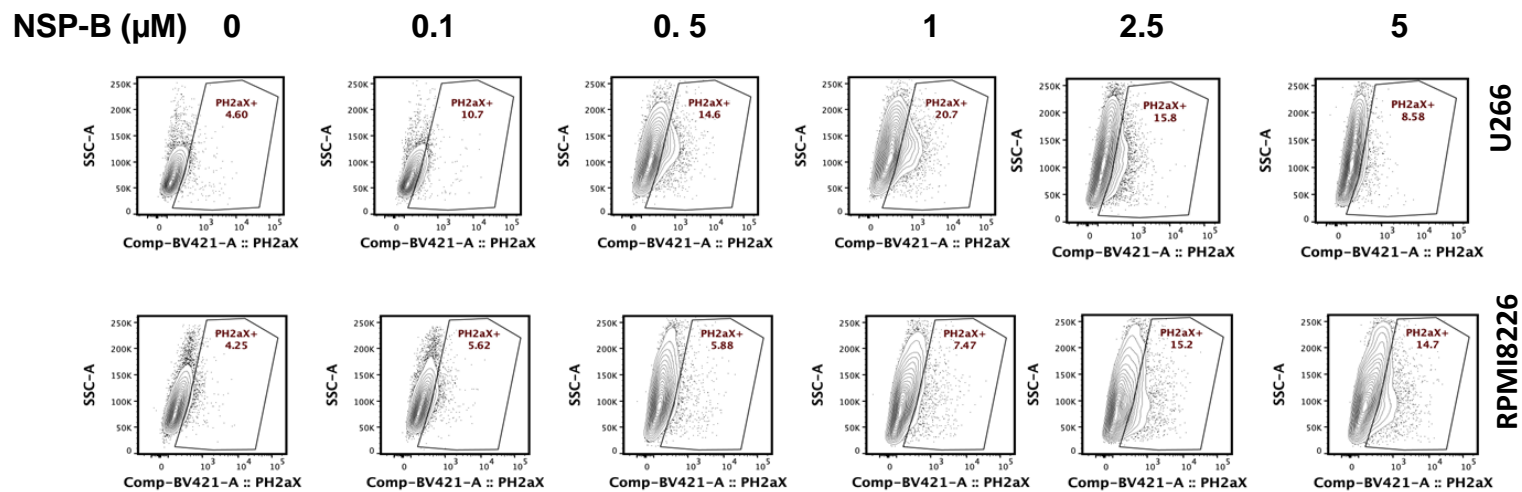

**B**

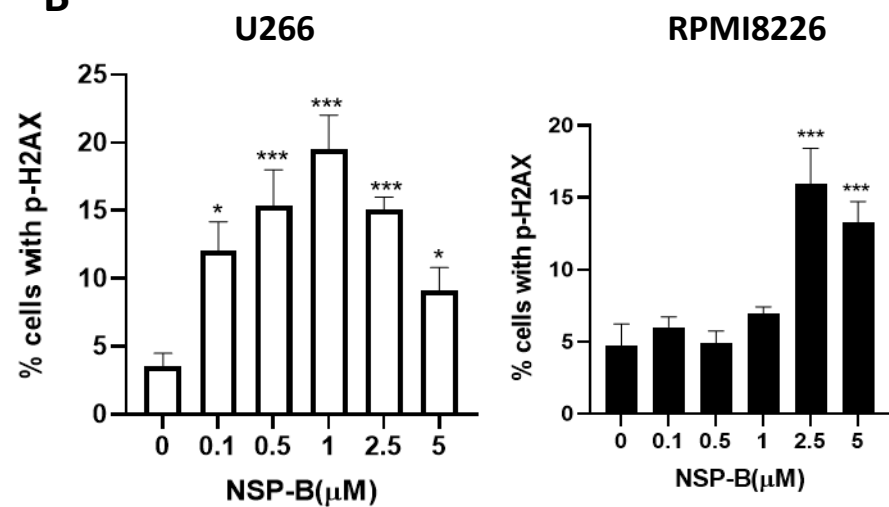

**C**

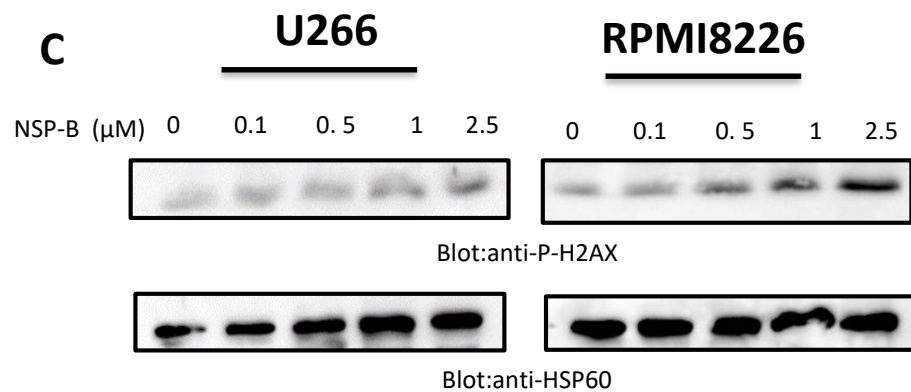

Supplement: Supplementary file 4 — Supplementary Figure 2. NSP‐B mediated phosphorylation of H2AX in MM cells.A,B)U266 and RPMI8226 cells were treated with NSP‐B and P‐H2AX were determined by flow cytometry. The graph displays the mean ± SD (standard deviation) of three independent experiments. *p < 0.05, **p < 0.01,***p < 0.001. C) U266 and RPMI8226 cells were treated with various doses of NSP‐B, and equal amounts of proteins were immuno‐blotted with antibodies against P‐H2AX and HSP60. Original Western blot images and quantification graphs can be found at Supplementary Files 1 and 2. [file CBIN-48-190-s009.pdf]

# Supplementary Figure 3

**A**

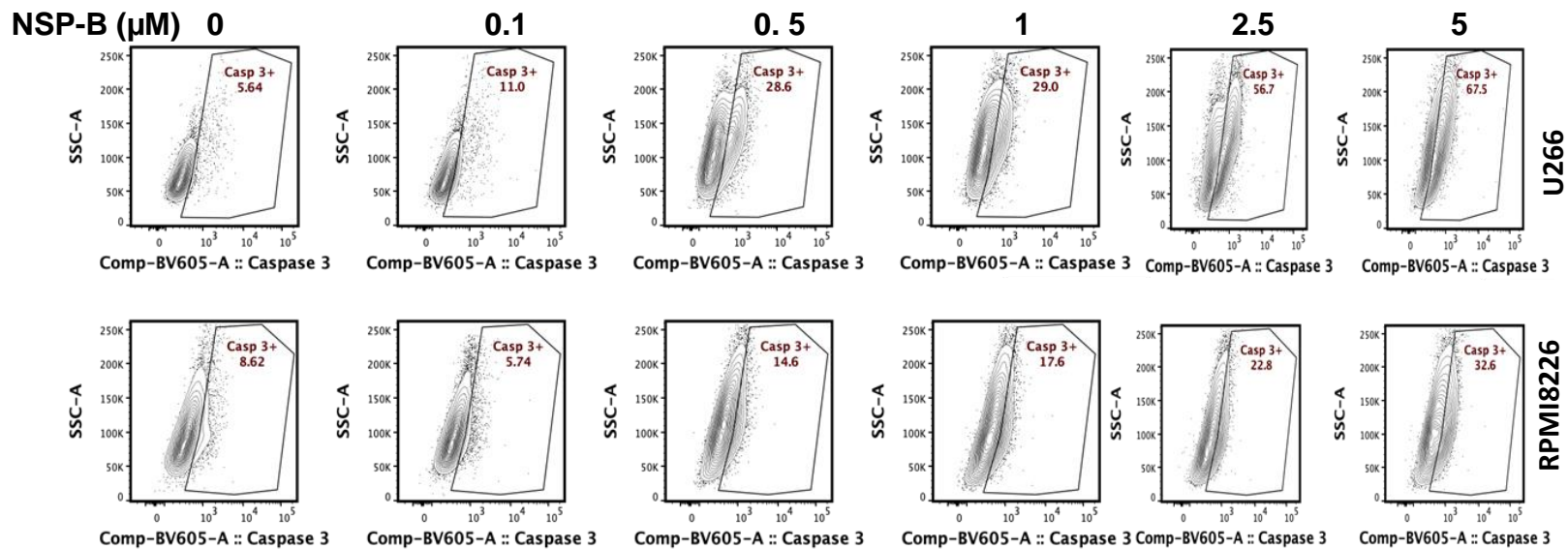

**C**

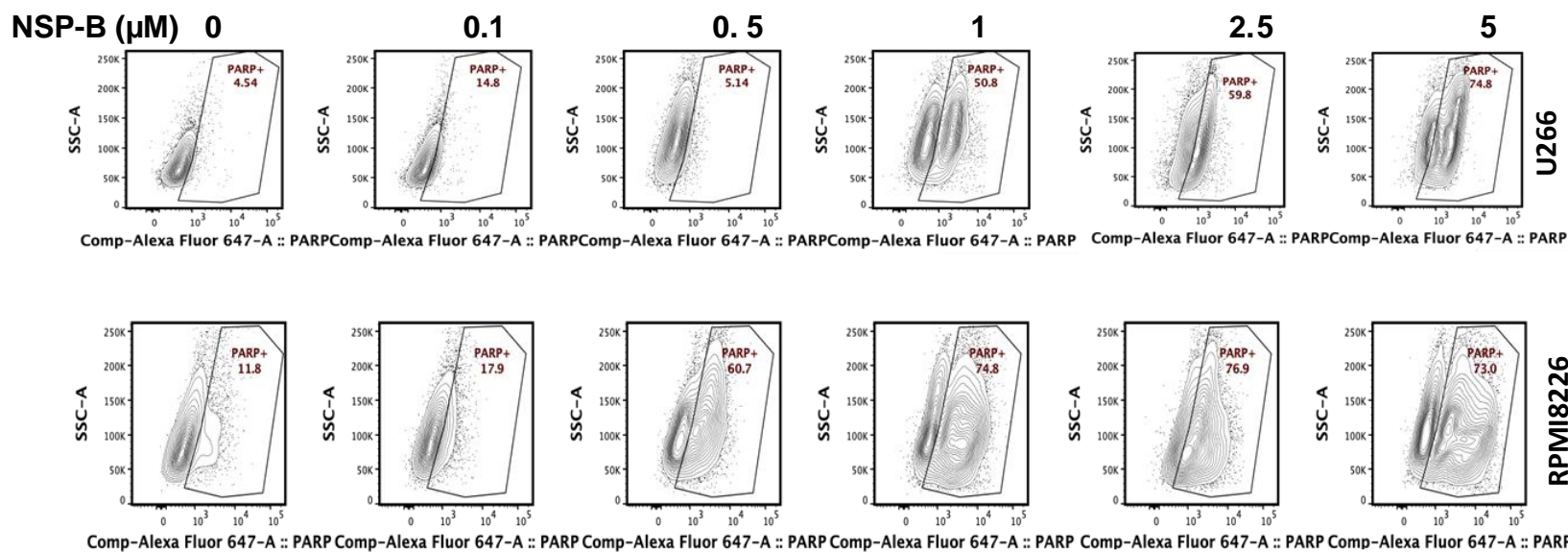

**B**

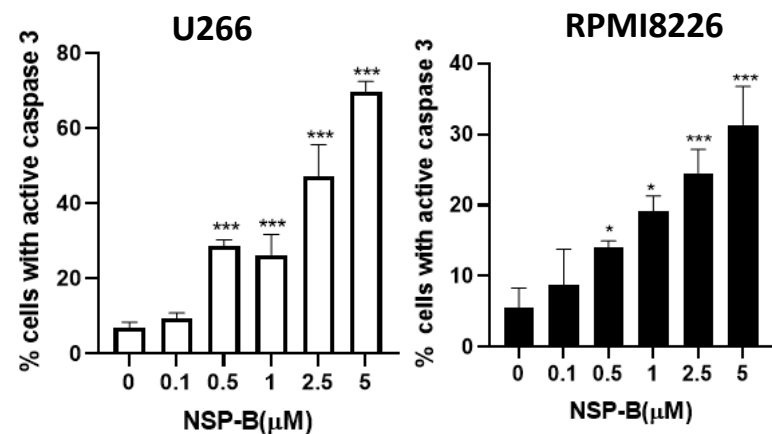

**D**

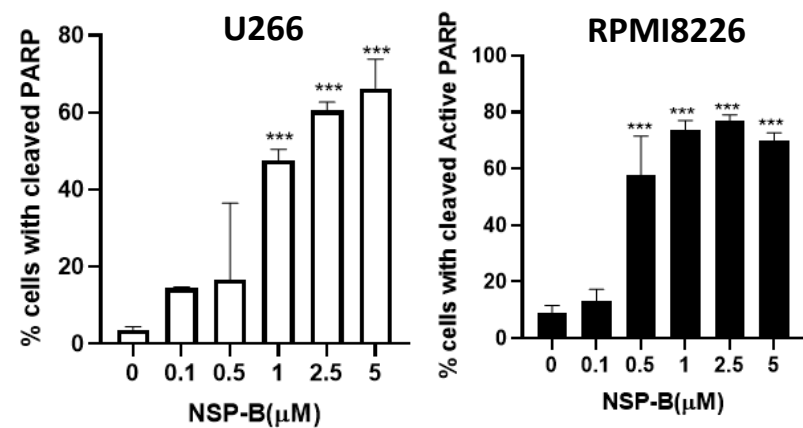

Supplement: Supplementary file 5 — Supplementary Figure 3: NSP‐B induced caspase and PARP activity in MM cells. A,B,C and D) U266 and RPMI8226 cells were treated with NSP‐B(0.1, 0.5,1, 2.5 and 5 µM) for 48 hours and caspase and PARP activity was determined by flow cytometry as described in Materials and Methods section. The graph displays the mean ± SD (standard deviation) of three independent experiments. *p < 0.05, **p < 0.01,***p < 0.001. [file CBIN-48-190-s005.pdf]

Supplementary Figure 4

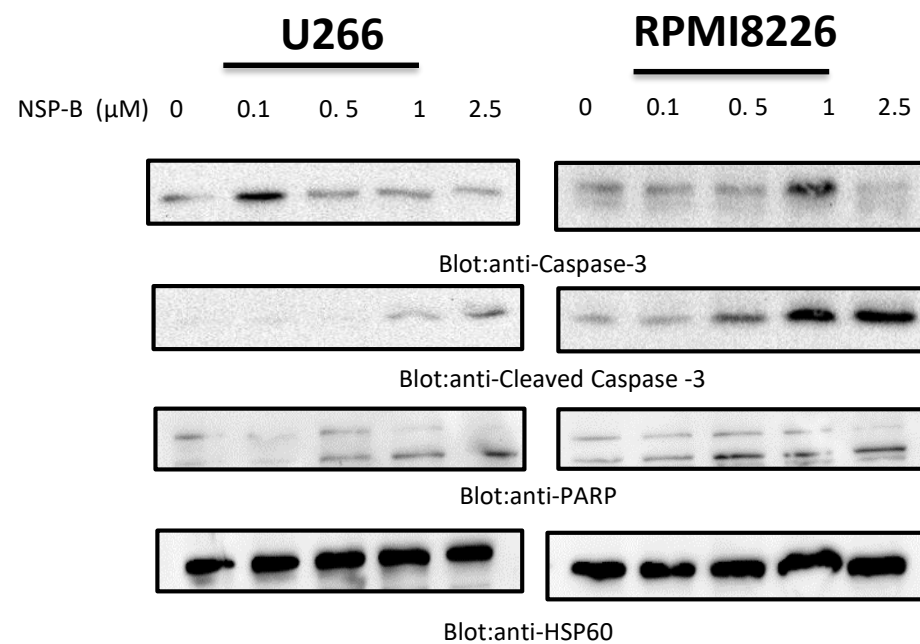

Supplement: Supplementary file 6 — Supplementary Figure 4: NSP‐B induced caspase and PARP activity in MM cells. U266 and RPMI8226 cells were treated with NSP‐B (0.1, 0.5,1, 2.5 and 5 µM) for 48 hours and equal amounts of proteins were immuno‐blotted with antibodies against Caspase‐3,cleaved caspase‐3. PARP and HSP60. Original Western blot images and quantification graphs can be found at Supplementary Files 1 and 2. [file CBIN-48-190-s001.pdf]

Supplementary Figure 5

A

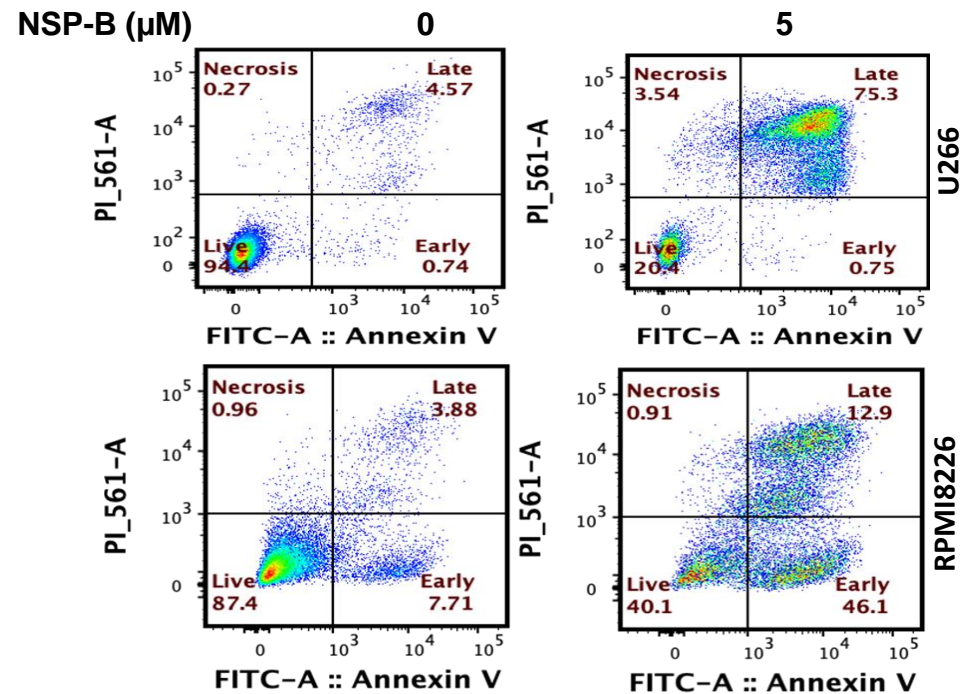

B

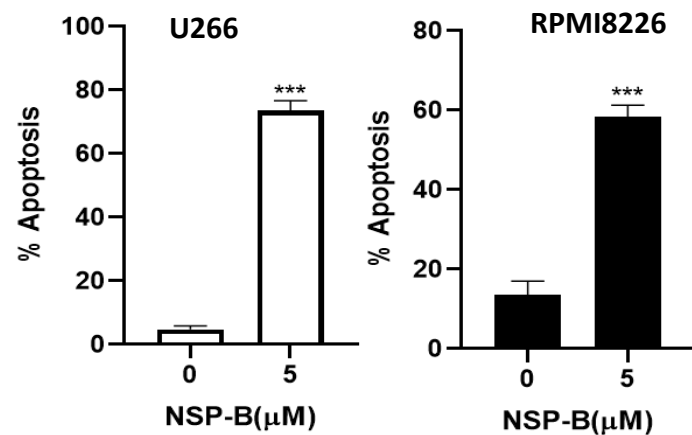

C

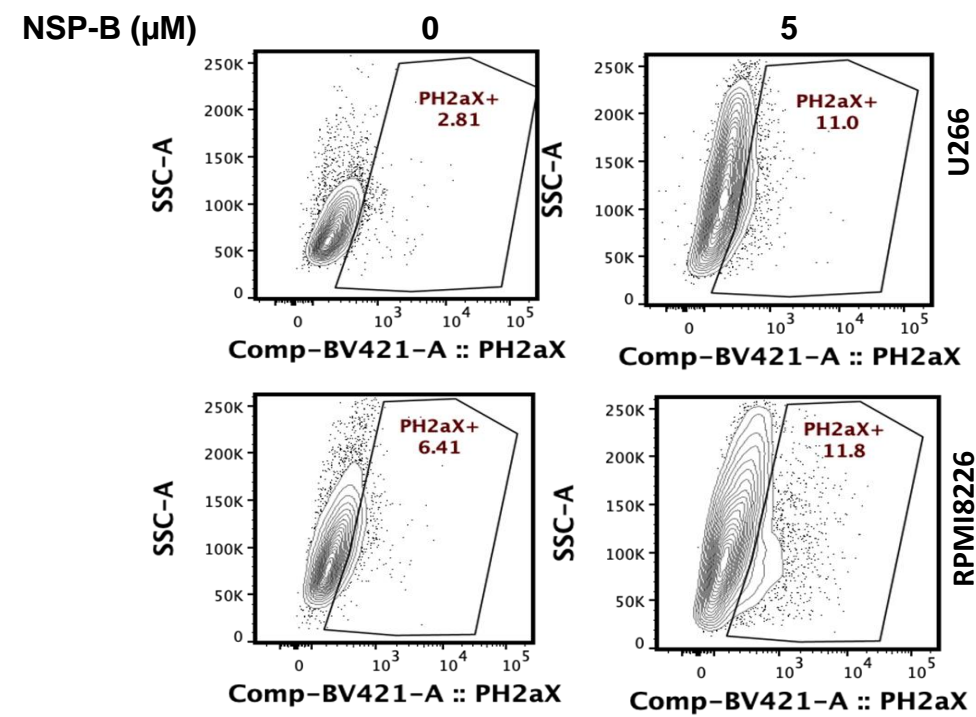

D

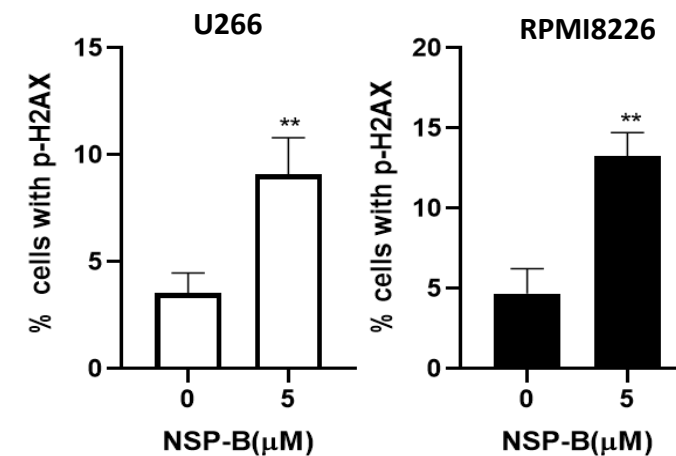

Supplement: Supplementary file 7 — Supplementary Figure 5: NSP‐B mediated apoptosis in MM cells.A,B) U266 and RPMI8226 cells were treated with and without NSP‐B (0 and 5 µM), followed by staining with fluorescein‐conjugated annexin‐V/PI, and apoptotic cells were determined by flow cytometry. The graph displays the mean ± SD (standard deviation) of three independent experiments. *p < 0.05, **p < 0.01,***p < 0.001. NSP‐B mediated phosphorylation of H2AX in MM cells.C,D) U266 and RPMI8226 cells were treated with and without NSP‐B (0 and 5 µM) and P‐H2AX were determined by flow cytometry. The graph displays the mean ± SD (standard deviation) of three independent experiments. *p < 0.05, **p < 0.01,***p < 0.001. [file CBIN-48-190-s004.pdf]

Supplementary Figure 6

A

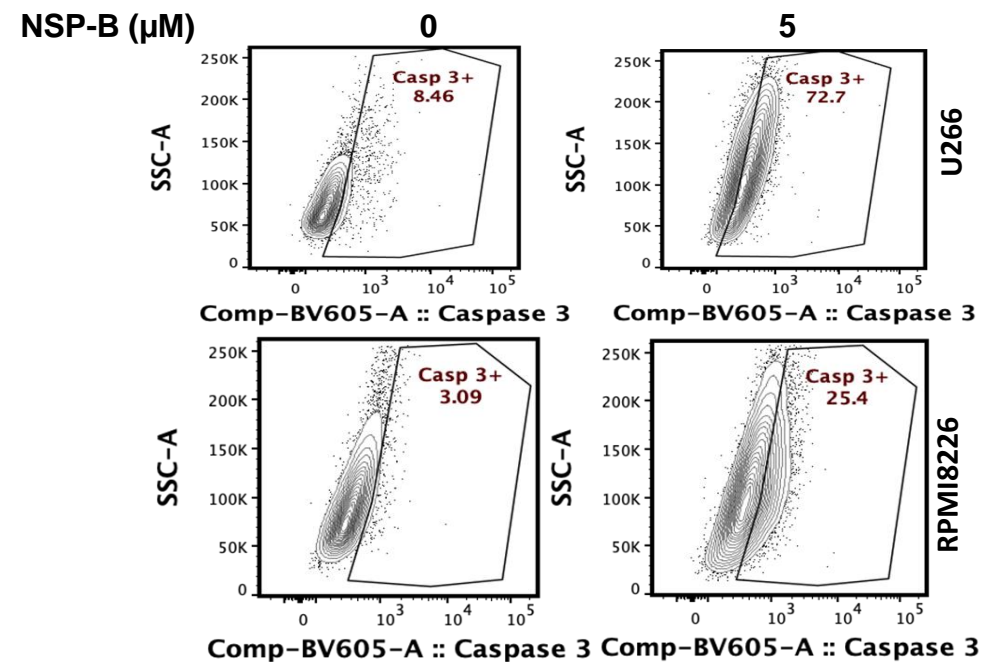

B

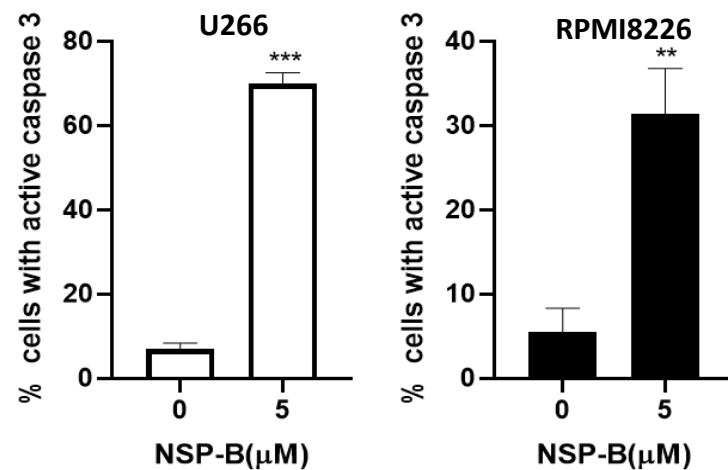

C

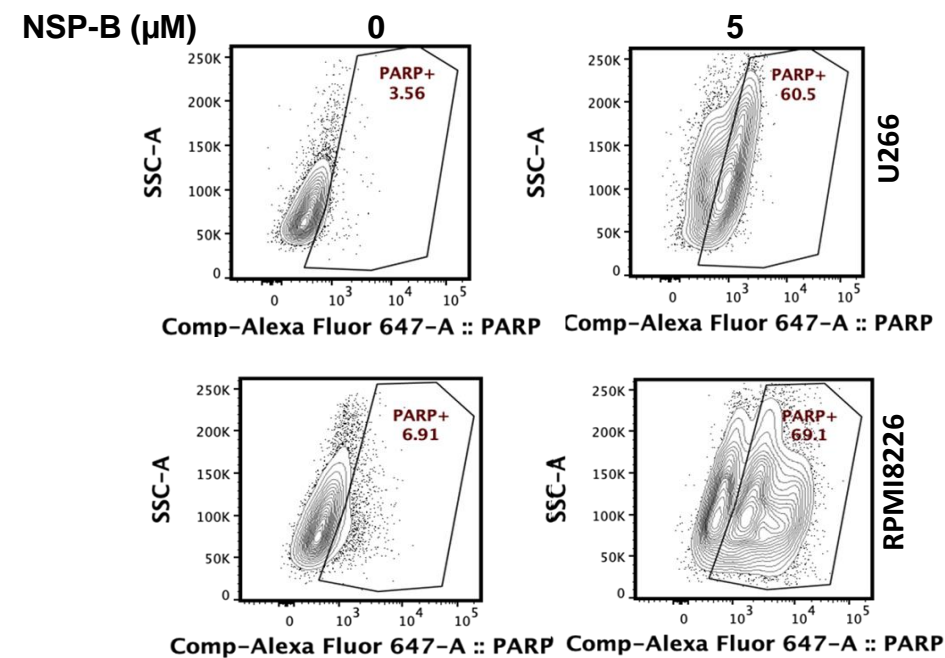

D

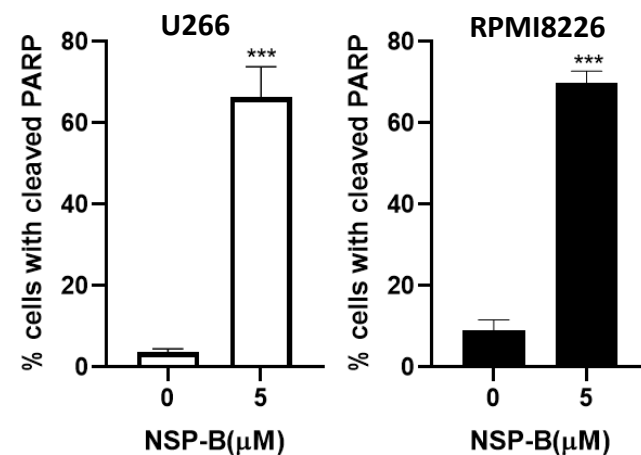

Supplement: Supplementary file 8 — Supplementary Figure 6: NSP‐B induced caspase and PARP activity in MM cells. A,B,C and D) U266 and RPMI8226 cells were treated with and without NSP‐B(0 and 5 µM) for 48 hours and caspase and PARP activity was determined by flow cytometry as described in Materials and Methods section. The graph displays the mean ± SD (standard deviation) of three independent experiments. *p < 0.05, **p < 0.01,***p < 0.001. [file CBIN-48-190-s006.pdf]

Supplementary Figure 7

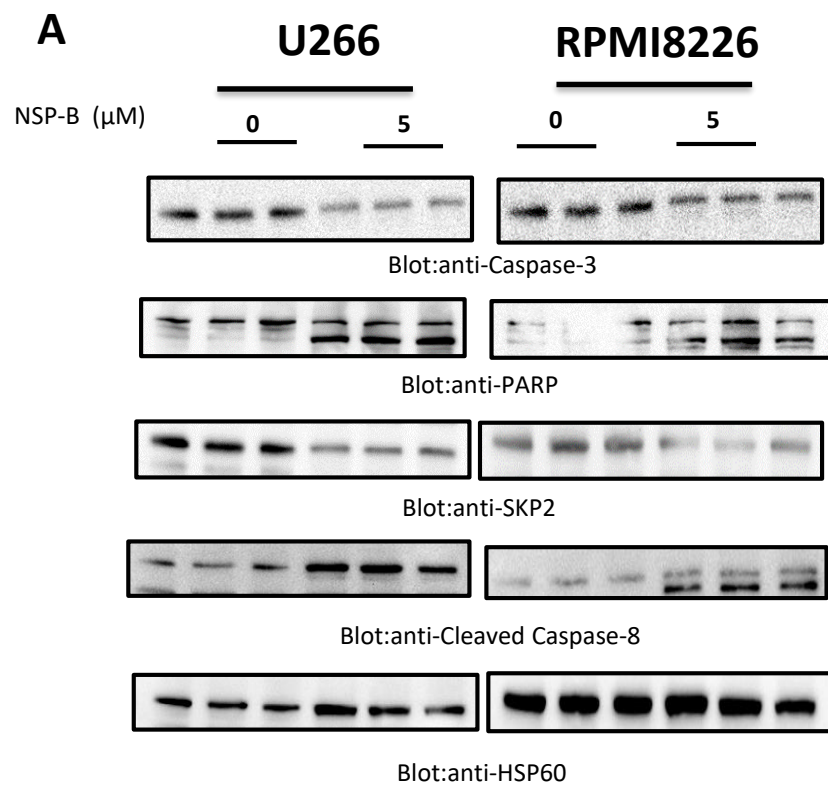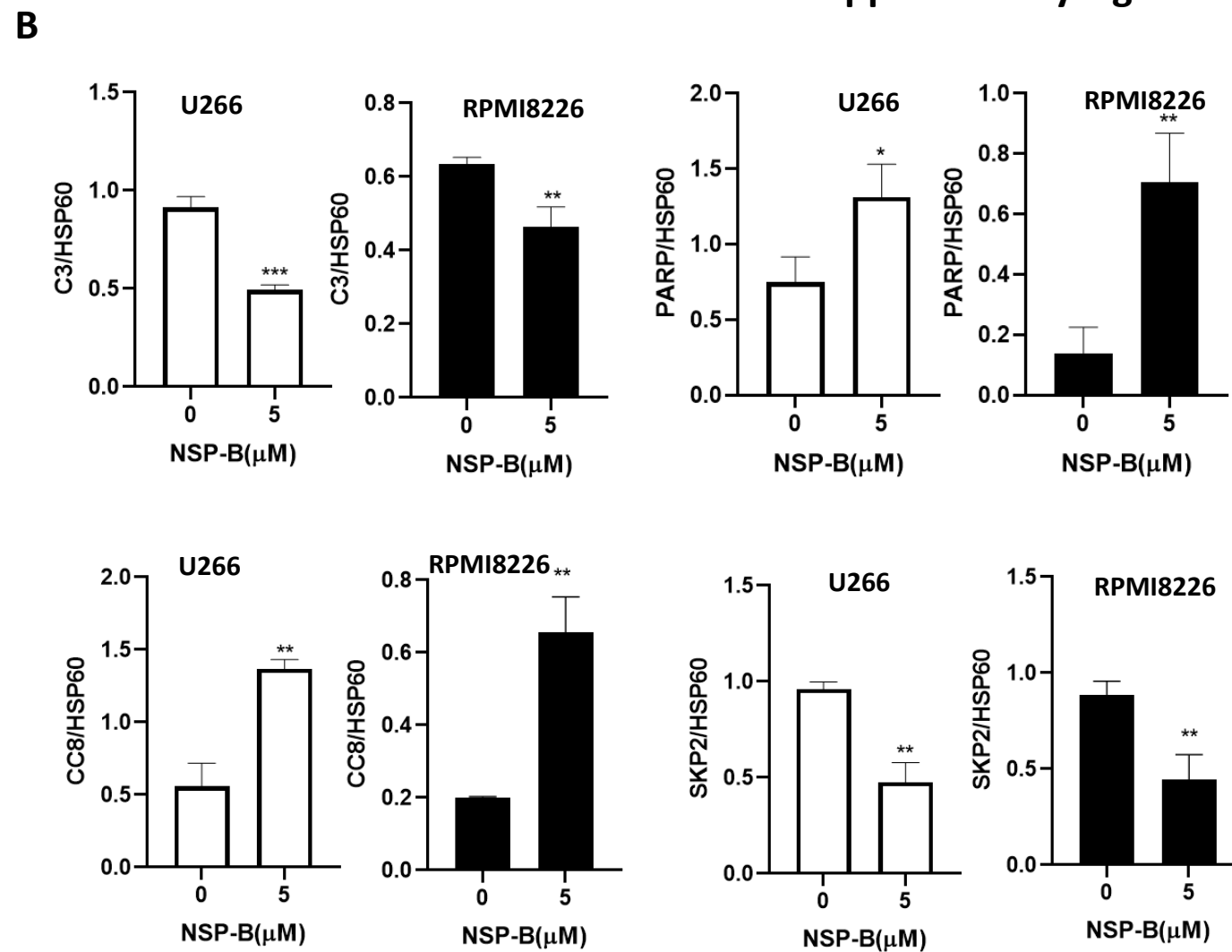

Supplement: Supplementary file 9 — Supplementary Figure 7: NSP‐B induced the activation of caspases and downregulated the expression of SKP2.A) U266 and RPMI8226 cells were treated with and without NSP‐B (0 and 5 µM) for 48 hours and equal amounts of proteins were immuno‐blotted with antibodies against Caspase‐3,PARP, SKP2,cleaved caspase‐8 and HSP60. Original Western blot images can be found at Supplementary File 2. B) The blots were quantified against house keeing gene (HSP60).The graph displays the mean ± SD (standard deviation) of three independent experiments. *p < 0.05, **p < 0.01,***p < 0.001. [file CBIN-48-190-s008.pdf]
